# Unimer Exchange as a Tool for Programming Enzymatic Degradation through Micellar Dynamics

**DOI:** 10.1021/acs.biomac.5c00955

**Published:** 2025-10-10

**Authors:** Shahar Tevet, Michal Brodsky, Roey J. Amir

**Affiliations:** † Department of Organic Chemistry, School of Chemistry, Faculty of Exact Sciences, 26745 Tel-Aviv University, Tel-Aviv 6997801, Israel; ‡ The Center for Nanoscience and Nanotechnology, Tel-Aviv University, Tel-Aviv 6997801, Israel; § ADAMA Center for Novel Delivery Systems in Crop Protection, Tel-Aviv University, Tel-Aviv 6997801, Israel

## Abstract

A key challenge in designing enzyme-responsive micellar nanocarriers lies in balancing their stability and enzymatic degradation. While it has been widely assumed that the micelle–unimer exchange governs enzyme accessibility to the hydrophobic blocks, this relationship had not been directly demonstrated. Here, to uncover this long-assumed mechanistic link, we synthesized a set of triblock amphiphiles that convert by an in situ transition to diblock amphiphiles via reductive cleavage of a central disulfide bond. In parallel, hydrophobicity was independently tuned by modifying the aliphatic end-groups. Enzymatic degradation studies and Förster resonance energy transfer (FRET)-based exchange assays showed two consistent trends across all systems: increasing hydrophobicity led to slower micelle–unimer exchange and reduced enzymatic degradation rates, while transition to diblock consistently enhanced both. These results provide direct evidence that exchange kinetics govern enzymatic degradation and lay the mechanistic foundation for overcoming the stability–degradability barrier for enzyme-responsive micelles by applying architectural transitions as a molecular programming tool.

## Graphical abstract

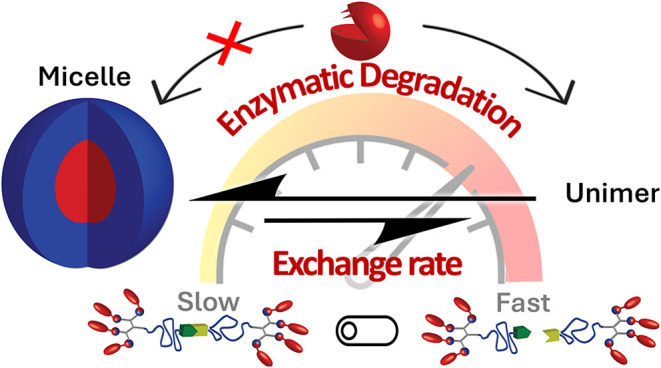

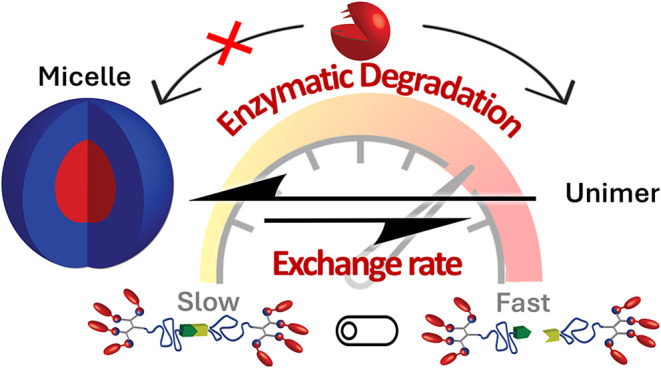

## Introduction

Polymeric micelles have gained considerable attention over the past decades due to their great potential in various biomedical applications, especially as platforms for targeted delivery of therapeutics and imaging agents.^1–9^ Due to their nature, amphiphilic block copolymers tend to self-assemble into core–shell nanostructures with hydrophobic cores and hydrophilic coronas, enabling effective encapsulation of poorly soluble drugs while enhancing their solubility, stability, and bioavailability.^10,11^

To enhance therapeutic efficacy and reduce systemic toxicity, considerable attention has been directed toward the development of stimuli-responsive polymeric micelles that can release their payload selectively at the target site.^12–15^ These ‘smart’ assemblies are engineered to undergo structural or functional changes in response to specific stimuli, such as light,^16,17^ pH,^18–20^ temperature,^21–23^ or enzymatic activity.^24–27^ Upon exposure to such stimuli, the physicochemical properties of the amphiphiles are altered, often leading to micelle disassembly, which plays a crucial role in controlled release and targeted therapy.^28,29^ The ability to fine-tune these systems to respond selectively to chemical and biological cues is critical for fulfilling their potential as advanced bioresponsive materials.

Among the various types of stimuli, enzymes stand out due to their high specificity and the overexpression of specific enzymes in disease-associated tissues.^12,25,30,31^ These characteristics make enzymes particularly suitable for selectively triggering the disassembly of polymeric assemblies, such as micellar nanocarriers, thereby enabling site-specific activation for targeted therapeutic applications.^32,33^ However, a key challenge in designing such systems is the need to balance micellar stability, which is required for their administration and circulation, with efficient enzymatic degradability at the target site. The rate of micelle degradation can significantly affect the timing and location of drug release, impacting the overall therapeutic outcomes. This significant challenge arises from the fact that the enzymatic substrates are often located within the hydrophobic core of the micelle, which is generally inaccessible to the activating enzyme, making the interaction between enzymes and polymeric micelles fundamentally different from that of other stimuli. Unlike dimensionless stimuli such as light and temperature or small-molecule-based triggers such as pH changes and oxidative-responsive systems,^34,35^ enzymes are macromolecular entities whose sizes are comparable to those of polymeric micelles. Consequently, their ability to penetrate through the hydrophilic shell and access the hydrophobic core of micelles is significantly hindered. This restricted access implies that enzymatic degradation of polymeric micelles cannot occur directly within the hydrophobic core of the assembled state but rather through an equilibrium-driven mechanism. Specifically, enzymes interact predominantly with the free unimeric form of amphiphilic polymers that exist in equilibrium with their micellar counterparts. The rate of enzymatic degradation is thus believed to be governed by the micelle–unimer equilibrium as micelles with increased stability show lower susceptibility to enzymatic degradation, and vice versa.^36,37^ The need to balance between the stability and responsiveness of the assembled system adds another layer of complexity to the design and application of enzyme-responsive polymeric assemblies, making this equilibrium-based mechanism a key factor for designing polymeric nanocarriers with tunable stability and responsiveness.

To further understand the balance between stability and enzymatic degradability, several groups including the Thayumanavan group^38–41^ and ours^24–27^ have used critical micelle concentration (CMC) values, which describe the inherent tendency of amphiphilic polymers to self-assemble into micelles, to correlate the thermodynamic stability of the micellar system with their enzymatic degradation rates. However, while higher CMC values indeed correlated with faster enzymatic degradation, the changes in CMC values, which were often rather minor, failed to capture the wide range of enzymatic degradation rates, often ranging from rapidly degrading amphiphiles to nondegradable ones.^42^ In contrast to the thermodynamically based CMC values, which are routinely measured and provide information on the effective concentration of unimers in the solution, the kinetic stability of a micellar system refers to the ability of micelles to retain their structure over time and resist disassembly under dynamic conditions and is usually unexplored when studying enzymatic degradation of amphiphilic assemblies. Hence, due to the gap between the differences in CMC values, which reflect the concentration of accessible unimers, and the enzymatic degradation rates, which are expected to be strongly influenced by the rate at which unimers are expelled from the micelles and become available to interact with the enzymes, we believe it is essential to consider the kinetics of micelle–unimer exchange as the missing link between stability and degradability.

The micelle–unimer chain exchange rate serves as a key indicator of kinetic stability, reflecting the frequency at which individual polymer chains dissociate from micelles, diffuse through the solvent, and reassociate with other micelles.^43–49^ This exchange, described by the Aniansson–Wall model,^50^ depends heavily on the energy barrier for chain expulsion. A range of experimental techniques, such as time-resolved small-angle neutron scattering (TR-SANS),^51^ fluorescence correlation spectroscopy (FCS),^52^ and Förster resonance energy transfer (FRET),^53–55^ have been used to probe these dynamics. This dynamic exchange process should therefore play a critical role in determining how micelles respond to enzymatic stimuli and how accessible their hydrophobic cores are to enzymatic interactions.

Various structural parameters have been shown to influence micelle–unimer exchange dynamics. These parameters include the molecular weight of the amphiphiles, the polymer rigidity, and their architectural configuration, in addition to the hydrophobicity of the core of the micelles.^56–60^ For example, high-molecular-weight polymers or strongly bound hydrophobic cores typically yield kinetically stable micelles with reduced chain exchange rates. Conversely, amphiphiles with lower molecular weight or reduced hydrophobicity exhibit faster exchange and dynamic reorganization.^45^ The architectural design of amphiphilic polymers also plays a critical role. For example, triblock amphiphiles often form more kinetically stable micelles, as, unlike diblock amphiphiles (DBAs), in order for a triblock amphiphile to leave the micelle, it needs both its hydrophobic blocks to escape the hydrophobic core and pass through the hydrophilic shell, causing an overall slower unimer exchange rate than diblock counterparts.^58^

While the impact of the exchange rate on enzymatic degradation has long been proposed, despite the growing recognition of this relationship, experimental systems that allow direct and controlled dissection of these dynamics have been lacking. This gap has limited progress toward true mechanistic insight and a predictive and quantitative understanding, which remain elusive. Establishing this link is essential for advancing the fundamental understanding of how nanoscale dynamic behavior governs enzyme accessibility. Moreover, clarifying how unimer availability dictates enzymatic degradability of the hydrophobic domains of polymeric amphiphiles is critical for overcoming the inherent stability–responsiveness barrier in enzyme-responsive micelles. This deeper understanding could ultimately unlock new strategies for designing next-generation micellar nanocarriers with programmable degradation profiles, selectivity, and optimized therapeutic performance.

Recently,^61^ we have developed a general and modular approach for designing stable enzyme-responsive micelles whose enzymatic degradation can be enhanced on demand, aiming to overcome the stability–responsiveness barrier of such systems. The control over their response to the activating enzyme was achieved by stimuli-induced splitting of triblock amphiphiles (TBAs) into two identical diblock amphiphiles (DBAs), which have the same hydrophilic–lipophilic balance as the parent triblock amphiphile and exactly half the molecular weight. While this architectural change did not alter micelle size or thermodynamic properties, the sensitivity of the micelles toward enzymatic degradation, protein interactions, and cargo release were all drastically affected. We attributed this correlation to a change in the micelle–unimer equilibrium in situ. As a proof of concept, we designed both UV- and reduction-activated splitting mechanisms, demonstrating the ability to use architectural transition as a tool for tuning amphiphile–protein interactions, providing a general solution toward overcoming the stability–degradability barrier for enzyme-responsive nanocarriers.

Herein, we aim to apply our modular design strategy to directly test the hypothesis regarding the correlation between enzymatic degradation and micelle–unimer exchange rate using dendritic triblocks. We specifically aimed to understand how three key molecular parameters—hydrophobicity, molecular weight, and architecture—influence both degradation and micellar dynamics. To do so, we used our previously developed splittable amphiphile platform, which enables an in situ architectural transition from TBA to DBA through reductive cleavage. This approach allows clean decoupling of structural variables: the in situ disulfide-based splitting simultaneously alters architecture and molecular weight, while hydrophobicity is tuned independently through the introduction of different aliphatic end-groups during the synthesis of the dendrons.

Enzymatic degradation was tracked by monitoring the hydrolysis of the amphiphiles over time using HPLC. In parallel, micelle–unimer exchange dynamics were evaluated using a FRET-based mixing assay, in which micellar populations were separately labeled with complementary fluorophores and exchange was assessed by monitoring the emergence of FRET signal upon mixing. These two complementary experiments were performed across six amphiphilic micelles systematically varying in architecture and hydrophobicity. As conceptually illustrated in Figure 1, these measurements were designed to test whether faster unimer exchange increases enzyme accessibility and, consequently, degradation. By analyzing both enzymatic hydrolysis and micellar mixing across structurally defined systems, this study aims to provide direct experimental evidence for the mechanistic link between micellar exchange and enzymatic responsiveness.

**Figure 1:**
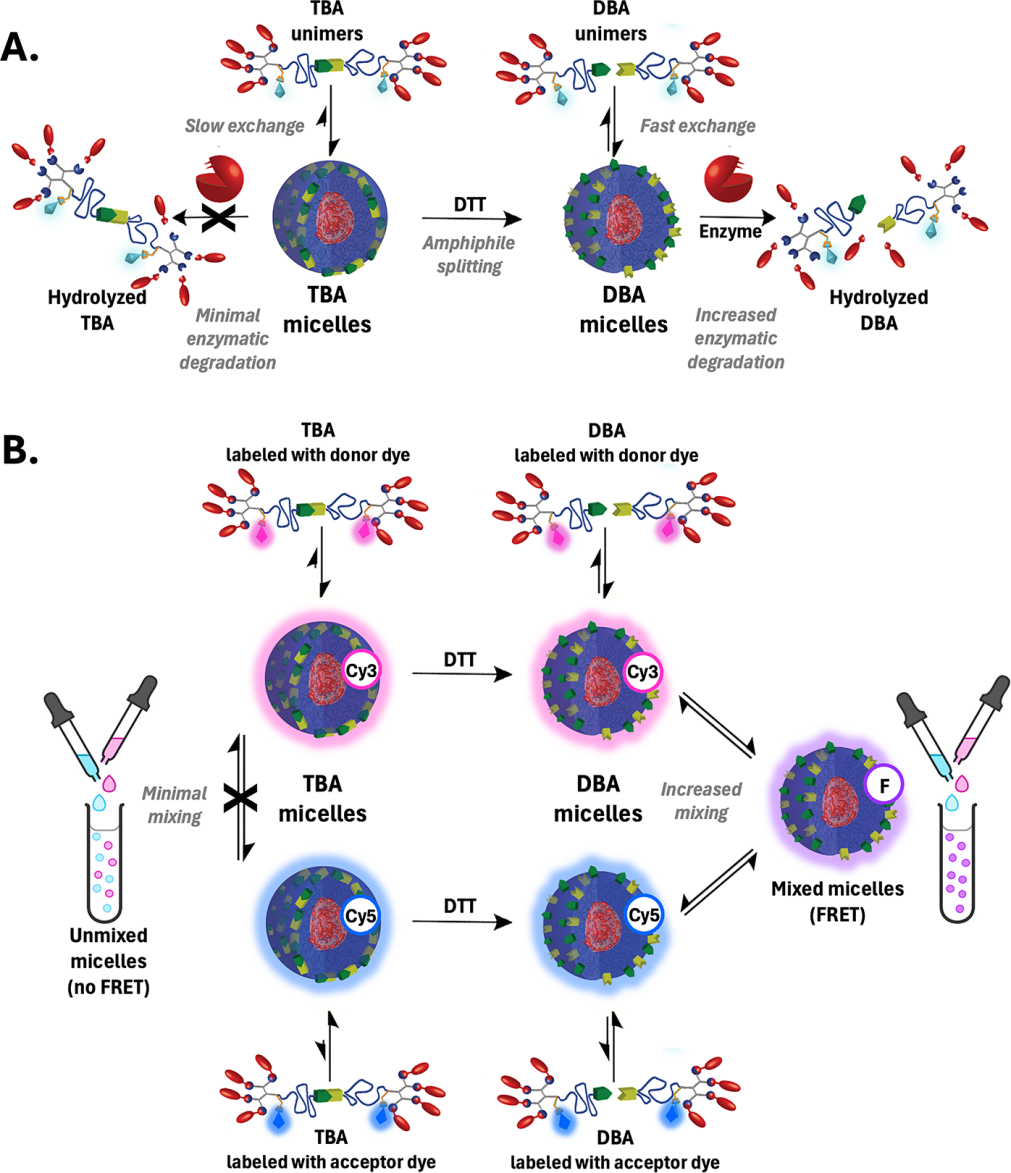
Conceptual illustration of experimental approaches used to assess the relationship between micelle–unimer exchange dynamics and enzymatic degradation. (A) Schematic of enzymatic degradation experiments: TBAs undergo in situ DTT-induced splitting to DBAs, resulting in faster micelle–unimer exchange and increased enzymatic susceptibility. (B) Schematic of FRET-based exchange experiments: upon spontaneous mixing of Cy3- and Cy5-labeled micellar populations, the TBA system remains unmixed (pink and blue), while DBA micelles rapidly exchange unimers, forming uniformly mixed populations (purple), indicative of faster exchange.

## Results and Discussion

### Molecular Design, Synthesis, and Characterization

Our approach is based on using stimuli-induced architectural transitions of hydrophobic–hydrophilic–hydrophobic (B–A–B) triblock amphiphiles into hydrophobic–hydrophilic (B–A′) diblock amphiphiles as a tool for enhancing the enzymatic degradation of their hydrophobic blocks. This in situ transition strategy allows clean structural modification without altering formulation conditions, ensuring that observed effects on degradation and exchange reflect only changes in the architecture and molecular weight.

The synthetic design of the splittable amphiphiles is presented in Scheme 1. We chose poly­(acrylic acid) (PAA) as the hydrophilic block (A) and implemented a redox-responsive linker at its center. As shown previously, this linker will allow us to achieve the macromolecular transition from TBA to DBA in the presence of a reducing agent, DTT (Scheme 1B). PAA was selected due to its relatively high hydrophilicity, which should facilitate the formation of micelles rather than hydrogels that are often obtained for other PEG-based triblock systems. The synthesis of this block was performed as previously reported,^61^ starting by atom transfer radical polymerization (ATRP) of *tert*-butyl acrylate (tBA) from a bifunctional initiator. This initiator also serves as the redox-responsive splittable linker that bears a disulfide bond in its center. After polymerization, the terminal bromides were substituted into azides, which were conjugated by copper-catalyzed azide–alkyne cycloaddition (CuAAC)^62^ to the hydrophobic dendrons.

**Scheme 1:**
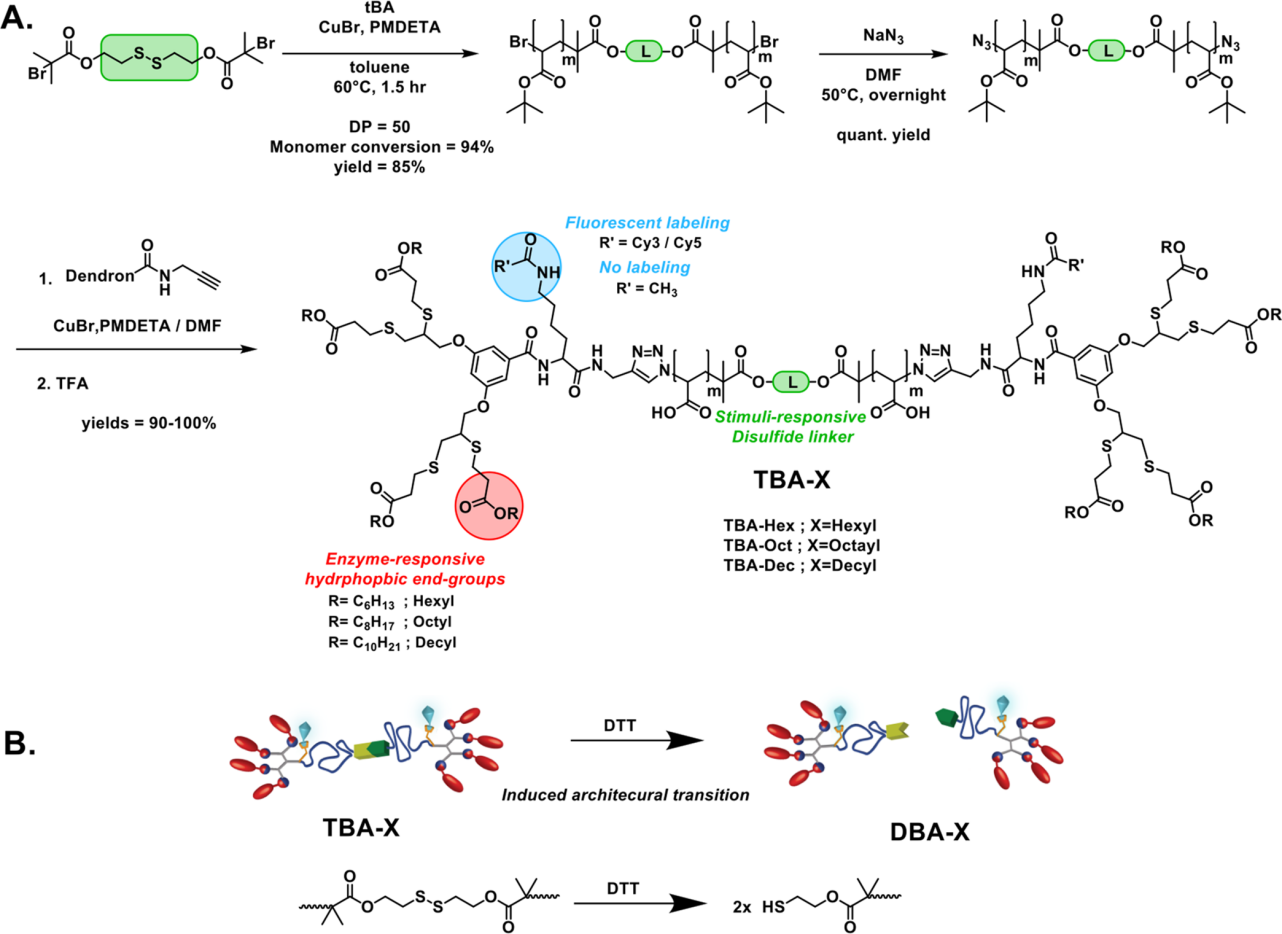
Molecular Design and Synthesis of Enzyme-Responsive Splittable Triblock Amphiphiles; (A) Synthetic Route to TBA-X; the Central Redox-Responsive Disulfide Linker Enables In Situ Splitting into Two Diblock Amphiphiles (DBA-X); the Three Amphiphile Families Differ in Hydrophobicity, Modulated by Aliphatic End-Groups (Hex, Oct, or Dec). Fluorophore Labels (Cy3 and Cy5) Were Incorporated through a Lysine-Based Linker for FRET Analysis; (B) Schematic Illustration of the Stimuli-Induced Architectural Transition from TBA to DBA in the Presence of the Reducing Agent DTT

These enzyme-responsive dendrons, serving as the hydrophobic blocks (B), contain four aliphatic end-groups, which are linked through ester bonds and can hence serve as hydrophobic substrates for a model enzyme, porcine liver esterase (PLE). The dendritic architecture offers high molecular precision and tunable hydrophobicity, controlled by adjusting the length of the aliphatic end-groups. For this study, we have used hexyl-, octyl-, and decyl-terminated dendrons, enabling systematic modulation of amphiphiles' hydrophobicity across three TBA families. Together with our splittable architecture, this platform allows independent modification of molecular weight, architecture, and hydrophobicity—three key determinants of micellar behavior.

Utilizing a lysine linker embedded in the hydrophobic block, the dendrons were fluorescently labeled with either Cy3 or Cy5, serving as a chromophore pair suitable for FRET measurements. Förster resonance energy transfer (FRET) involves the nonradiative transfer of excitation energy from an excited donor fluorophore (Cy3) to a proximal ground-state acceptor fluorophore (Cy5). It is susceptible to nanometer-scale changes in donor–acceptor separation distance and their relative dipole orientations.^63^ Since FRET is highly dependent on the proximity of the dyes, only in a mixed micelle containing both the donor and acceptor will this spectral phenomenon be observed. Hence, the combination of FRET dye pairs as part of the molecular design will serve as a spectral report mechanism for the unimer exchange rate (Figure 1B). Monitoring the fluorescence emission will afford kinetic analysis of the mixing of the two micellar populations, each labeled with a different fluorophore, which is directly affected by the micelle–unimer equilibrium and the rate of unimer exchange with the outer environment. To minimize dye–dye quenching, additional dendrons labeled with acetamide were used to dilute the fluorophores within the micellar formulation.

Overall, nine splittable TBAs were synthesized for this study, presenting three degrees of hydrophobicity and three types of labeling. All amphiphiles were obtained in high purity and characterized by NMR, HPLC, SEC, and UV–vis and fluorescence spectroscopies (see the Supporting Information).

To confirm the ability of the synthesized amphiphiles to form micellar assemblies in aqueous media, we first characterized the self-assembly of the TBAs under physiological conditions (PBS, pH 7.4). To address the different polymeric micelles used in this study, the systems will be named using the amphiphile architecture (TBA/DBA) and the associated end-groups (X). All three TBAs, TBA-Hex, TBA-Oct, and TBA-Dec, were found to spontaneously self-assemble into stable micelles. CMC values were determined using FRET analysis,^64^ while dynamic light scattering (DLS) and transmission electron microscopy (TEM) confirmed the formation of spherical nanoscale assemblies with consistent diameters and low polydispersity (Figures S48–S50). These measurements verified that all three TBA variants formed well-defined micelles across the tested hydrophobic range. Following the in situ conversion of TBA to DBA via DTT treatment, the corresponding DBA-based systems were evaluated under identical conditions. CMC, DLS, and TEM measurements were repeated to assess their ability to self-assemble into micelles. The results (Table 1) showed that all DBAs also formed micelles of comparable size, confirming that the architectural transition does not disrupt assembly.

**Table 1:** Micellar Assemblies and Their Properties

amphiphile	end-group	Mn^a^ (kDa)	*D̵* ^b^	weight ratio^c^	CMC^d^ (μM)	*D*_H_ ^e^ (nm)	*D*_H_ ^f^ (nm)
TBA-Hex	hexyl	6.55	1.12	0.38	6 ± 1	11 ± 2	15 ± 3
DBA-Hex	hexyl	3.28		0.38	9 ± 1	9 ± 1	16 ± 3
TBA-Oct	octyl	6.78	1.12	0.40	5 ± 1	10 ± 1	16 ± 4
DBA-Oct	octyl	3.39		0.40	8 ± 1	10 ± 1	17 ± 4
TBA-Dec	decyl	7.01	1.13	0.42	4 ± 1	11 ± 1	15 ± 2
DBA-Dec	decyl	3.50		0.42	6 ± 1	11 ± 1	16 ± 3

a Calculated based on the hydrophilic SS-PAA block (4.0 kDa as calculated from ^1^H NMR) and the expected exact mass of the synthesized dendrons.

b Based on SEC analysis of the *t*-butyl-protected TBAs.

c Weight ratio of the dendritic block(s) within the amphiphiles.

d Determined using FRET between Cy3- and Cy5-labeled amphiphiles.

e Hydrodynamic diameter measured by DLS.

f Hydrodynamic diameter measured by TEM (average of at least 15 particles).

### Kinetic Experiments

Before evaluating the correlation between enzymatic degradation and micelle–unimer exchange across the six amphiphilic systems, we wished to reaffirm a central principle of this study: that the architectural transition from TBA to DBA can serve as a tool to enhance enzymatic degradability in situ. This mechanistic foundation, established in our previous study,^61^ highlights the dynamic tunability of these polymeric assemblies. Since the impact of architecture on enzymatic degradability serves as a key mechanistic tool for the comparisons made throughout this study, we first revalidated this effect under the present experimental setup using TBA-Hex as a representative system (Figure 2). Upon incubation with porcine liver esterase (PLE) under physiological pH at 37 °C, we monitored the molecular composition of the micellar solution by HPLC. Over time, a gradual decrease in the area under the curve (AUC) corresponding to TBA-Hex was observed along with the appearance of a new peak assigned to the hydrolyzed amphiphile (Figure 2A,B). These results confirm that, under these conditions, TBA-Hex undergoes slow enzymatic degradation, with 25% conversion over 6 h. To evaluate the effect of architecture on hydrolysis rate, we next introduced DTT to the system to trigger the cleavage of the central disulfide bond and convert TBA-Hex to DBA-Hex in situ. The full architectural transition from TBA to DBA was clearly shown by HPLC within a few minutes, as can be seen in Figure 2B. As expected, the transition was accompanied by a marked increase in the enzymatic degradation rate, reflected in a steeper decline in the parent peak in the HPLC chromatogram. This acceleration is attributed solely to the change in architecture and molecular weight, as the hydrophobic dendrons and overall HLB remain identical. Importantly, to confirm that DTT itself does not affect enzymatic reactivity, we conducted control experiments using a control diblock amphiphile in the presence and absence of DTT (Figure S51). No difference in degradation profiles was observed, indicating that the enhanced degradation observed after splitting is due solely to architectural change and not to the reductive environment. Moreover, in the absence of PLE, both TBA and DBA remained intact, and no hydrolysis was observed under the experimental conditions (Figure S52).

In parallel, we monitored the enzymatic degradation of the system using fluorescence spectroscopy. Mixed micelles were prepared by coassembling TBA-Hex amphiphiles labeled with 10% Cy3, 10% Cy5, and 80% unlabeled chains. These micelles exhibited a FRET signal under Cy3 excitation, confirming close proximity between the two dyes, as expected for coassembled micelles. As enzymatic degradation proceeded, FRET intensity decreased gradually, with a corresponding decrease in Cy5 emission and an increase in Cy3 signal, reflecting progressive micellar disassembly (Figure 2C,D). Following the DTT-induced transition to DBA-Hex, FRET decay occurred significantly faster, reinforcing the link among amphiphile architecture, enzymatic degradation, and micellar disassembly. Notably, in the absence of the activating enzyme, both with and without DTT, the micelles exhibited stable FRET intensity throughout the entire measurement time frame (Figure S53). This confirms that the observed emission changes are driven by micellar disassembly rather than photobleaching or spontaneous dye diffusion. Moreover, when performing HPLC analysis of the enzymatic degradation for this mixed-micelle formulation, Cy3-, Cy5-, and unlabeled amphiphiles can be monitored separately due to their distinctive wavelengths. Their degradation profiles were found to be comparable across all tested conditions, confirming that fluorescent labeling had no measurable effect on enzymatic degradability. In the main analysis, degradation was quantified using the Cy3-labeled population as a representative signal. Together, these results validate our ability to use FRET as a sensitive and efficient tool for monitoring real-time micellar disassembly and underscore the role of architecture in enzymatic responsiveness.

**Figure 2:**
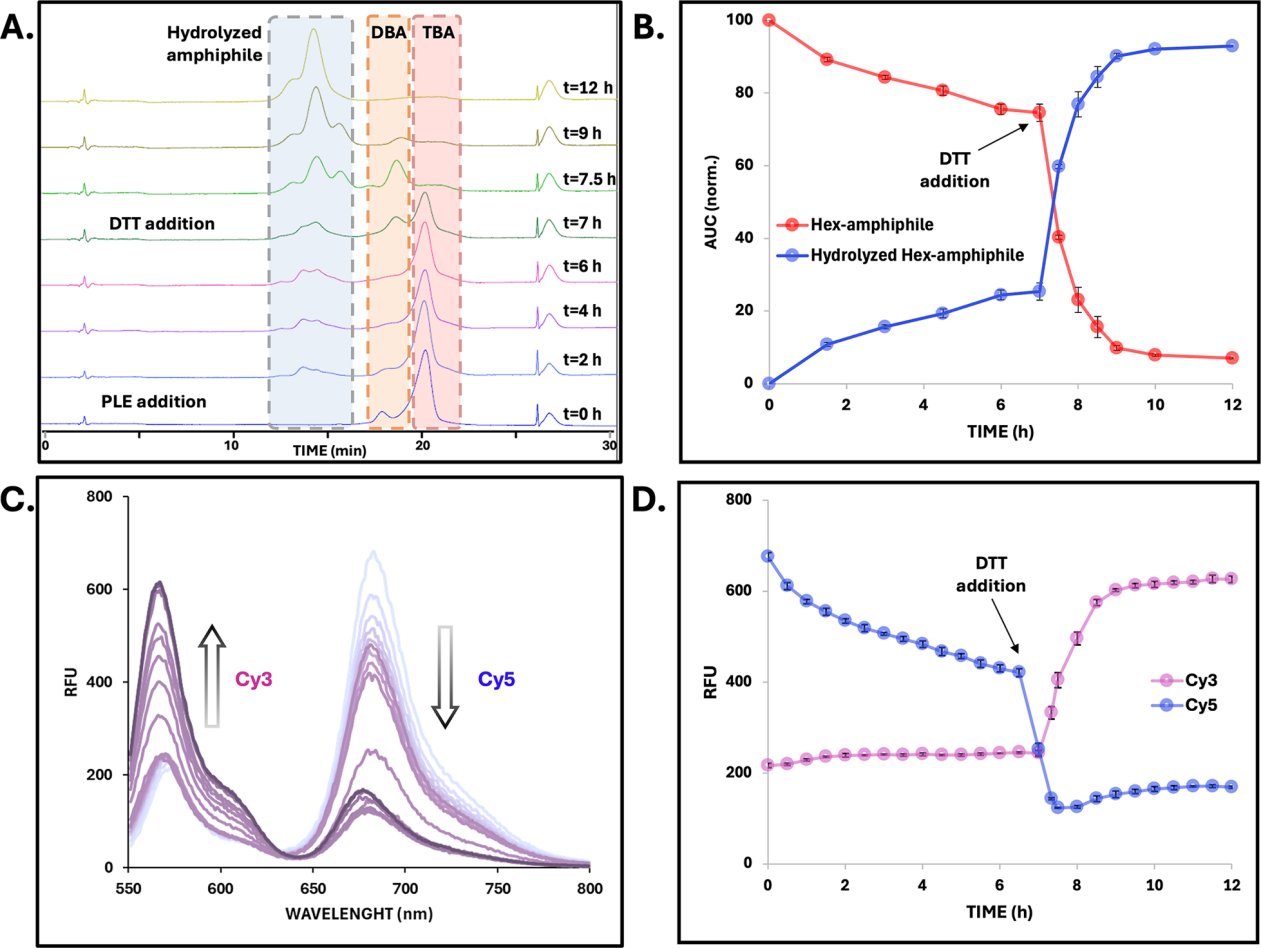
In situ architectural transition enhances enzymatic degradation and micelle disassembly. (A,B) Micellar degradation was monitored by HPLC to assess the molecular composition of the system over time: (A) overlay of HPLC chromatograms at selected time points (*t* = 0, 2, 4, 6, 7, 7.5, 9, and 12 h) and (B) analyzed enzymatic degradation profile. (C,D) Micellar disassembly was analyzed by fluorescence spectroscopy via FRET signal evolution. (C) Overlay of representative fluorescence emission spectra and (D) FRET signal decay over time, showing Cy3 intensity increase and Cy5 decrease upon disassembly. Experimental conditions: DTT was added at *t* = 7 h to induce in situ splitting of the TBA-Hex to DBA-Hex. [TBA] = 80 μM, [PLE] = 0.1 μM, [DTT] = 20 mM, λ_EX Cy3_ = 520 nm.

Next, we evaluated the enzymatic degradation kinetics across the full set of amphiphiles. Each formulation, TBA and DBA with Hex, Oct, and Dec end-groups, was incubated with PLE, and the percentage of the remaining parent amphiphile was quantified over time via HPLC (Figure 3A–C). To ensure that the degradation profile reflected structural variables and not splitting efficiency, the TBAs were pretreated with DTT, and full transition to DBAs was confirmed prior to enzyme addition (Figure S47). Further end-state analysis was conducted by DLS to confirm whether the polymers remained in the micellar state or disassembled due to their hydrolysis (Figure S49). As shown in Figure 3A, TBA-Hex showed slow degradation (∼25%) over the first 6 h and only 50% after 24 h, whereas DBA-Hex exhibited rapid enzymatic degradation, with over 90% hydrolyzed within less than 3 h. These observations are consistent with the expected higher stability of amphiphiles with triblock architecture and higher molecular weight.^61^ Increasing the hydrophobicity of the end-groups yielded a pronounced effect: DBA-Oct showed only 40% degradation after 24 h, and TBA-Oct remained largely intact with less than 10% hydrolysis over the same period of time (Figure 3B). Notably, both DBA-Dec and TBA-Dec exhibited negligible degradation (Figure 3C), with 7% and no degradation after 24 h, respectively, suggesting that their higher hydrophobicity imposes a threshold beyond which enzymatic degradation is suppressed, irrespective of amphiphile architecture. These results clearly demonstrate that small variations in end-group hydrophobicity can drastically affect enzymatic susceptibility and that architecture further modulates this effect. Across all pairs, the DBA versions degraded faster than their TBA counterparts, underscoring the influence of molecular weight and architecture. However, in the case of the decyl-based amphiphiles, the higher degree of hydrophobicity yielded micelles that remained highly stable and resistant to enzymatic degradation, regardless of architecture. These findings support a model in which hydrophobicity and architecture work in tandem to regulate enzymatic degradability.

To compare the enzymatic degradation to the dynamic properties of these micellar systems, we evaluated micelle–unimer exchange using a FRET-based mixing assay (Figures 1B and 3D–F). Each micellar formulation was divided into two populations, one containing 20% Cy3-labeled amphiphiles and 80% unlabeled amphiphiles, and the other micellar solution contained 20% Cy5-labeled and 80% unlabeled amphiphiles. Upon mixing of the two solutions in a 1:1 ratio, the development of the FRET signal was monitored by exciting the Cy3 (at 520 nm) and recording the emission intensities corresponding to Cy3 (570 nm) and Cy5 (670 nm) over time. The values were then normalized based on the *t* = 0 emission intensities. Since FRET occurs only when both dyes colocalize in the same micelle, its increase reflects unimer exchange between micelles, resulting in mixed-micelle formation (Figure 1B). As shown in Figure 3D, DBA-Hex exhibited the fastest exchange kinetics, with FRET signal reaching equilibrium within one hour. TBA-Hex showed a slower exchange rate, equilibrating after approximately three hours, in agreement with its expected higher kinetic stability. DBA-Oct exhibited a slightly slower exchange rate than TBA-Hex, while TBA-Oct showed minimal (∼5%) signal development (Figure 3E). The decyl-based systems exhibited the slowest exchange profile, with DBA-Dec showing minimal FRET signal evolution, even after extended incubation, and TBA-Dec having virtually no FRET over that time frame. These micelle–unimer exchange profiles mirror the enzymatic degradation data, with faster FRET evolution for DBA over TBA for each set and slower signal development as the amphiphiles’ hydrophobicity increases. These trends reinforce the hypothesis that faster micelle–unimer exchange facilitates enzymatic accessibility, as amphiphiles with faster mixing dynamics also exhibited more extensive degradation. This correlation between two independent data sets, degradation and chain exchange, provides compelling evidence for a dynamic mechanism of enzymatic reactivity. Furthermore, these results highlight the ability to control these two parameters by precise chemical modifications of the amphiphiles.

To analyze the relationship between micelle–unimer exchange and enzymatic degradation, we extracted the apparent rate constants from both data sets. For degradation, the natural logarithm of the normalized amphiphile concentration was plotted over time (Figure 4A), yielding a linear fit consistent with a first-order kinetic model: ln­([*A*]_*t*_/[*A*]_0_) = −*kt*. Similarly, monoexponential fits were applied to the FRET signal evolution to derive exchange rate constants (Figure 4B). While *k*_deg_ values extracted from HPLC reflect direct chemical cleavage, the *k*_ex_ values derived from FRET serve as comparative indicators of micelle–unimer exchange dynamics. Although DTT may slightly perturb the FRET in DBA micelles, its consistent presence across all DBA samples ensures valid intragroup comparison. Thus, despite potential environmental effects, this comparison captures the dynamic distinctions induced by the hydrophobicity and architecture. The calculated *k*_deg_ and *k*_ex_ values are summarized in Figure 4C.

Comparing TBAs and DBAs of identical hydrophobicity revealed that DBA variants consistently exhibited around one order of magnitude faster degradation and exchange rates, highlighting the impact of molecular weight and architecture on dynamic behavior. Interestingly, when looking at the *k*_deg_ and *k*_ex_ values for the TBA series, we noticed that there was also a difference between the two rates, with the enzymatic degradation being slower than the exchange rate. This can be attributed to the dependence of the enzymatic degradation mechanism on the exchange of monomers, so they can become accessible to the enzyme. As the enzyme cannot capture all unimers when outside of the micellar assemblies before they return into the micelles, the kinetics of the enzymatic degradation is thus slower.

**Figure 3:**
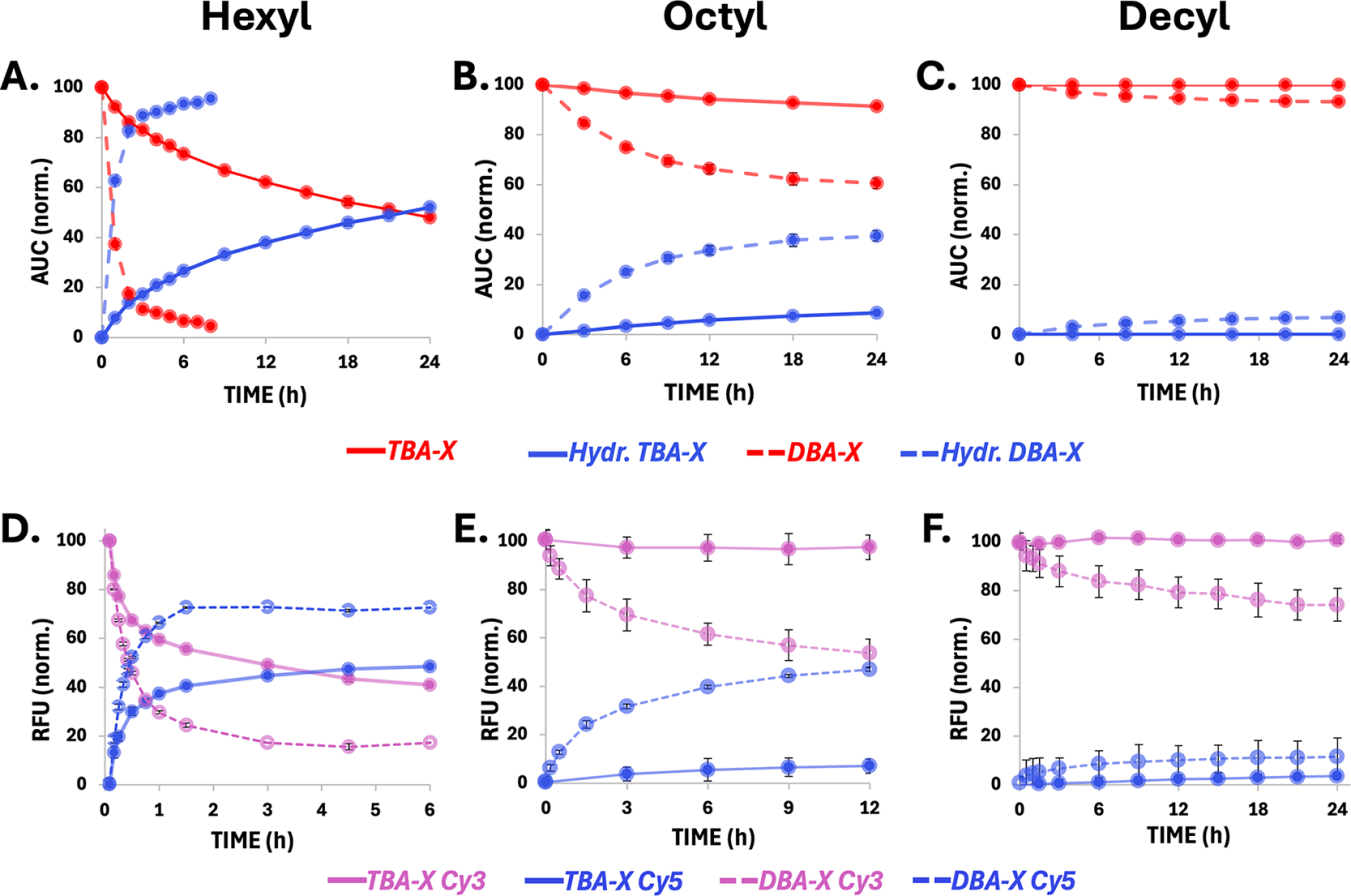
Enzymatic degradation and micelle–unimer exchange dynamics of TBA-X and DBA-X systems. (A–C) Enzymatic degradation profiles of (A) hexyl-, (B) octyl-, and (C) decyl-based micelles, monitored by HPLC. (D–F) FRET signal evolution (λ_EX Cy3_ = 520 nm), reflecting unimer exchange dynamics between separately labeled micellar populations for (D) hexyl-, (E) octyl-, and (F) decyl-based systems. Cy3 intensity decreases and Cy5 increases upon the spontaneous mixing of micellar populations. Experimental conditions: [TBA] = 80 μM; activating enzyme ([PLE] = 0.1 μM) was used for induced hydrolysis in (A-C); DBA-X systems were treated with DTT prior to all measurements ([DTT] = 20 mM).

To further explore the role of hydrophobicity, the extracted rate constants were plotted against the dendrons’ calculated cLogP values (Figure 4D,E). Although *c*Log*P* represents only the hydrophobicity of the dendron segment and not the complete amphiphile due to the hydrophilic PAA block, it provides a consistent quantitative parameter for comparing different hydrophobic blocks. For each type of end-group, both TBA and DBA were assigned the same cLogP values since they have the same dendritic block. However, as their architecture and overall hydrophobicity could not be quantified using the *c*-Log*P* parameter, the TBA and DBA systems were plotted separately.

Interestingly, across both architectures, we observed an exponential correlation between dendron hydrophobicity and both degradation and exchange rates (Figure 4D,E). This clear exponential trend underscores the significant and systematic influence of hydrophobic interactions on both dynamic behavior and consequently enzymatic responsiveness. Notably, *k*_deg_ trends exhibited similar slopes for the TBA and DBA series, suggesting that hydrophobicity exerts a comparable influence on enzymatic degradation independent of architecture. Similarly, *k*_ex_ values for TBA micelles mirrored *k*_deg_ trends, reinforcing the link between micelle–unimer dynamics and enzymatic accessibility. For DBA micelles, a slightly shallower *k*_ex_ slope was observed, possibly reflecting minor environmental effects of DTT rather than intrinsic differences in dynamic behavior. Nevertheless, the overall correlation between micellar dynamics and enzymatic degradation remains robust and consistent across all systems.

Importantly, the degradation and exchange rates paint consistent and meaningful structure–function relationships. Increasing the hydrophobicity of the end-groups resulted in more kinetically stable micelles that were less accessible to enzymatic cleavage, while the in situ architectural transition from TBA to DBA, accompanied by a reduction in molecular weight, consistently accelerated both exchange and degradation. Altogether, this study closes a key gap in our understanding of enzyme-responsive micelles and shows how precise structural modifications of architecture, molecular weight, and hydrophobicity can be used to program the stability and responsiveness of polymeric nanocarriers.

Furthermore, this deeper mechanistic understanding provides vital support for the design principle behind our recently reported polymeric micellar formulations composed of PEG-based diblock and triblock amphiphiles.^24,65,66^ These formulations undergo sequential mesophase transitions, from coassembled micelles to triblock-based hydrogels, due to the faster enzymatic degradation of the diblock amphiphiles. Ultimately, the hydrogel can also undergo further enzymatic degradation into fully dissolved polymers. We attributed the high enzymatic selectivity toward the diblock amphiphiles to their faster micelle–unimer exchange, which makes them more accessible to the activating enzyme compared to the triblock counterparts. The current study provides clear verification of this hypothesis and thus expands not only the fundamental understanding of the correlation between architecture, exchange rate, and degradability but also opens new directions for developing advanced polymeric formulations with complex response mechanisms.

**Figure 4:**
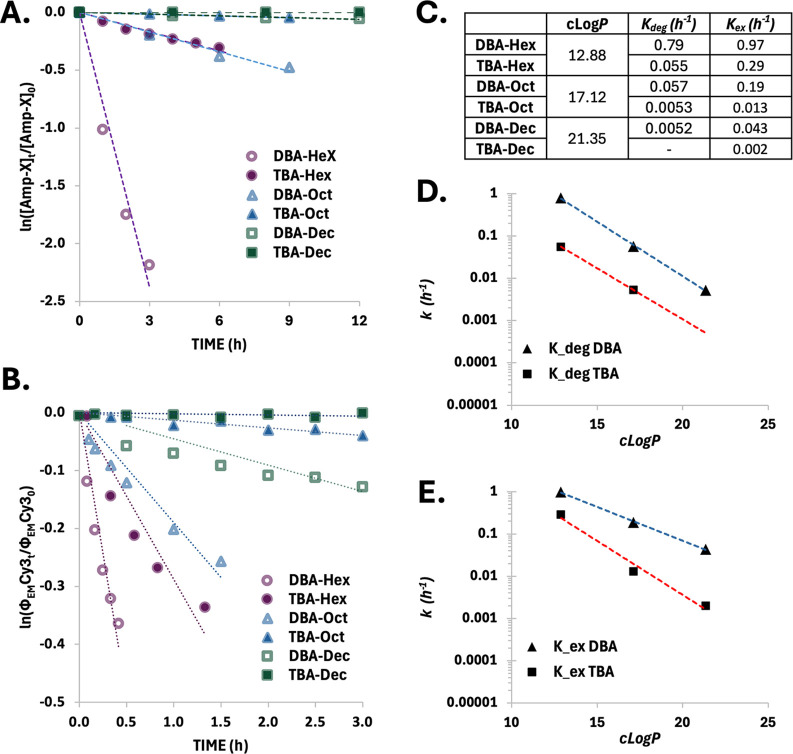
Kinetic evaluation of enzymatic degradation and micelle–unimer exchange across amphiphiles differing in architecture and hydrophobicity. (A,B) First-order fits of (A) enzymatic degradation (*k*_deg_) extracted from HPLC and (B) micelle–unimer exchange (*k*_ex_) derived from FRET signal evolution (circle, triangle, and square markers for hexyl, octyl, and decyl amphiphiles, respectively; full marker for TBAs; and empty markers for DBAs). (C) Summary of cLogP values calculated for the amphiphiles’ hydrophobic dendrons and corresponding rate constants. (D,E) Correlation plots of (D) *k*_deg_ and (E) *k*_ex_ versus *c* Log *P* values for the TBA (red) and DBA (blue) systems.

Moreover, combining the stimuli-induced change in architecture together with the ability to modularly tune the hydrophobicity of the dendritic blocks provides a strategy to overcome the stability–responsiveness balance, which holds back the potential applications of enzyme-responsive systems.

## Conclusions

To conclude, in this study, we set out to investigate the long-assumed correlation between enzymatic degradation and micelle–unimer exchange dynamics in polymeric micelles. Using a modular platform capable of in situ architectural transition from triblock to diblock amphiphiles along with systematic variation in the hydrophobicity of the end-groups, we constructed six micellar systems. FRET-based mixing experiments and HPLC-monitored hydrolysis assays under identical conditions were used to assess the relationship between micellar exchange and enzymatic degradation. Both data sets showed similar trends across all amphiphile designs: systems with faster micelle–unimer exchange consistently exhibited faster enzymatic degradation. This parallel behavior confirms that the two processes are closely linked, supporting the hypothesis that enzymatic cleavage occurs primarily via the unimeric species, hence providing direct mechanistic insight into how micelle dynamics dictate enzymatic responsiveness.

Altogether, this study demonstrates that both hydrophobicity and architecture independently and systematically can be applied to modulate micelle dynamics and enzymatic responsiveness. The excellent correlation between the exchange and enzymatic degradation rates across all systems firmly establishes micelle–unimer exchange not just as a key feature of dynamic assembly but as the mechanistic gatekeeper that dictates enzymatic accessibility and reactivity.

## Supplementary Material



## Notes

Detailed synthetic procedures, amphiphile and micelle characterization data, experimental protocols, and control experiment results (PDF)
