# Supplementary material for: Unimer Exchange as a Tool for Programming Enzymatic Degradation through Micellar Dynamics
Source: Biomacromolecules. 2025 Oct 10;26(11):7423–33. doi: 10.1021/acs.biomac.5c00955 (PMC12606637; doi:10.1021/acs.biomac.5c00955)
Supplement: Supplementary file 1 [file bm5c00955_si_001.pdf]

Unimer Exchange as a Tool for Programming Enzymatic Degradation through Micellar Dynamics

Shahar Tevet,[a,b,c] *Michal Brodsky*[a,b] *and Roey J. Amir**[a,b,c,d]

a. Department of Organic Chemistry, School of Chemistry, Faculty of Exact Sciences, Tel-Aviv University, Tel-Aviv, Israel
b. The Center for Nanoscience and Nanotechnology, Tel-Aviv University, Tel-Aviv, Israel
c. ADAMA Center for Novel Delivery Systems in Crop Protection, Tel-Aviv University, Tel-Aviv, Israel
d. The Center for Physics and Chemistry of Living Systems, Tel-Aviv University, Tel-Aviv, Israel

Supplementary Information

Table of Contents

Instrumentation and Materials **3**
Instrumentation 3
Materials 3
Synthesis and characterization of polymer-dendron amphiphiles **4**
Characterization of assembled micellar structures **48**
HPLC measurements 48
Critical micelles' concentration (CMC) 50
Dynamic light scattering 52
TEM imaging 53
Enzymatic degradation experiments and FRET mixing essays **54**
References **57**

Instrumentation and Materials

Instrumentation

^{1}H- and ^{13}C-NMR: spectra were recorded on Bruker Avance I and Avance III 400MHz (and 100MHz) spectrometers as indicated. Chemical shifts are reported in ppm and referenced to the solvent. **SEC:** All measurements were recorded on Viscotek GPCmax by Malvern using refractive index detector and PEG standards (purchased from Sigma-Aldrich) were used for calibration. **DLS:** All measurements were recorded on a Malvern Zetasizer NanoZS. **TEM:** Images were taken by a JEM-1400Plus TEM at 120kV. **Fluorescence spectra:** CMC measurements were recorded on a TECAN Infinite M200Pro device, enzymatic degradation experiment and FRET based mixing assays were recorded on an Agilent Technologies Cary Eclipse Fluorescence Spectrometer using quartz cuvettes. **HPLC:** All measurements were recorded on a Waters Alliance e2695 separations module equipped with a Waters 2998 photodiode array detector. All solvents were purchased from Bio-Lab Chemicals and were used as received. All solvents are HPLC grade.

Materials

3-mercaptopropionic acid (98%), 2,2-dimethoxy-2-phenylacetophenone (DMPA, 99%), 4-(Dimethylamino)pyridine (DMAP, 99%), Fmoc-L-Lys(Boc)-OH (98%), N-Hydroxysuccinimide (NHS, 99%), O-(Benzotriazol-1-yl)-N,N,N′,N′-tetramethyluronium hexafluorophosphate (HBTU), Copper(I) bromide (CuBr, 98%), N,N,N′,N′′,N′′-Pentamethyldiethylenetriamine (PMDETA, 99%), Bis[2-(2′-bromoisobutyryloxy)ethyl]disulfide, 1-hexanol, 1-octanol, 1-decanol, Porcine liver esterase (PLE) and SephadexR LH20 were purchased from Sigma-Aldrich. Propargyl bromide (80% in toluene), chlorotriphenylmethane (Trt-Cl, 98%), 4-nitrophenol (99%), triethylsilane (98%), N,N'-dicyclohexylcarbodiimide (DCC, 99%), propargyl amine (98%), tert-Butyl acrylate (tBA, 99%), triethylamine and anhydrous K_2CO_3 (99%) were purchased from Alfa Aesar. 3,5-dihydroxy benzoic acid was purchased from Apollo scientific. Cystamine hydrochloride (98%), Potassium hydroxide, Diisopropylethylamine (DIPEA) and sodium azide (NaN_3) were purchased from Merck. Silica Gel 60A, 0.040-0.063mm, sodium hydroxide, Anhydrous Na_2SO_4 (granular, 10-60mesh), piperidine (peptide synthesis), N,N-Dimethylformamide (DMF, peptide synthesis), Trifluoroacetic acid (TFA, HPLC grade) and all solvents were purchased from Bio-Lab and were used as received. Deuterated solvents for NMR were purchased from Cambridge Isotope Laboratories (CIL), Inc.

Synthesis and characterization of polymer-dendron amphiphiles

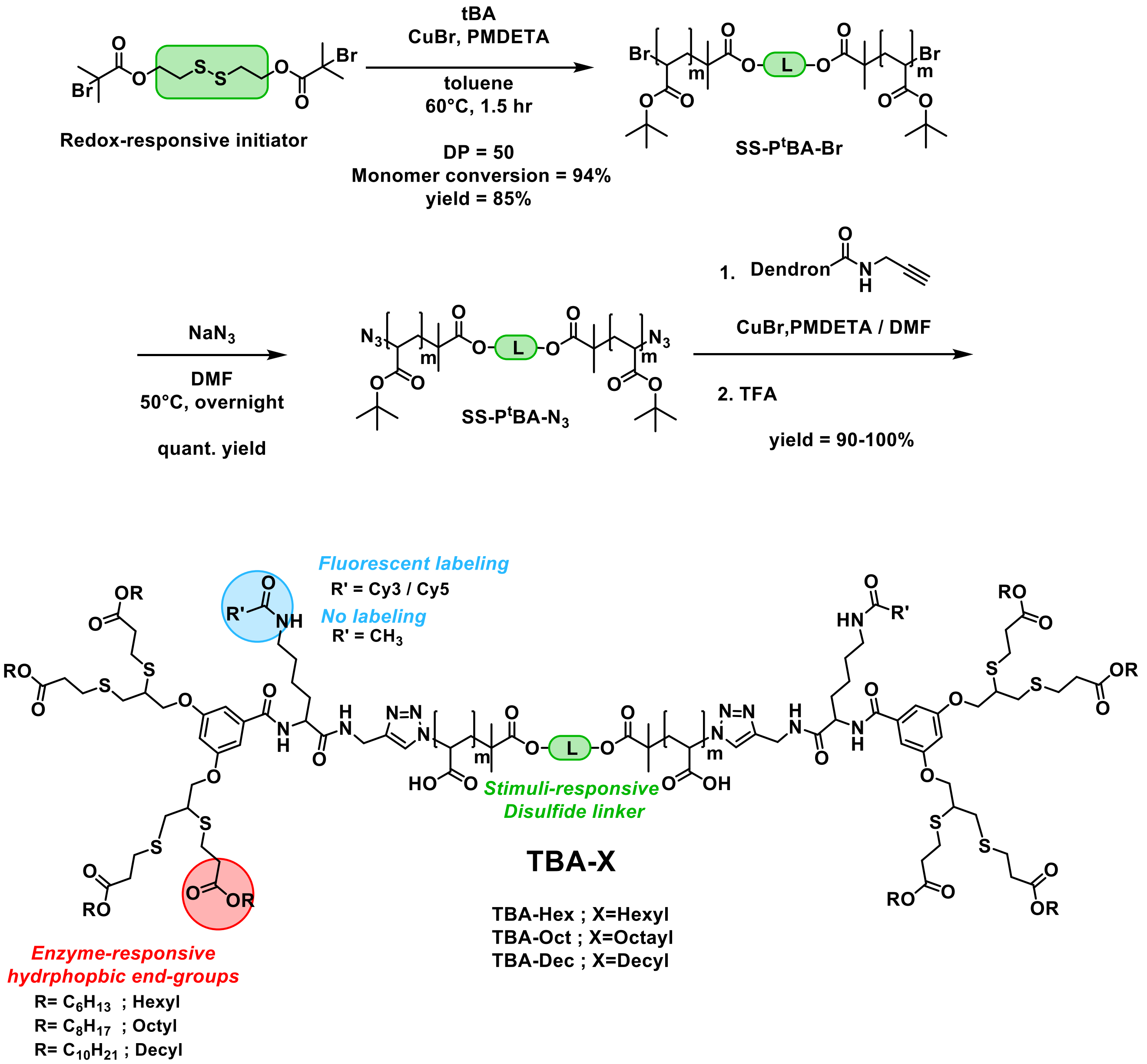

Figure S1: Synthetic route to splittable triblock amphiphiles SS-PAA-L(X)-D-4xAlkyl: The three amphiphile families differ in hydrophobicity, modulated by aliphatic end-groups (Hex, Oct, or Dec). Fluorophore labels (Cy3 and Cy5 and acetyl moiety were incorporated through lysine-based linker.

* Redox-responsive initiator Bis[2-(2′-bromoisobutyryloxy)ethyl]disulfide was purchased from Sigma-Aldrich and was used as received.

* SS-PtBA-Br and SS-PtBA-N_3 were synthesized as previously reported[1] and the spectroscopic characterization correlated well with these reports.

<u>Preparation of azide functionalized PtBA:</u>

<u>SS-PtBA-Br</u>

Redox-responsive initiator (502 mg, 1.11 mmol), PMDETA (470 µL, 2.22 mmol), tBA (8.5 mL, 58.02 mmol) and CuBr (318 mg, 2.22 mmol) were reacted according to the published procedure. The polymer was obtained as white solid in 85% yield (6.35 gr).

^{1}H NMR (400 MHz, Chloroform-*d*) δ 4.30 (br s, 4H, CH_2-C***H***$_2$-O), 4.10 (m, 2H, -C***H***-Br), 3.03 – 2.80 (t, J = 8.0 Hz 4H, CH_2-C***H***$_2$-S-), 2.44-2.06 (brs, 43H, PtBA backbone -C***H***-CO), 2.02-1.17 (m, 503H, PtBA backbone –C***H***$_2$-CH- + -O-C(C***H***$_3$)$_3$), 1.13 (s, 12H, -CO-C-(C***H***$_3$)$_2$-PtBA). DP= 50, Monomer conversion = 95%.

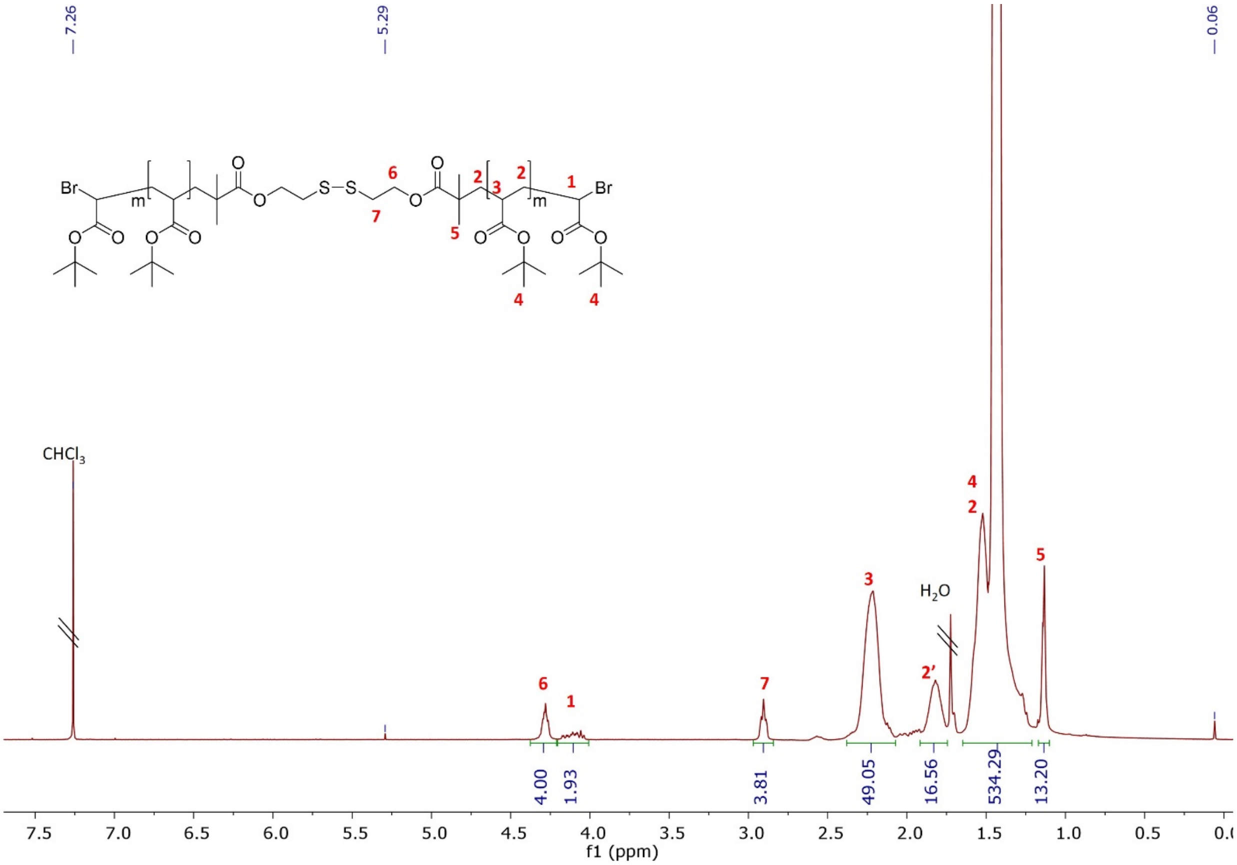

Figure S2: ^{1}H-NMR spectra of SS-PtBA-Br in $CDCl_3$.

SS-PtBA-N_3

SS-PtBA-Br (2.67 gr, 0.39 mmol) and NaN_3 (750 mg, 11.6 mmol) were reacted according to the published procedure. The product was obtained as white solid in quantitative yield (2.62 gr).

^{1}H NMR (400 MHz, Chloroform-*d*) δ 4.29 (br s, 4H, CH_2-C***H*₂**-O), 3.83 – 3.57 (m, 2H, -C***H***-N_3), 2.91 (br s, 4H, CH_2-C***H*₂**-S-), 2.40-2.12 (brs, 38H, PtBA backbone -C***H***-CO), 2.03-1.21 (m, 486H, PtBA backbone –C***H***$_2$-CH- + -O-C(C***H***$_3$)$_3$), 1.14 (s, 12H, -CO-C-(C***H***$_3$)$_2$-PtBA).

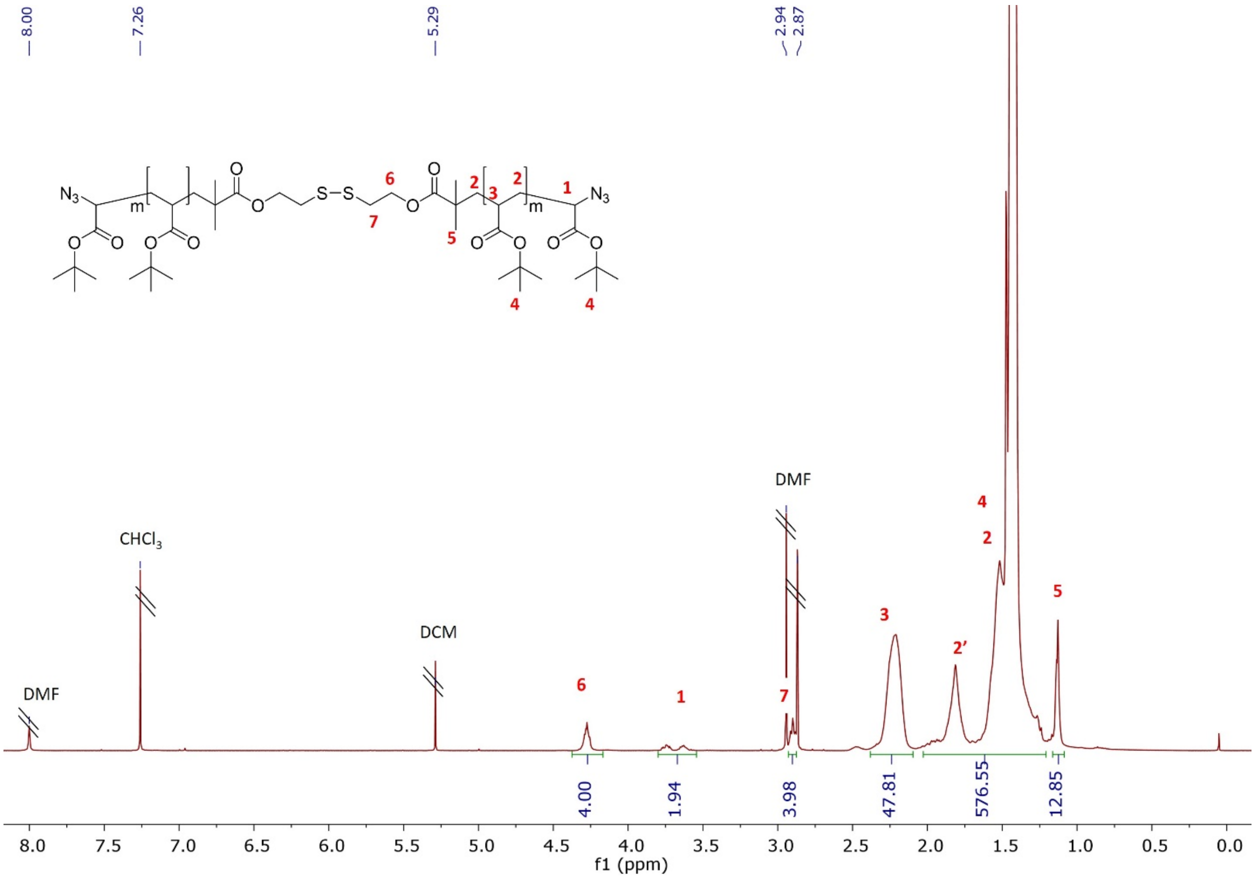

Figure S3: ^{1}H-NMR spectra of SS-PtBA-N_3 in $CDCl_3$.

Preparation of esterase-responsive labelled dendrons:

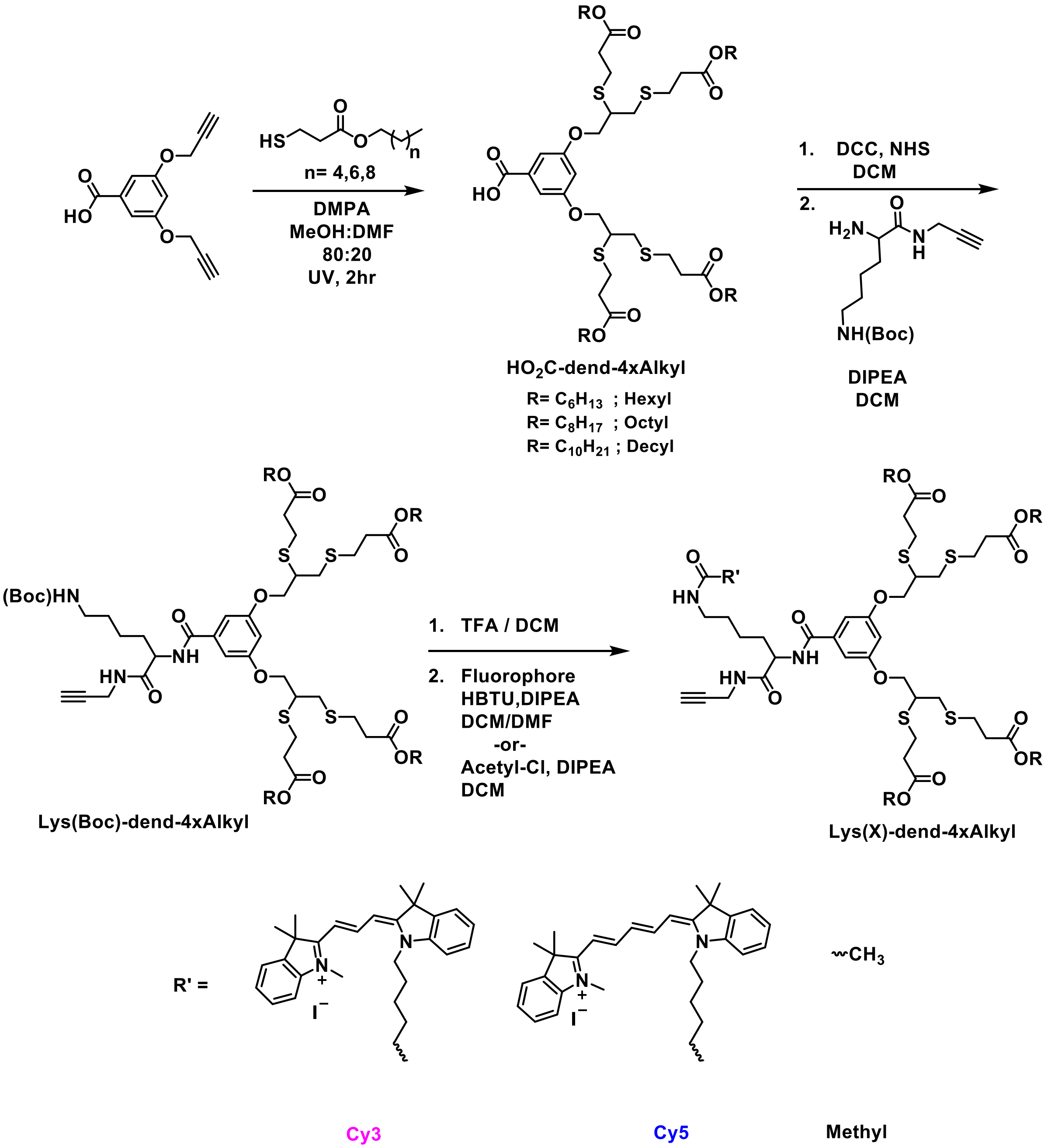

Figure S4: Synthetic of fluorescently labeled enzyme-responsive dendrons with increasing hydrophobicity and different labeling moieties.

* 3,5-bis(propargyloxy) benzoic acid[2] and NH_2-Lys(Boc)-propargyl amide[3] were synthesized as previously reported and the spectroscopic characterization correlated well with these reports.

Synthesis of thiolated enzyme-responsive hydrophobic end-group

General procedure for Alkyl 3-mercaptopropanoate

3-(tritylthio)propanoic acid[4] and the relevant alcohols were suspended in DCM (10mL for 1gr). Then, DCC (1.1eq) and DMAP (0.3eq) were added, and the reaction was stirred overnight at room temperature. The reaction was filtered, and TFA was added, followed by triethyl silane (1.3eq). The reaction was stirred at room temperature for 10 minutes and evaporated to dryness. The product was purified by silica column (35:65 Hex:DCM v/v) (TLC plates were stained with $KMnO_4$). Products were obtained as yellowish oils.

Hexyl 3-mercaptopropanoate

3-(tritylthio)propanoic acid (3gr, 9.4mmol), 1-hexanol (1.2mL, 0.96gr, 9.4mmol), DCC (1.9gr, 9.4mmol) and DMAP (0.31gr, 2.5mmol) were reacted according to the general procedure. Then, 8mL TFA was added, followed by triethyl silane (1.8mL, 1.3gr, 11.1mmol). The product was obtained as a yellowish liquid in 60% yield (0.98gr). The ^{1}H-NMR characterization correlated well with the previous report by the group.[5]

Octyl 3-mercaptopropanoate

3-(tritylthio)propanoic acid (3gr, 9.4 mmol), 1-octanol (1.5mL, 1.2gr, 9.4mmol), DCC (1.9gr, 9.4mmol), and DMAP (0.31gr, 2.5mmol) were reacted according to the general procedure. Then, 8 mL TFA was added, followed by triethyl silane (1.8mL, 1.3gr, 11.1mmol). The product was obtained as a yellowish liquid in 70% yield (1.30 gr).

^{1}H-NMR (400MHz, $CDCl_3$): δ 4.10 (t, J = 6.8 Hz, 2H, -COO-C**H$_2$**-), 2.80-2.75 (m, 2H, HS-C**H$_2$**-), 2.64 (td, J = 6.8, 0.8 Hz, 2H, -C**H$_2$**-COO-), 1.66-1.59 (m, 3H, -COO-CH_2-C**H$_2$**- + **H**S-), 1.38-1.22 (m, 10H), 0.88 (t, J = 6.7 Hz, 3H). ^{13}C-NMR ($CDCl_3$): δ 171.58, 64.80, 38.43, 31.57, 29.07, 28.49, 25.79, 22.52, 19.69, 13.97.

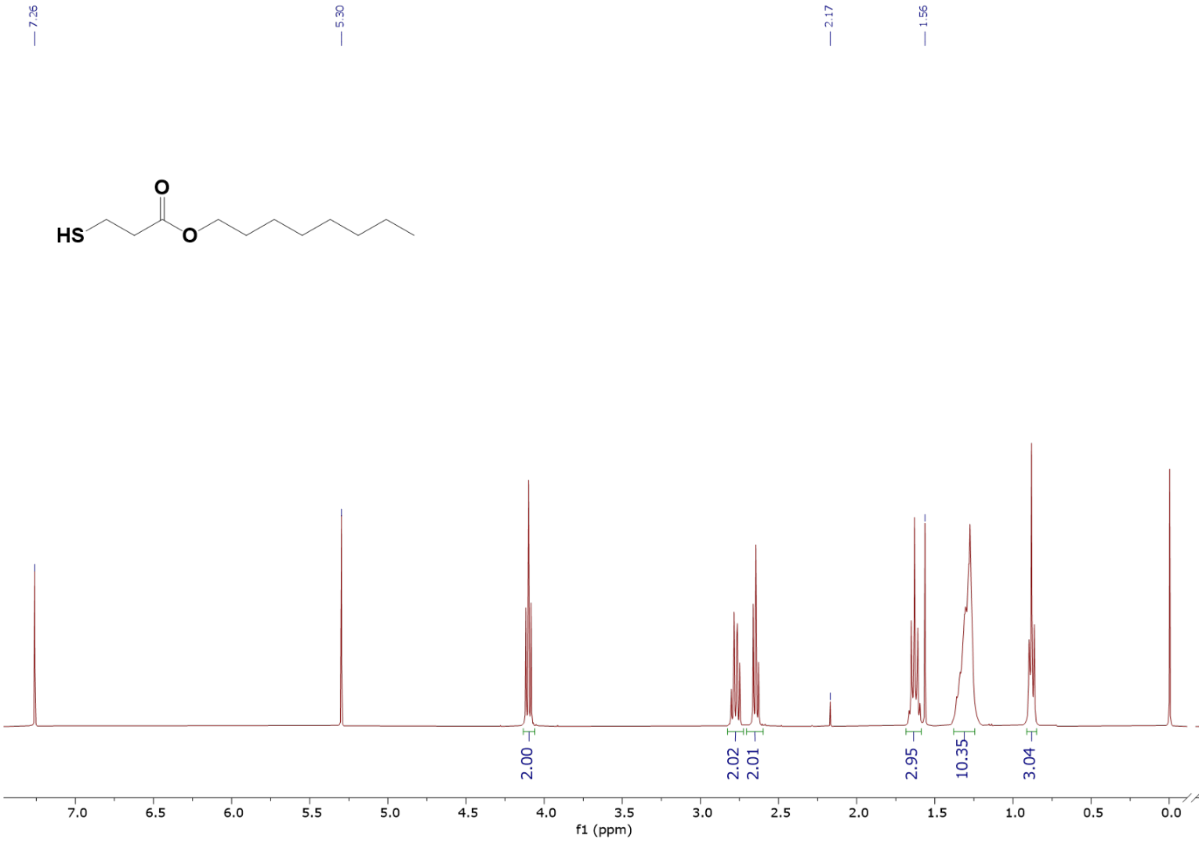

Figure S5: ^{1}H-NMR spectra of Octyl 3-mercaptopropanoate in $CDCl_3$.

<u>Decyl 3-mercaptopropanoate</u>

3-(tritylthio)propanoic acid (3gr, 9.4mmol), 1-decanol (1.8mL, 1.5gr, 9.4mmol) DCC (1.9gr, 9.4mmol) and DMAP (0.31gr, 2.5mmol) were reacted according to the general procedure. Then, 8 mL TFA was added, followed by triethyl silane (1.8mL, 1.3gr, 11.1mmol). The product was obtained as a yellowish liquid in 80% yield (1.69gr).

^{1}H-NMR (400MHz, $CDCl_3$): δ 4.10 (m, 2H, -COO-C**H_2**-), 2.80-2.75 (m, 2H, HS-C**H_2**-), 2.64 (m, 2H, -C**H_2**-COO-), 1.67-1.59 (m, 3H, -COO-CH_2-C**H_2**- + **H**S-), 1.38-1.22 (m, 10H), 0.88 (t, J = 6.7 Hz, 3H). ^{13}C-NMR ($CDCl_3$): δ 171.83, 65.07, 38.71, 32.03, 29.66, 29.44, 29.38, 28.76, 26.06, 22.82, 19.96, 14.24.

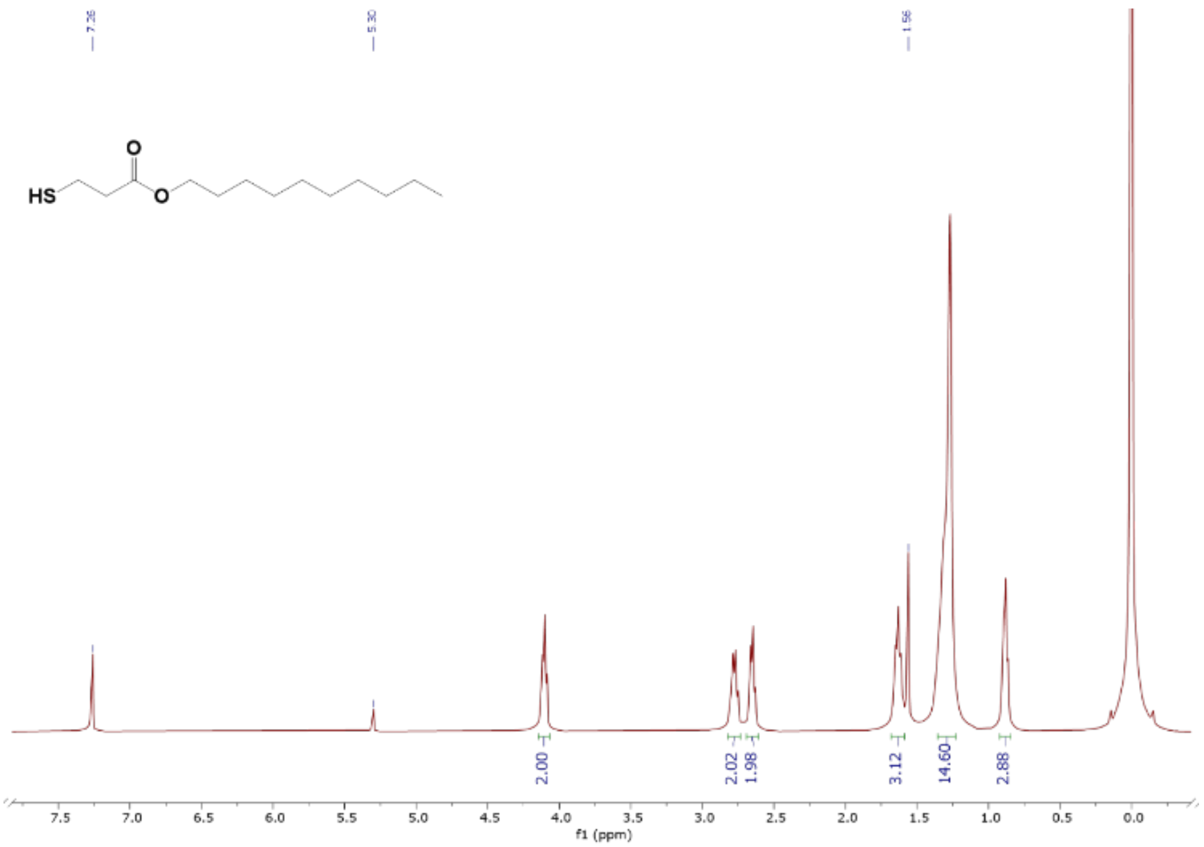

Figure S6: ^{1}H-NMR spectra of Decyl 3-mercaptopropanoate in $CDCl_3$.

General procedure for HO_2C-dend-4xAlkyl compounds

3,5-bis(propargyloxy) benzoic acid, the relevant thiol (8eq) and DMPA (0.4eq) were dissolved in a MeOH:DMF (80:20v/v) solvent mixture. The reaction was purged with nitrogen for 15 minutes, sonicated for 15 minutes, and stirred under UV light for 2 hours. The solvents were evaporated until dryness. The product was purified by silica column (50:50 EtOAc:DCM). The products were obtained as yellowish oils.

HO_2C-dend-4xHex

HO_2C-BU (0.14gr, 0.62mmol), Hexyl 3-mercaptopropanoate (0.94gr, 4.92mmol), and DMPA (60mg, 0.25mmol) were dissolved in 2.5mL of MeOH:DMF (80:20v/v) and reacted according to the general procedure. The product was obtained as yellowish oil in 70% yield (0.43gr). The ^{1}H-NMR characterization correlated well with the previous report by the group.[3]

HO_2C-dend-4xOct

HO_2C-BU (0.13gr, 0.57mmol), Octyl 3-mercaptopropanoate(1gr, 4.58mmol), and DMPA (58mg, 0.22mmol) were dissolved in 4mL of MeOH:DMF (80:20v/v) and reacted according to the general procedure. The product was obtained as yellowish oil in 88% yield (0.55gr).

^{1}H-NMR (400MHz, $CDCl_3$): δ 7.25 (d, J = 2.2 Hz, 2H), 6.72 (t, J = 2.3 Hz, 1H), 4.28-4.15 (m, 4H, Ar-OC**H**$_2$-), 4.14-4.02 (m, 8H, -COO-C**H**$_2$-) , 3.19 (quin, J = 6.1 Hz, 2H, -C**H**-S-), 3.02-2.80 (m, 12H, -C**H**$_2$-S-), 2.68-2.58 (m, 8H, -C**H**$_2$-COO-), 1.54-1.66 (m, 8H, -COO-CH2-C**H**$_2$-), 1.37-1.15 (m, 40H), 0.88 (t, J = 6.8 Hz, 12H)

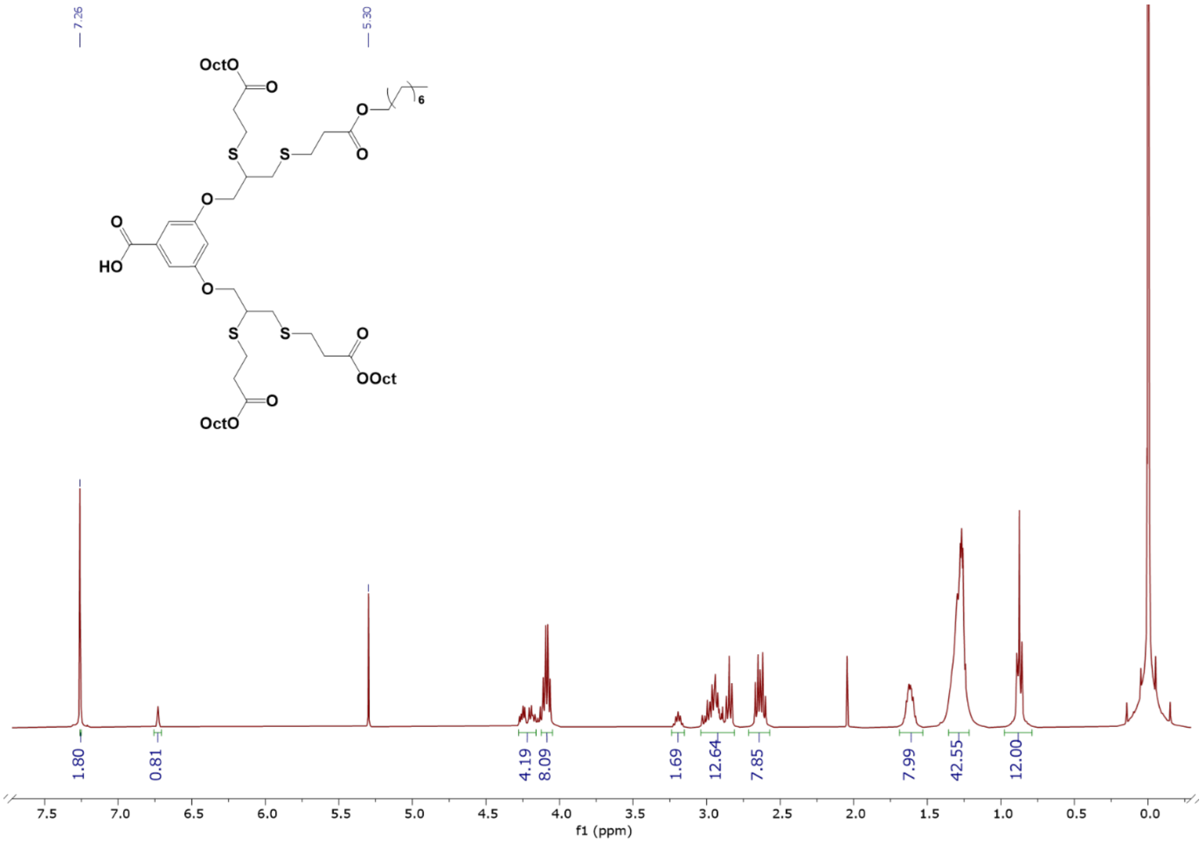

Figure S7: ^{1}H-NMR spectra of HO_2C-dend-4xOct in $CDCl_3$.

<u>HO_2C-dend-4xDec</u>

HO_2C-BU (0.20gr, 0.86mmol), Decyl 3-mercaptopropanoate (1.70gr, 6.88mmol), and DMPA (88mg, 0.34mmol) were dissolved in 4mL of MeOH: DMF (80:20v/v) and reacted according to the general procedure. The product was obtained as yellowish oil in 65% yield (0.70gr).

^{1}H-NMR (400MHz, $CDCl_3$): δ 7.25 (d, J = 2.2 Hz, 2H), 6.70 (t, J = 2.3 Hz, 1H), 4.25-4.15 (m, 4H, Ar-OC**H**$_2$-), 4.14-4.06 (m, 8H, -COO-C**H**$_2$-) , 3.19 (quin, J = 6.2 Hz, 2H, -C**H**-S-), 3.02-2.80 (m, 12H, -C**H**$_2$-S-), 2.68-2.58 (m, 8H, -C**H**$_2$-COO-), 1.54-1.66 (m, 8H, -COO-CH2-C**H**$_2$-), 1.37-1.15 (m, 56H), 0.88 (t, J = 6.8 Hz, 12H)

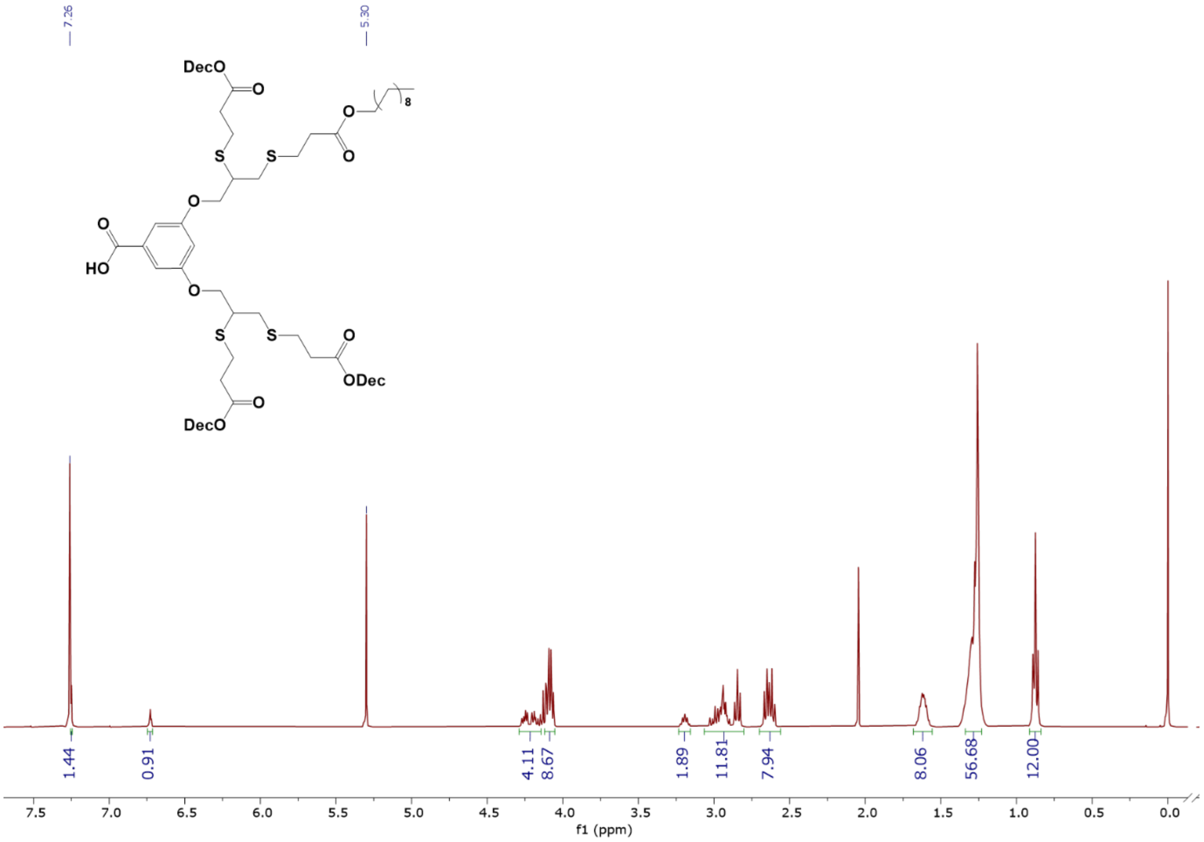

Figure S8: ^{1}H-NMR spectra of HO_2C-dend-4xDec in $CDCl_3$.

<u>General procedure for Lys(Boc)-dend-4xAlkyl compounds</u>

HO_2C-dend-4xAlkyl and NHS (1.1eq) were suspended in DCM, then DCC (1.1eq) was added. The reaction was stirred at room temperature for 1 hour (HPLC and TLC confirmed full activation to NHS ester), filtered, and DIPEA (9eq) was added, followed by NH_2-Lys(Boc)-propargyl amide (1.3eq), and the reaction was stirred overnight at RT. The reaction was diluted with DCM and washed with NH_4Cl. Solvents were evaporated, and the product was purified by a silica column (50:50 EtOAc:DCM). The products were obtained as a yellow-dense oil.

<u>Lys(Boc)-dend-4xHex</u>

HO_2C-dend-4xHex (0.30gr, 0.30mmol), NHS (34mg, 1.47mmol), DCC (0.30gr, 1.47mmol), DIPEA (0.35gr, 2.72mmol) and NH_2-Lys(Boc)-propargyl amide (0.11gr, 0.39mmol) were reacted according to the general procedure in 7.5mL DCM. The product was obtained as a yellow-dense oil in 70% yield (0.26gr). The ^{1}H-NMR characterization correlated well with the previous report by the group.[5]

Lys(Boc)-dend-4xOct

HO_2C-dend-4xOct (1.27gr, 1.15mmol), NHS (0.13gr, 1.27mmol), DCC (0.26gr, 1.27mmol), DIPEA (1.34gr, 10.40mmol) and NH_2-Lys(Boc)-propargyl amide (0.42gr, 1.50mmol) were reacted according to the general procedure in 30mL DCM. The product was obtained as a yellow-dense oil in 53% yield (0.77gr).

^{1}H-NMR (400MHz, $CDCl_3$): δ 7.00 (d, J = 2.3 Hz, 3H, Ar + -N**H**–CH_α-), 6.66 (brs, 1H, CH≡C-CH_2-N**H**-CO-), 6.62 (t, J = 2.3 Hz, 1H), 4.57 (q, J = 7.5 Hz, 1H, -NH-C**H**$_\alpha$-CO-NH-) 4.28-4.00 (m, 14H, Ar-O-C**H₂**- + CO-O-C**H₂**- + -NH-C**H₂**-C≡CH), 3.24-2.76 (m, 16H, -C**H₂**-S- + -C**H**-S- + -C**H₂**-NH(Boc)), 2.70-2.57 (m, 8H, -C**H₂**-CO-O-), 2.22 (t, J = 2.5 Hz, 1H, -NH-CH_2-C≡C**H**), 2.03-1.97 (m, 2H, -CH_α-C**H₂**-CH_2-), 1.67-1.59 (m, 8H, -CO-OCH_2-C**H₂**-), 1.42 (s, 9H, NH(**Boc**)), 1.34-1.21 (m, 44H, -CH_α-CH_2-C**H₂**-C**H₂**- + (C**H₂**)$_5$-CH_3), 0.87 (t, J = 6.8 Hz, 12H, -$(CH_2)_6$-C**H₃**).

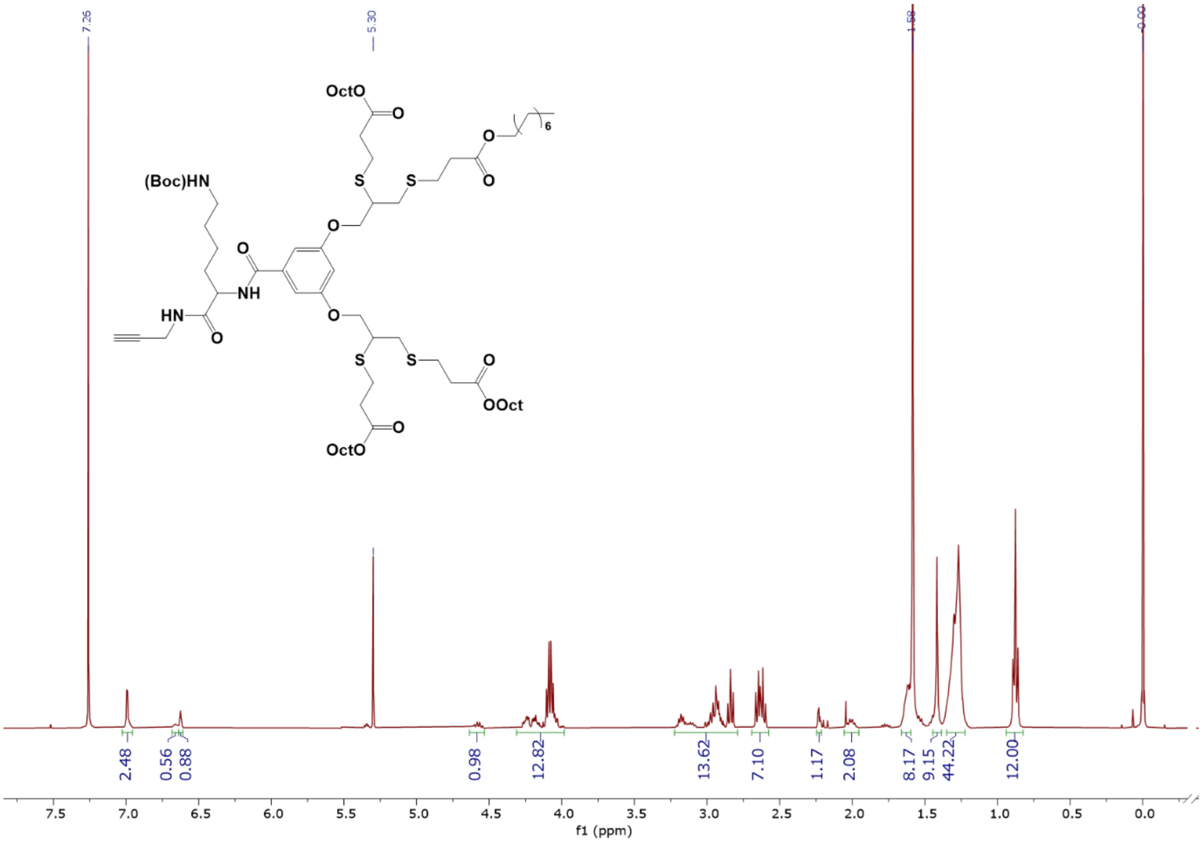

Figure S9: ^{1}H-NMR spectra of Lys(Boc)-dend-4xOct in $CDCl_3$.

Lys(Boc)-dend-4xDec

HO_2C-dend-4xDec (0.40gr, 0.328mmol) and NHS (0.041gr, 0.394mmol), DCC (81mg, 0.40mmol), DIPEA (0.40gr, 2.96mmol) and NH_2-Lys(Boc)-propargyl amide (0.12gr, 0.42mmol) were reacted according to the general procedure in 10mL DCM. The product was obtained as a yellow-dense oil with an 82% yield (0.403gr).

^{1}H-NMR (400MHz, $CDCl_3$): δ 7.00 (d, J = 2.3 Hz, 2H), 6.93 (brs, 1H, -N**H**–CH_α-), 6.66 (t, J = 2.3 Hz, 1H), 6.52 (brs, 1H, CH≡C-CH_2-N**H**-CO-), 4.57 (q, J = 7.4 Hz, 1H, -NH–C**H**$_\alpha$-CO-NH-) 4.28-4.00 (m, 14H, Ar-O-C**H₂**- + CO-

O-C$\mathbf{H_2}$- + -NH-C$\mathbf{H_2}$-C≡CH), 3.24-2.76 (m, 16H, -C$\mathbf{H_2}$-S- + -C**H**-S- + -C$\mathbf{H_2}$-NH(Boc)), 2.70-2.54 (m, 8H, -C$\mathbf{H_2}$-CO-O-), 2.22 (t, J = 2.6 Hz, 1H, -NH-CH$_2$-C≡C**H**), 1.98-1.88 (m, 2H, -CH$_\alpha$-C$\mathbf{H_2}$-CH$_2$-), 1.67-1.59 (m, 8H, -CO-OCH$_2$-C$\mathbf{H_2}$-), 1.42 (s, 9H, NH(**Boc**)), 1.35-1.19 (m, 60H, -CH$_\alpha$-CH$_2$-C$\mathbf{H_2}$-C$\mathbf{H_2}$- + (C$\mathbf{H_2}$)$_7$-CH$_3$), 0.87 (t, J = 6.4 Hz, 12H, -(CH$_2$)$_6$-C$\mathbf{H_3}$).

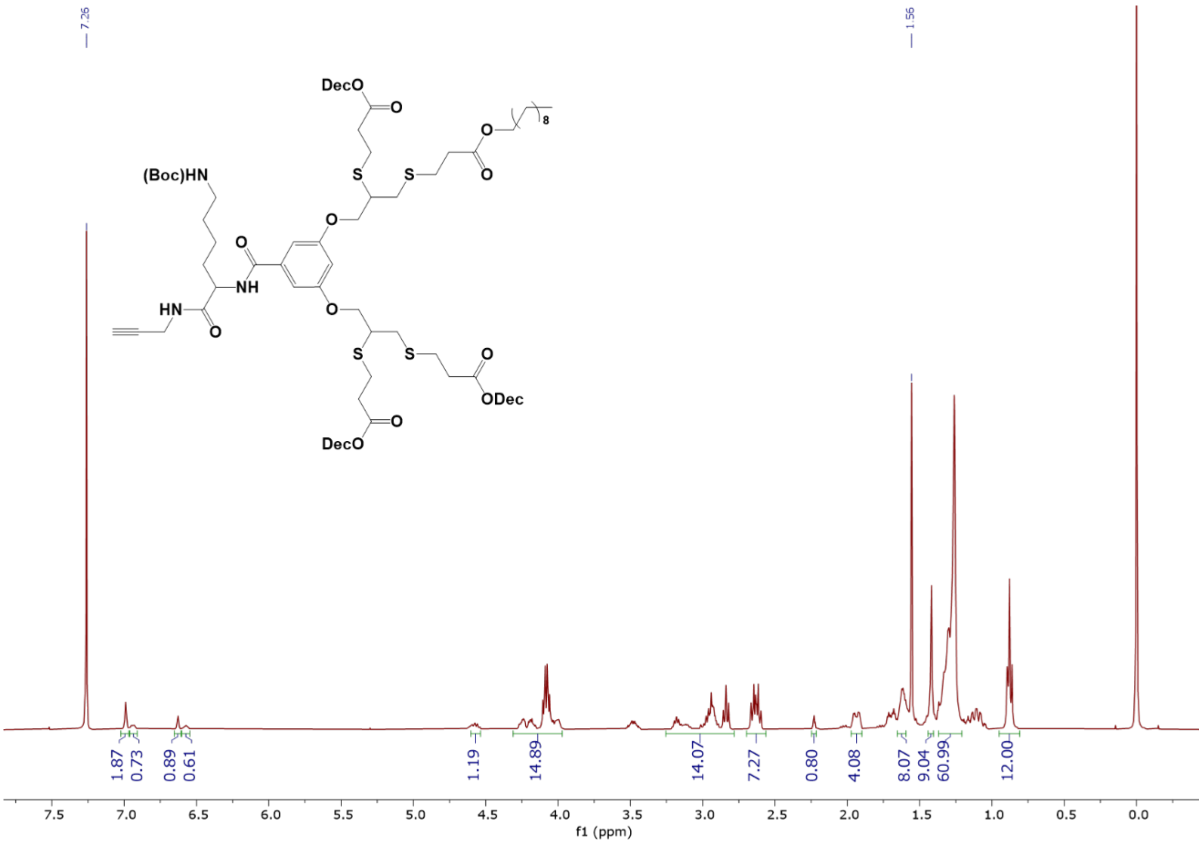

Figure S10: ^{1}H-NMR spectra of Lys(Boc)-dend-4xDec in CDCl$_3$.

<u>Synthesis of Lys(X)-dend-4xAlkyl</u>

General procedure for labelling Lys(Boc)-dend-4X compounds with Cy3, Cy5 and methyl

Boc deprotection: Lys(Boc)-dend-4xAlkyl were dissolved in DCM:TFA (1:1v/v), and the mixture was allowed to stir for 15 minutes. HPLC confirmed complete Boc deprotection, and then the reaction mixture was evaporated and placed on a high vacuum for 30 minutes. Then, compound was dissolved in minimal amount of DCN, and DIPEA (4eq) was added to the reaction mixture.

Cy$_x$ labeling: Cy3/Cy5 (1.1eq), HBTU (1.1eq), and DIPEA (4eq) were dissolved in DCM:DMF (1:1 v/v) in a separate vial. The solution was stirred for 5 minutes and then added to the reaction mixture with the dendron. The reaction was stirred for 1.5 hours at RT, and HPLC confirmed the completion of the reaction. Solvents were removed under reduced pressure, and the products were purified by a MeOH-based LH20 SEC column. Products were obtained as a dark blue or pink dense oils, for Cy5 and Cy3 labelling, accordingly.

Acetyl labeling: Acetyl chloride (1.1eq) was added, and the mixture was stirred for 2 hours RT. HPLC confirmed the completion of the reaction. The reaction was diluted with DCM, washed with NH_4Cl, and then with brine. Solvents were removed under reduced pressure, and the product was purified by a MeOH-based LH20 SEC column. Products were obtained as colorless oils.

Lys(Cy5)-dend-4xHex

Lys(Boc)-dend-4xHex (75mg, 0.059mmol) was dissolved in DCM:TFA (1:1v/v) (1mL). Then, it was reacted according to the general procedure with Cy5 (40mg, 0.065mmol), HBTU (24mg, 0.065mmol), and DIPEA (80mg, 0.47mmol) in DCM:DMF (1:1 v/v) (3mL). Product was obtained as a dark blue dense oil in quantitative yield (100mg).

^{1}H-NMR (400MHz, $CDCl_3$): see following spectrum and assignments.

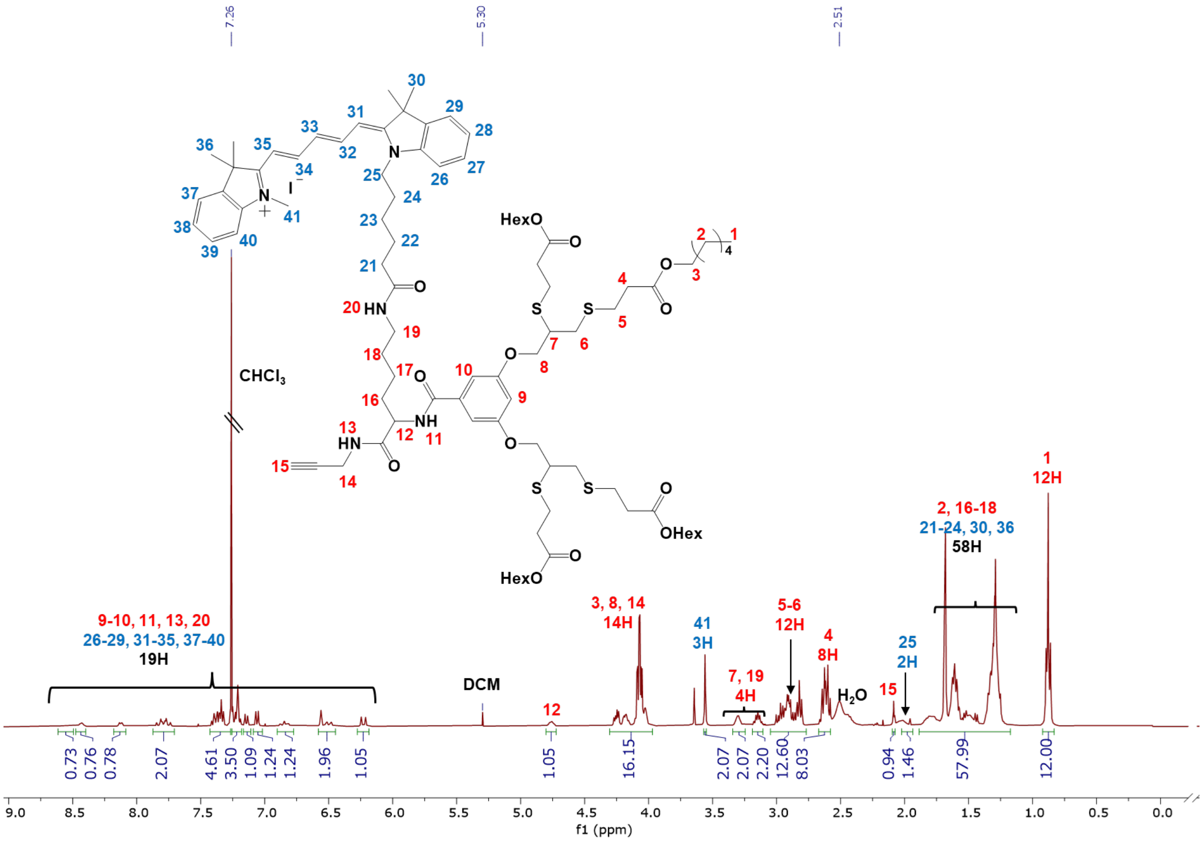

Figure S11: ^{1}H-NMR spectra of Lys(Cy5)-dend-4xHex in $CDCl_3$.

Lys(Cy5)-dend-4xOct

Lys(Boc)-dend-4xOct (50mg, 0.036mmol) was dissolved in DCM:TFA (1:1v/v) (0.6mL). Then, it was reacted according to the general procedure with Cy5 (50mg, 0.040mmol), HBTU (16.3mg, 0.040mmol), and DIPEA (40mg, 0.28mmol) in DCM:DMF (1:1 v/v) (2mL). Product was obtained as a dark blue dense oil in quantitative yield (66mg).

^{1}H-NMR (400MHz, $CDCl_3$): see following spectrum and assignments.

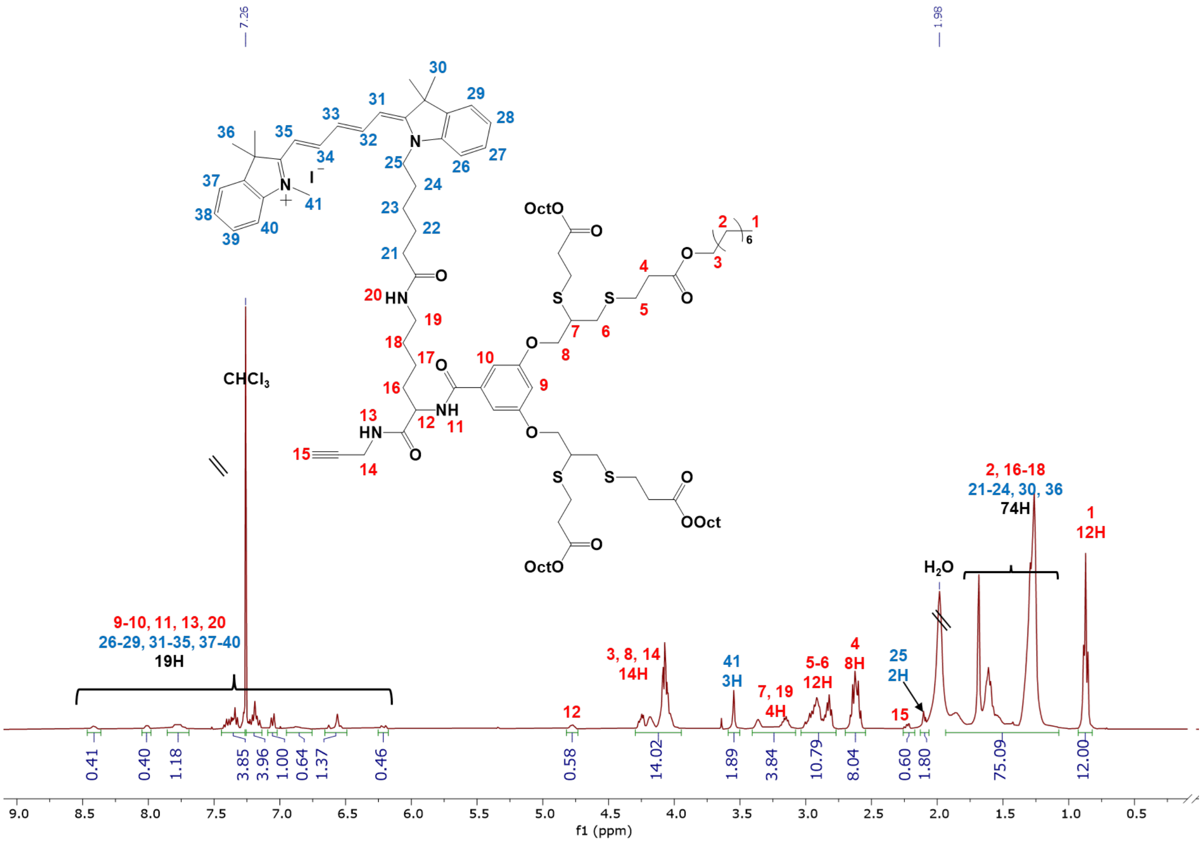

Figure S12: ^{1}H-NMR spectra of Lys(Cy5)-dend-4xOct in $CDCl_3$.

Lys(Cy5)-dend-4xDec

Lys(Boc)-dend-4xDec (72mg, 0.048mmol) was dissolved in DCM:TFA (1:1v/v) (1mL). Then, it was reacted according to the general procedure with Cy5 (36mg, 0.060mmol), HBTU (22.3mg, 0.060mmol), and DIPEA (80mg, 0.38mmol) in DCM:DMF (1:1 v/v) (3mL). Product was obtained as a dark blue dense oil in quantitative yield (90mg).

^{1}H-NMR (400MHz, $CDCl_3$): see following spectrum and assignments.

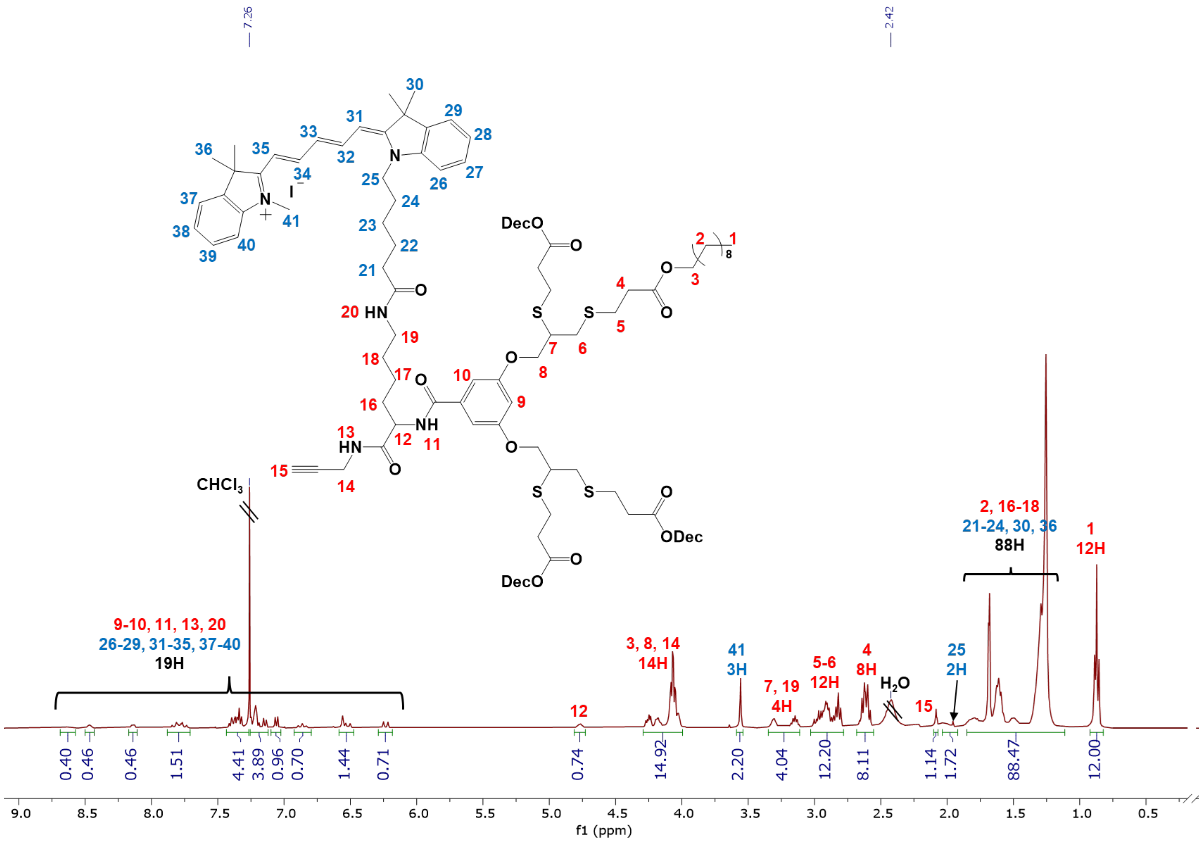

Figure S13: ^{1}H-NMR spectra of Lys(Cy5)-dend-4xDec in $CDCl_3$.

Lys(Acetyl)-dend-4xHex

Lys(Boc)-dend-4xHex (65mg, 0.05mmol) was dissolved in DCM:TFA (1:1v/v) (1mL). Then, it was reacted according to the general procedure with DIPEA (25mg, 0.20mmol) and Acetyl chloride (4.3mg, 0.056mmol). Product was obtained as a colorless oil in 84% yield (53mg).

^{1}H-NMR (400MHz, $CDCl_3$): δ 7.22 (d, J = 7.7 Hz, 1H, -N**H**-CH_α-), 7.09-7.00 (m, 3H, Ar-**H +** , -N**H**-CO-CH_3), 6.62 (t, J = 2.2 Hz, 1H, Ar-**H**), 5.90 (t, J = 6.0 Hz, 1H, -N**H**-CH_2-C≡CH), 4.71-4.55 (m, 1H, -NH–C**H**$_\alpha$-CO-NH-), 4.31-3.89 (m, 14H, Ar-O-C**H$_2$**- + CO-O-C**H$_2$**- + -NH-C**H$_2$**-C≡CH), 3.34-3.07 (m, 4H, -C**H**-S + -C**H$_2$**-NH-CO-CH_3), 3.00-2.80 (m, 12H, -C**H$_2$**-S-), 2.69-2.60 (m, 8H, C**H$_2$**-CO-O-), 2.22 (t, J = 2.5 Hz, 1H, -NH-CH_2-C≡C**H**), 1.93 (s, 3H, -NH-CO-C**H$_3$**), 1.67-1.16 (m, 38H, -CH_α-C**H$_2$**-C**H$_2$**-C**H$_2$**- + (C**H$_2$**)$_4$-CH_3), 0.94-0.79 (t, J= 6.5, 12H, -$(CH_2)_4$-C**H$_3$**).

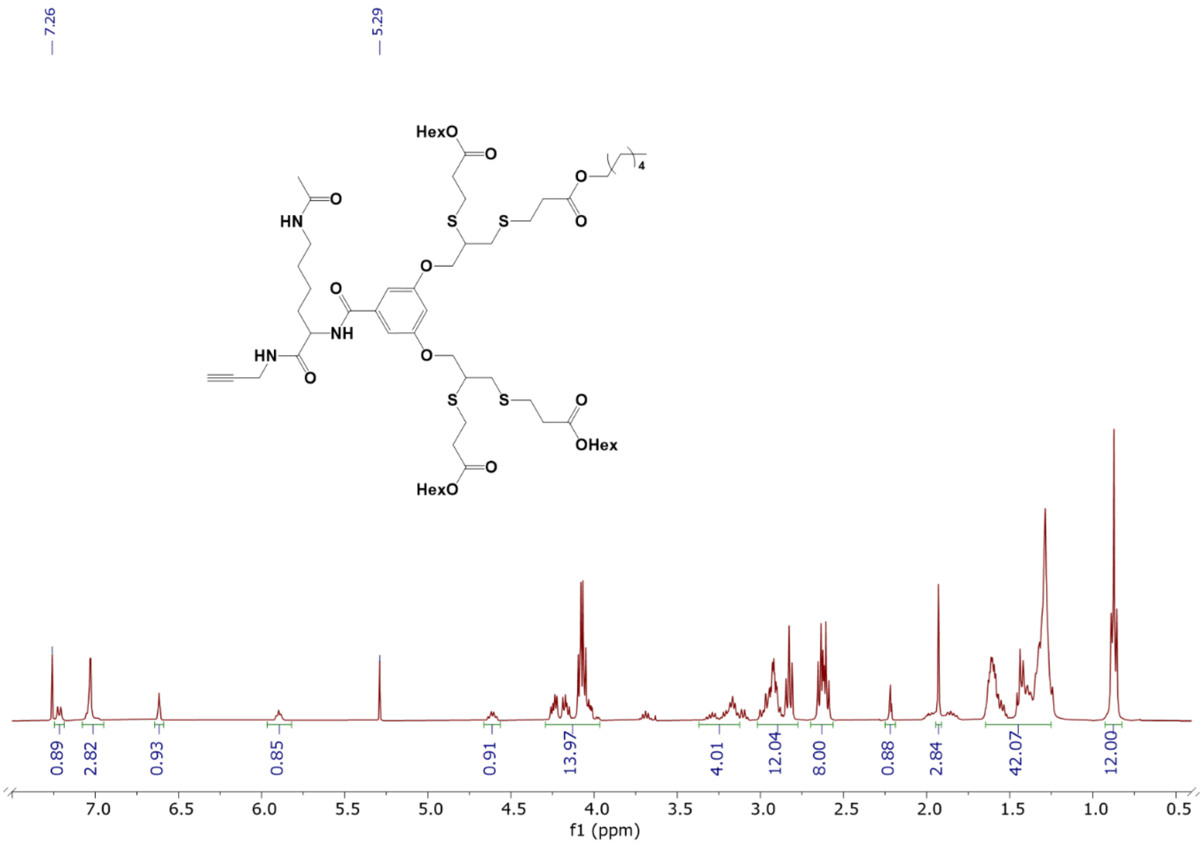

Figure S14: ^{1}H-NMR spectra of Lys(Acetyl)-dend-4xHex in $CDCl_3$.

Lys(Acetyl)-dend-4xOct

Lys(Boc)-dend-4xOct (70mg, 0.051mmol) was dissolved in DCM:TFA (1:1v/v) (1mL). Then, it was reacted according to the general procedure with DIPEA (25mg, 0.20mmol) and Acetyl chloride (4mg, 0.056mmol). Product was obtained as a colorless oil in 82% yield (56mg).

^{1}H-NMR (400MHz, $CDCl_3$): δ 7.18 (d, J = 7.6 Hz, 1H, -N**H**-CH_α-), 7.04 (d, J = 2.2 Hz, 2H, Ar-**H**), 6.91 (t, J = 5.4 Hz, 1H, -N**H**-CO-CH_3), 6.63 (t, J = 2.2 Hz, 1H, Ar-**H**), 5.80 (t, J = 6.0 Hz, 1H, -N**H**-CH_2-C≡CH), 4.64-4.52 (m, 1H, -NH–C**H**$_\alpha$-CO-NH-), 4.29-3.99 (m, 14H, Ar-O-C**H**$_2$- + CO-O-C**H**$_2$- + -NH-C**H**$_2$-C≡CH), 3.39-3.05 (m, 4H, -C**H**-S + -C**H**$_2$-NH-CO-CH_3), 3.03-2.79 (m, 12H, -C**H**$_2$-S-), 2.70-2.58 (m, 8H, C**H**$_2$-CO-O-), 2.22 (t, J = 2.5 Hz, 1H, -NH-CH_2-C≡C**H**), 1.93 (s, 3H, -NH-CO-C**H**$_3$), 1.70-1.09 (m, 54H, -CH_α-C**H**$_2$-C**H**$_2$-C**H**$_2$- + (C**H**$_2$)$_6$-CH_3), 0.94-0.79 (t, J= 6.4, 12H, -(CH_2)$_6$-C**H**$_3$).

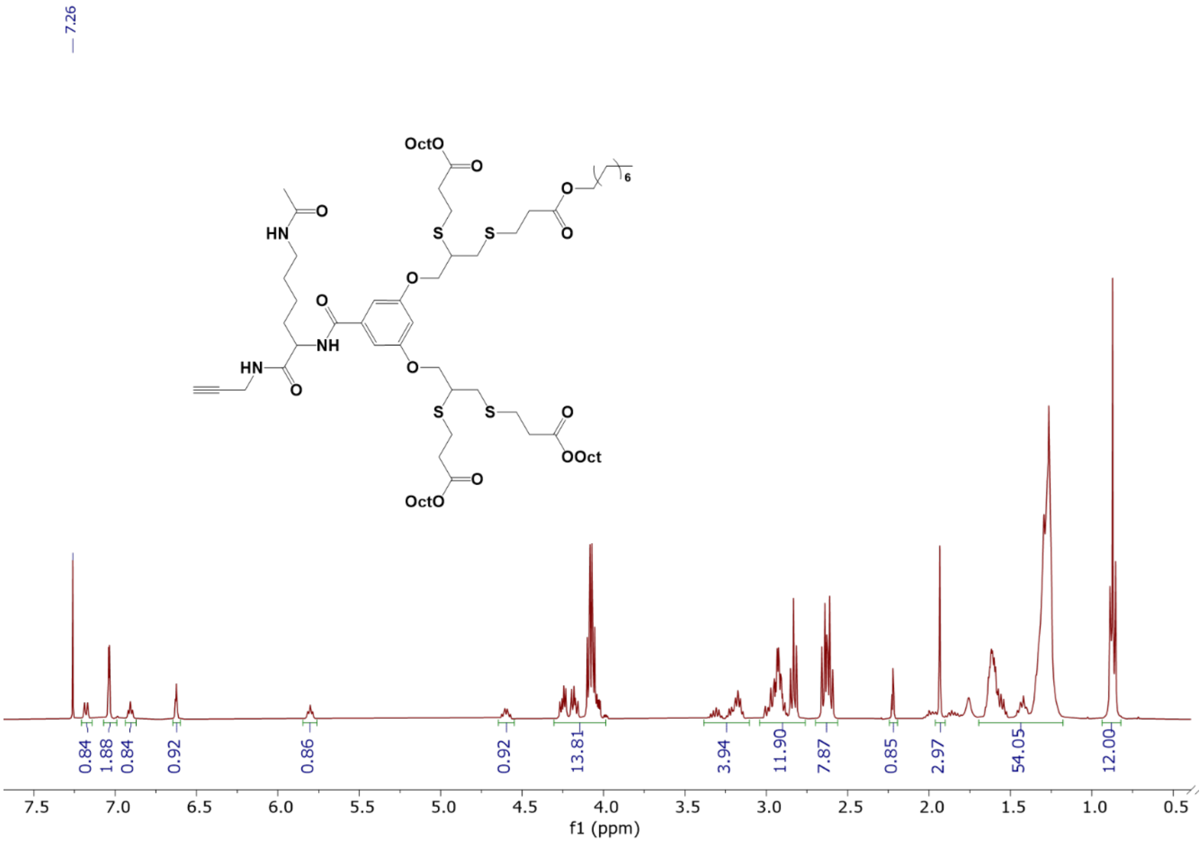

Figure S15: ^{1}H-NMR spectra of Lys(Acetyl)-dend-4xOct in $CDCl_3$.

Lys(Acetyl)-dend-4xDec

Lys(Boc)-dend-4xDec (70mg, 0.054 mmol) was dissolved in DCM:TFA (1:1v/v) (1mL). Then, it was reacted according to the general procedure with DIPEA (27mg, 0.21mmol) and Acetyl (4.5mg, 0.062mmol). Product was obtained as a colorless oil in 75% yield (61mg).

^{1}H-NMR (400MHz, $CDCl_3$): δ 7.15 (d, J = 7.5 Hz, 1H, -N**H**-CH_α-), 7.04 (d, J = 2.2 Hz, 2H, Ar-**H**), 6.82 (t, J = 5.4 Hz, 1H, -N**H**-CO-CH_3), 6.63 (t, J = 2.2 Hz, 1H, Ar-**H**), 5.76 (t, J = 6.0 Hz, 1H, -N**H**-CH_2-C≡CH), 4.59-4.49 (m, 1H, -NH–C**H**$_\alpha$-CO-NH-), 4.36-3.99 (m, 14H, Ar-O-C**H$_2$**- + CO-O-C**H$_2$**- + -NH-C**H$_2$**-C≡CH), 3.36-3.11 (m, 4H, -C**H**-S + -C**H$_2$**-NH-CO-CH_3), 3.07-2.78 (m, 12H, -C**H$_2$**-S-), 2.67-2.57 (m, 8H, C**H$_2$**-CO-O-), 2.22 (t, J = 2.5 Hz, 1H, -NH-CH_2-C≡C**H**), 1.94 (s, 3H, -NH-CO-C**H$_3$**), 1.70-1.15 (m, 70H, -CH_α-C**H$_2$**-C**H$_2$**-C**H$_2$**- + (C**H$_2$**)$_8$-CH_3), 0.90-0.81 (t, J= 6.5, 12H, -$(CH_2)_6$-C**H$_3$**).

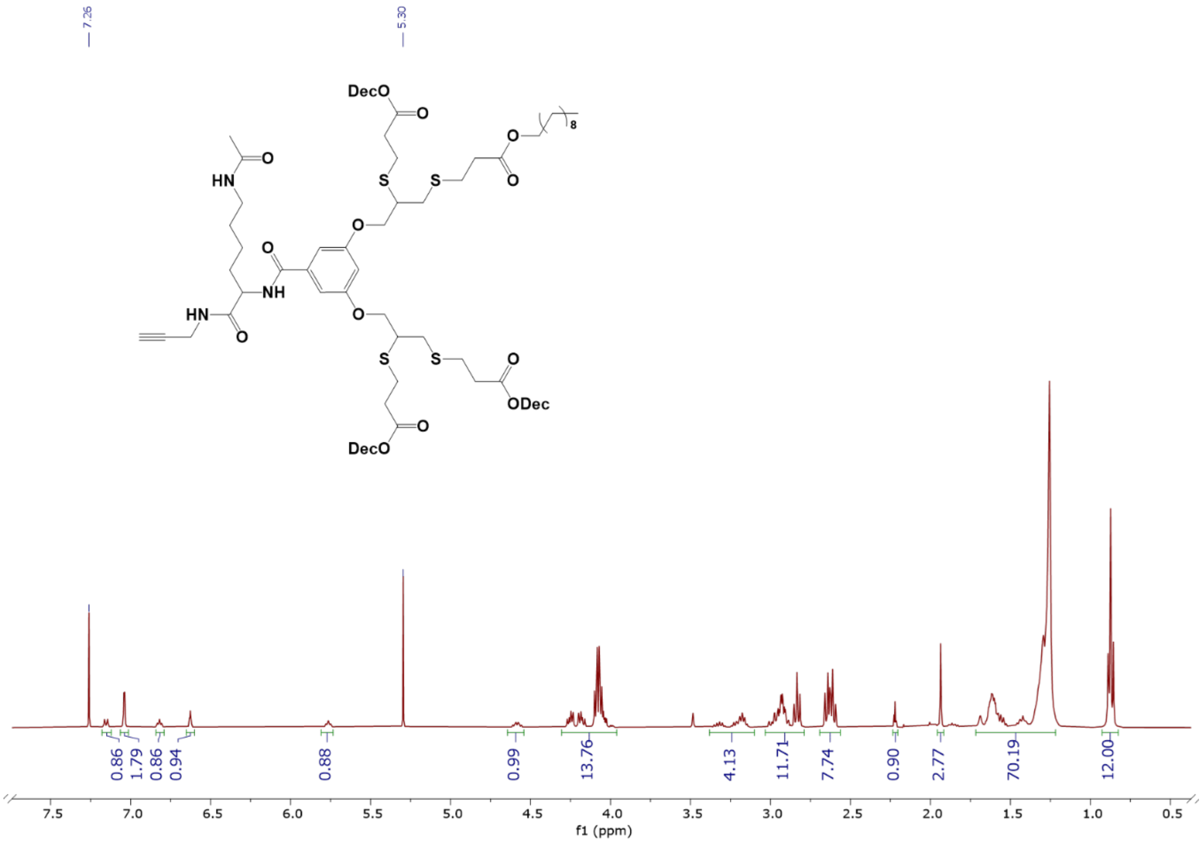

Figure S16: ^{1}H-NMR spectra of Lys(Acetyl)-dend-4xDec in $CDCl_3$.

Lys(Cy3)-dend-4Hex

Lys(Boc)-dend-4xHex (70mg, 0.054mmol) was dissolved in DCM:TFA (1:1v/v) (1mL). Then, it was reacted according to the general procedure with Cy3 (35 mg, 0.060 mmol), HBTU (22 mg, 0.060 mmol), and DIPEA (80 mg, 0.43mmol) in DCM:DMF (1:1 v/v) (3mL). Product was obtained as a pink oil in quantitative yield (62mg).

^{1}H-NMR (400MHz, $CDCl_3$): see following spectrum and assignments.

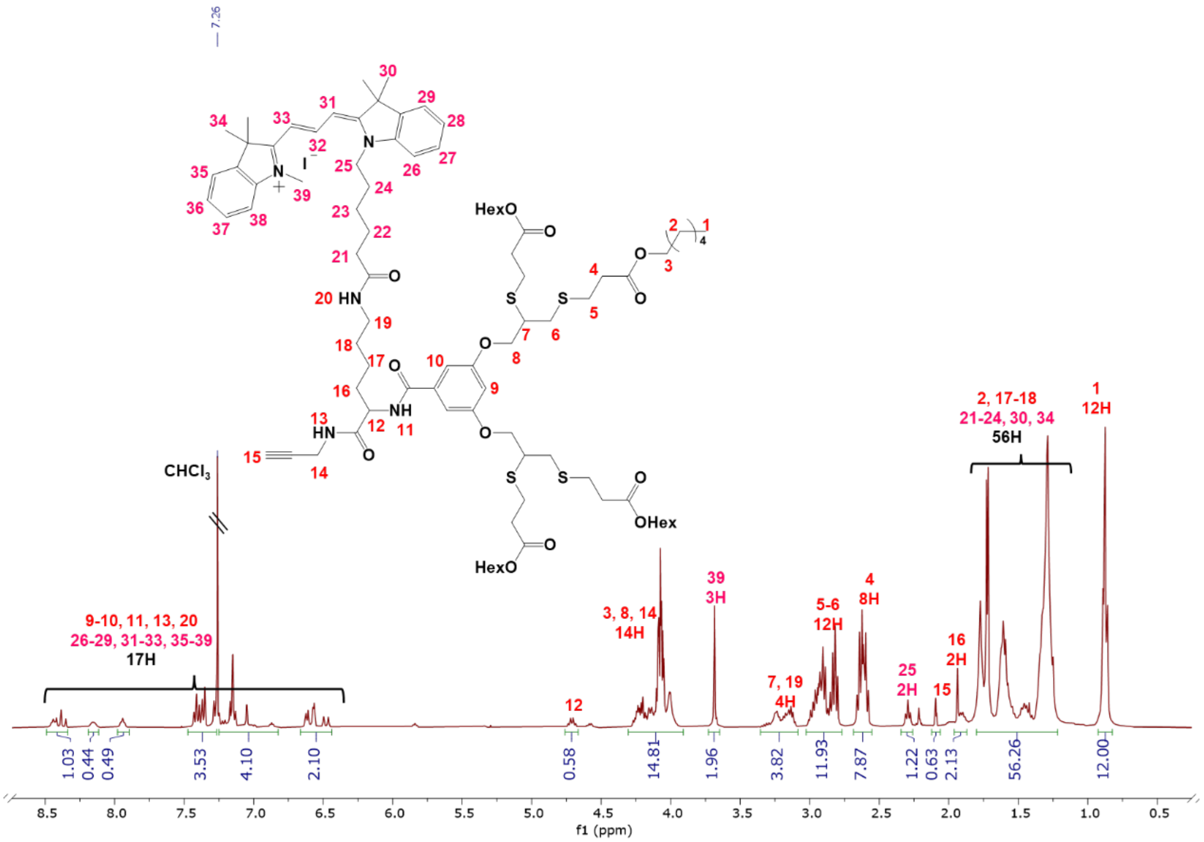

Figure S17: ^{1}H-NMR spectra of Lys(Cy3)-dend-4xHex in $CDCl_3$.

Lys(Cy3)-dend-4xOct

Lys(Boc)-dend-4xOct (96mg, 0.054mmol) was dissolved in DCM:TFA (1:1v/v) (1mL). Then, it was reacted according to the general procedure with Cy3 (45mg, 0.077mmol), HBTU (29mg, 0.077mmol), and DIPEA (80mg, 0.43mmol) in DCM:DMF (1:1 v/v) (4mL). Product was obtained as a pink oil in quantitative yield (123mg).

^{1}H-NMR (400MHz, $CDCl_3$): see following spectrum and assignments.

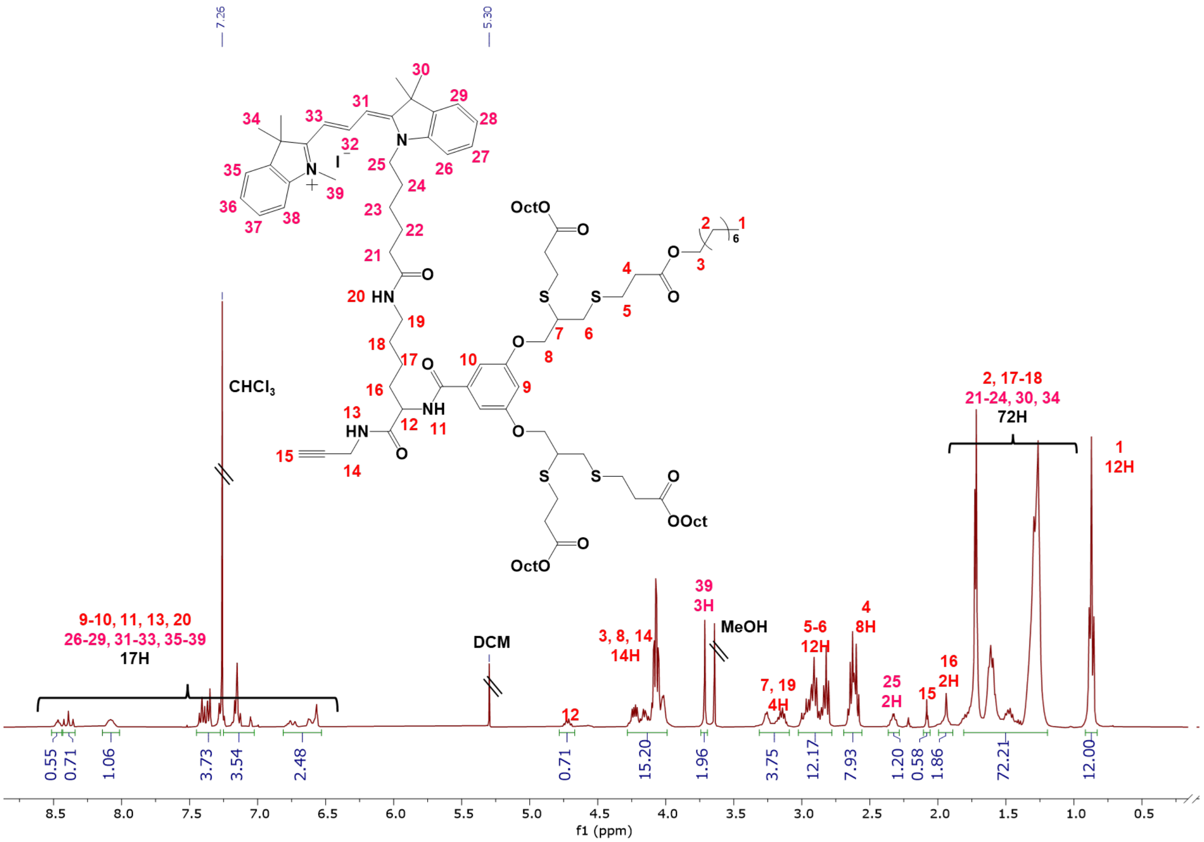

Figure S18: ^{1}H-NMR spectra of Lys(Cy3)-dend-4xOct in $CDCl_3$.

Lys(Cy3)-dend-4xDec

Lys(Boc)-dend-4xDec (54mg, 0.036mmol) was dissolved in DCM:TFA (1:1v/v) (1mL). Then, it was reacted according to the general procedure with Cy3 (25mg, 0.040mmol), HBTU (20mg, 0.040mmol), and DIPEA (60mg, 0.28mmol) in DCM:DMF (1:1 v/v) (4mL). Product was obtained as a pink oil in quantitative yield (66mg).

^{1}H-NMR (400MHz, $CDCl_3$): see following spectrum and assignments.

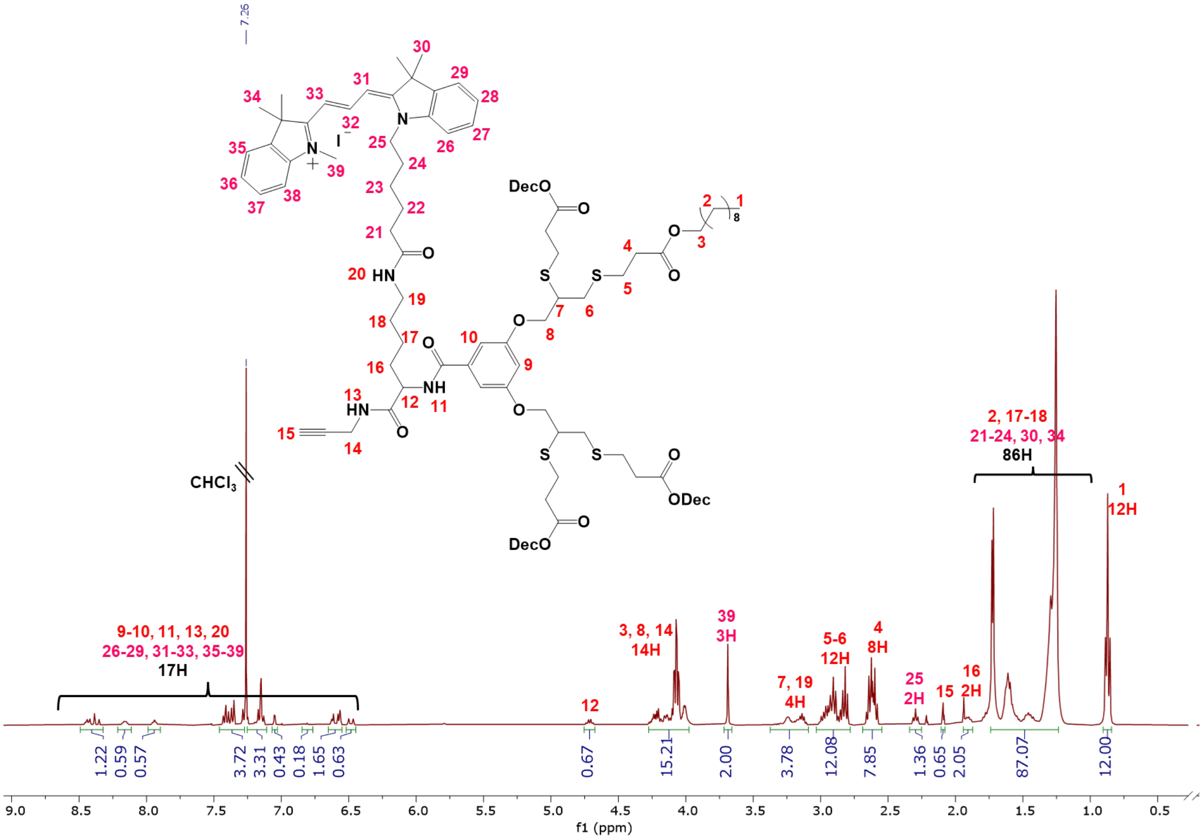

Figure S19: ^{1}H-NMR spectra of Lys(Cy3)-dend-4xDec in $CDCl_3$.

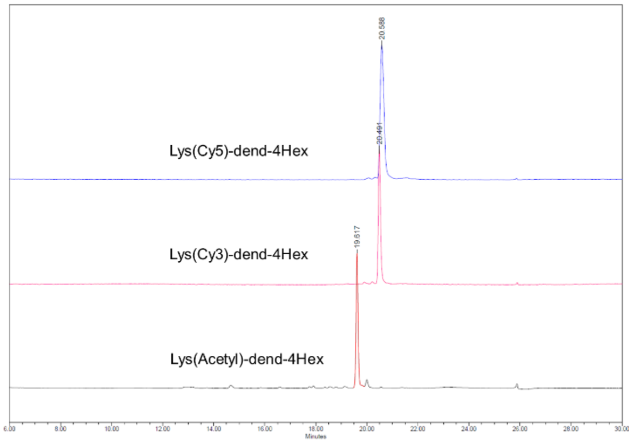

Figure S20: HPLC overlay of Lys(Acetyl)-dend-4xHex at 297nm, Lys(Cy3)-dend-4xHex at 546nm, and Lys(Cy5)-dend-4xHex at 640nm.

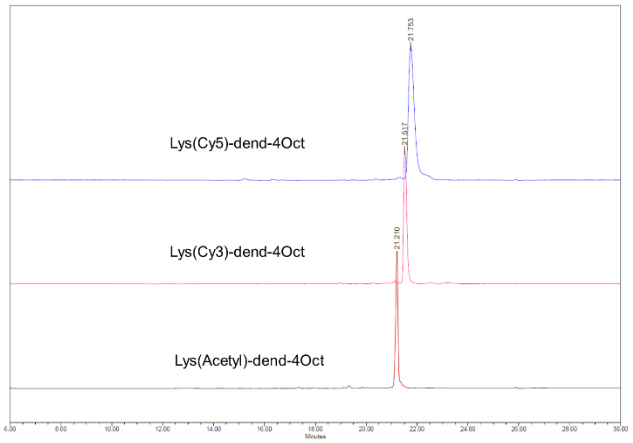

Figure S21: HPLC overlay of Lys(Acetyl)-dend-4xOct at 297nm, Lys(Cy3)-dend-4xOct at 546nm, and Lys(Cy5)-dend-4xOct at 640nm.

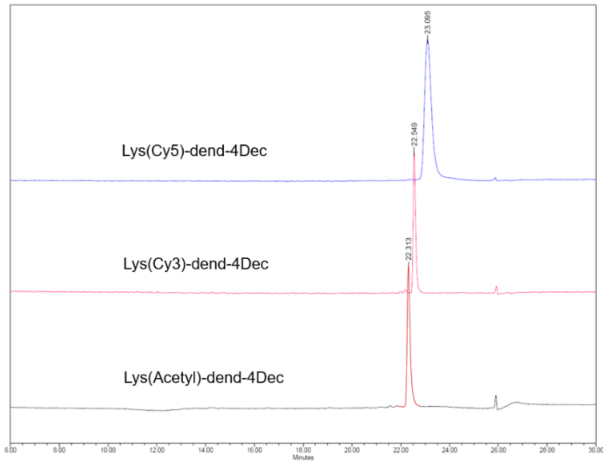

Figure S22: HPLC overlay of Lys(Acetyl)-dend-4xDec at 297nm, Lys(Cy3)-dend-4xDec at 546nm, and Lys(Cy5)-dend-4xDec at 640nm.

General procedure for CuAAC click reaction between di azide functionalized PtBA and esterase-responsive labelled dendron:

CuBr (3 eq. in respect to di azide functionalized PtBA) was loaded in 4 mL glass vial, which was sealed with a rubber septum. Vial was deoxygenated with three vacuum-nitrogen cycles and backfilled with nitrogen. In a separate 4 mL vial polymer-N_3 (1 eq.), dendron (2.6 eq.) and PMDETA (3 eq.) were dissolved in DMF (100 – 200 mg polymer/ mL) and purged with nitrogen for 2 minutes. The above-mentioned mixture was added into the CuBr containing vial using nitrogen flushed syringe and needle. Vial was thoroughly vortexed until clear green solution was obtained (approximately 30 seconds). Reaction was stirred at room temperature for 1 hour, filtered through syringe filter (0.44 µm, hydrophilic PTFE) and purified using LH20 (Sephadex®) size exclusion column and eluted with MeOH. Fractions that contained the product (identified by bright yellow color) were unified and MeOH evaporated to dryness and product was dried on high vacuum. All polymers were obtained as solids, with distinctive blue, pink or white color according to dendron labeling (Cy5, Cy3 and acetyl, accordingly).

SS-PtBA-L(Ac)-dend-4xHex

CuBr (6 mg), SS-PtBA-N_3 (92mg), Lys(Acetyl)-dend-4xHex (50 mg) and PMDETA (9 µL) were reacted according to the general procedure. The product was obtained as yellow solid in 93% yield (118 mg). ^{1}H NMR (400 MHz, Chloroform-*d*): see following spectrum and assignments.

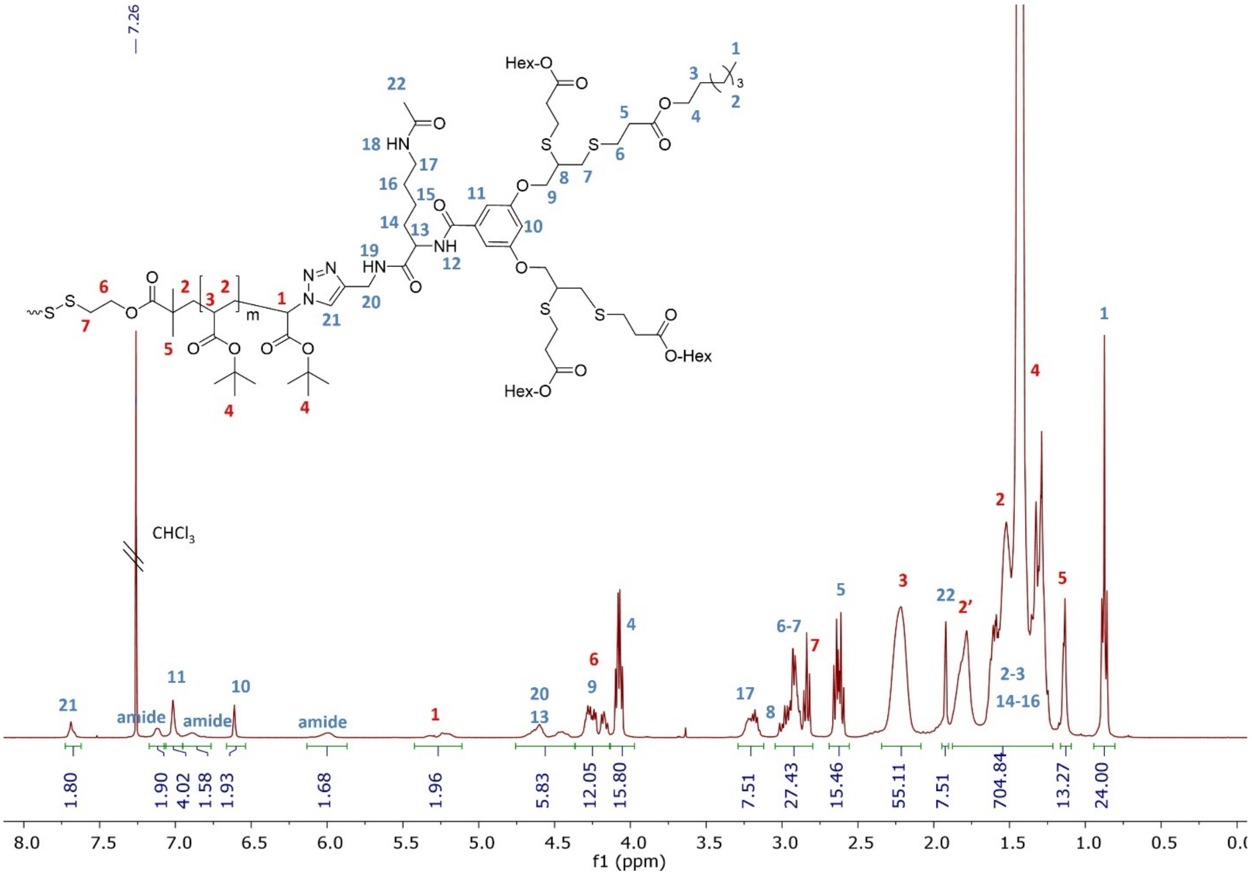

Figure S23: ^{1}H-NMR spectra of SS-PtBA-L(Ac)-dend-4xHex in $CDCl_3$.

SS-PtBA-L(Cy3)-dend-4xHex

CuBr (6 mg), SS-PtBA-N_3 (93 mg), Lys(Cy3)-dend-4xHex (55 mg) and PMDETA (9 µL) were reacted according to the general procedure. The product was obtained as yellow solid in 90% yield (107 mg). ^{1}H NMR (400 MHz, Chloroform-*d*): see following spectrum and assignments.

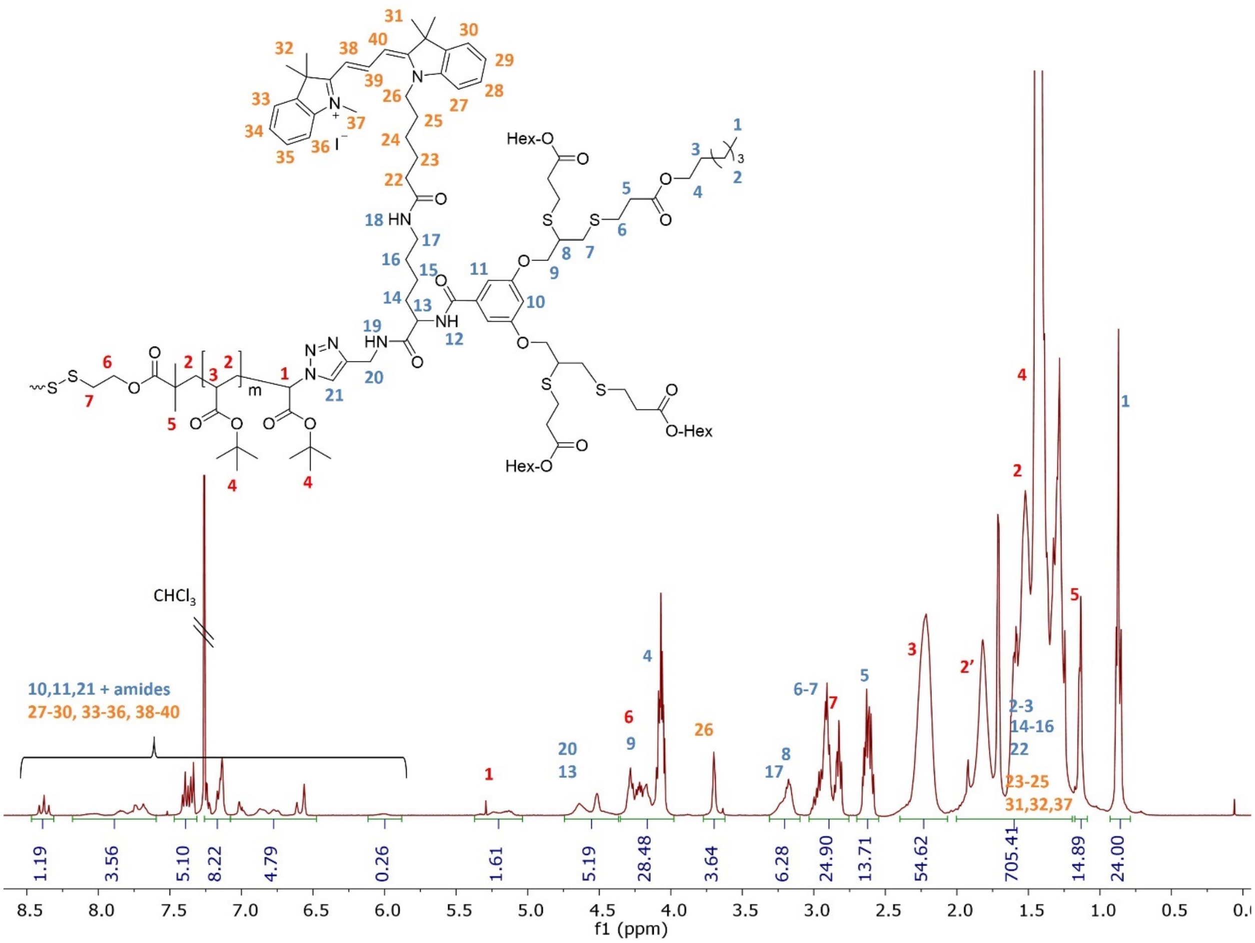

Figure S24: ^{1}H-NMR spectra of SS-PtBA-L(Cy3)-dend-4xHex in $CDCl_3$.

SS-PtBA-L(Cy5)-dend-4xHex

CuBr (6 mg), SS-PtBA-N_3 (96 mg), Lys(Cy5)-dend-4xHex (60 mg) and PMDETA (9 µL) were reacted according to the general procedure. The product was obtained as yellow solid in 94% yield (120 mg). ^{1}H NMR (400 MHz, Chloroform-*d*): see following spectrum and assignments.

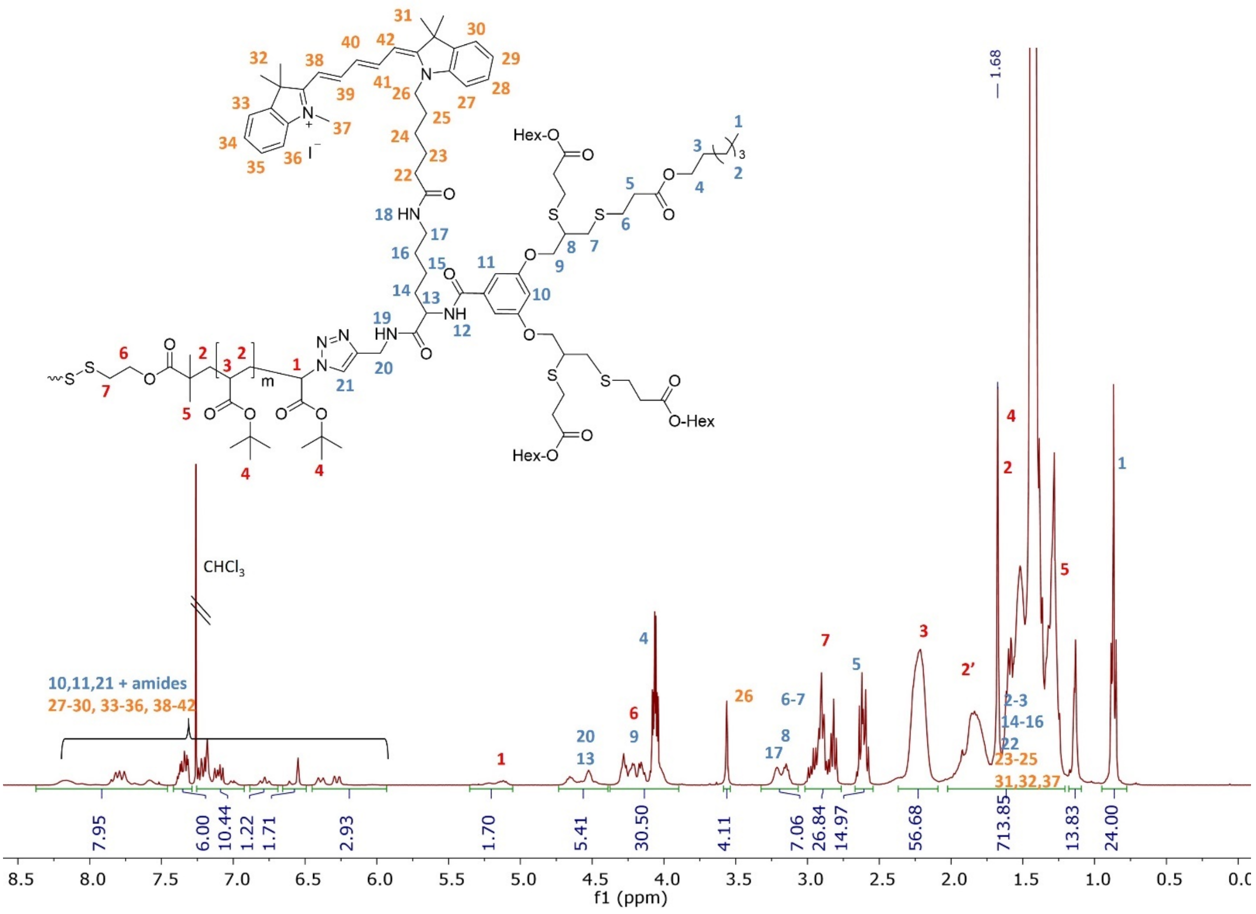

Figure S25: ^{1}H-NMR spectra of SS-PtBA-L(Cy5)-dend-4xHex in $CDCl_3$.

SS-PtBA-L(Ac)-dend-4xOct

CuBr (6 mg), SS-PtBA-N_3 (93mg), Lys(Acetyl)-dend-4xOct (60 mg) and PMDETA (9 µL) were reacted according to the general procedure. The product was obtained as yellow solid in 91% yield (112 mg). ^{1}H NMR (400 MHz, Chloroform-*d*): see following spectrum and assignments.

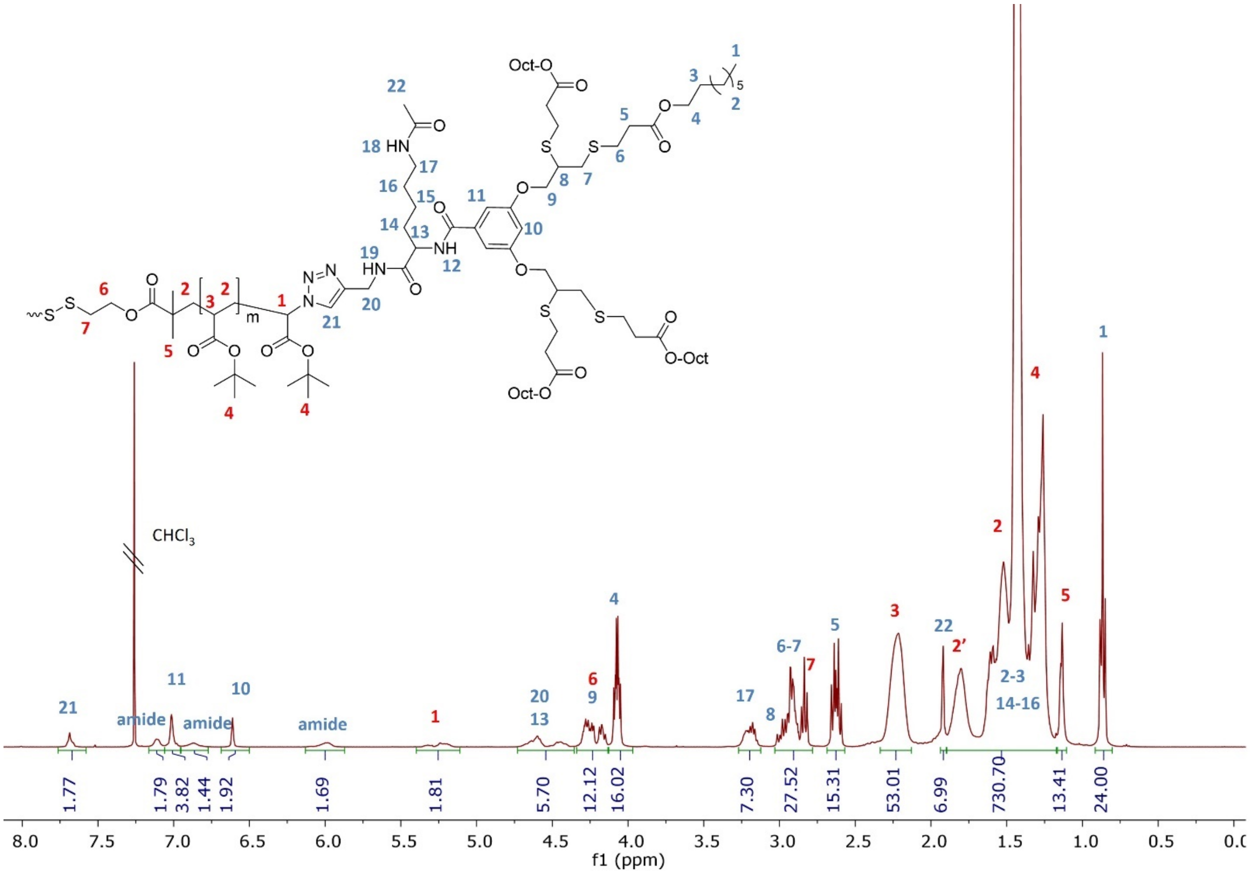

Figure S26: ^{1}H-NMR spectra of SS-PtBA-L(Ac)-dend-4xOct in $CDCl_3$.

SS-PtBA-L(Cy3)-dend-4xOct

CuBr (6 mg), SS-PtBA-N_3 (96 mg), Lys(Cy3)-dend-4xOct(58 mg) and PMDETA (9 µL) were reacted according to the general procedure. The product was obtained as yellow solid in quantitative yield (129 mg). ^{1}H NMR (400 MHz, Chloroform-*d*): see following spectrum and assignments.

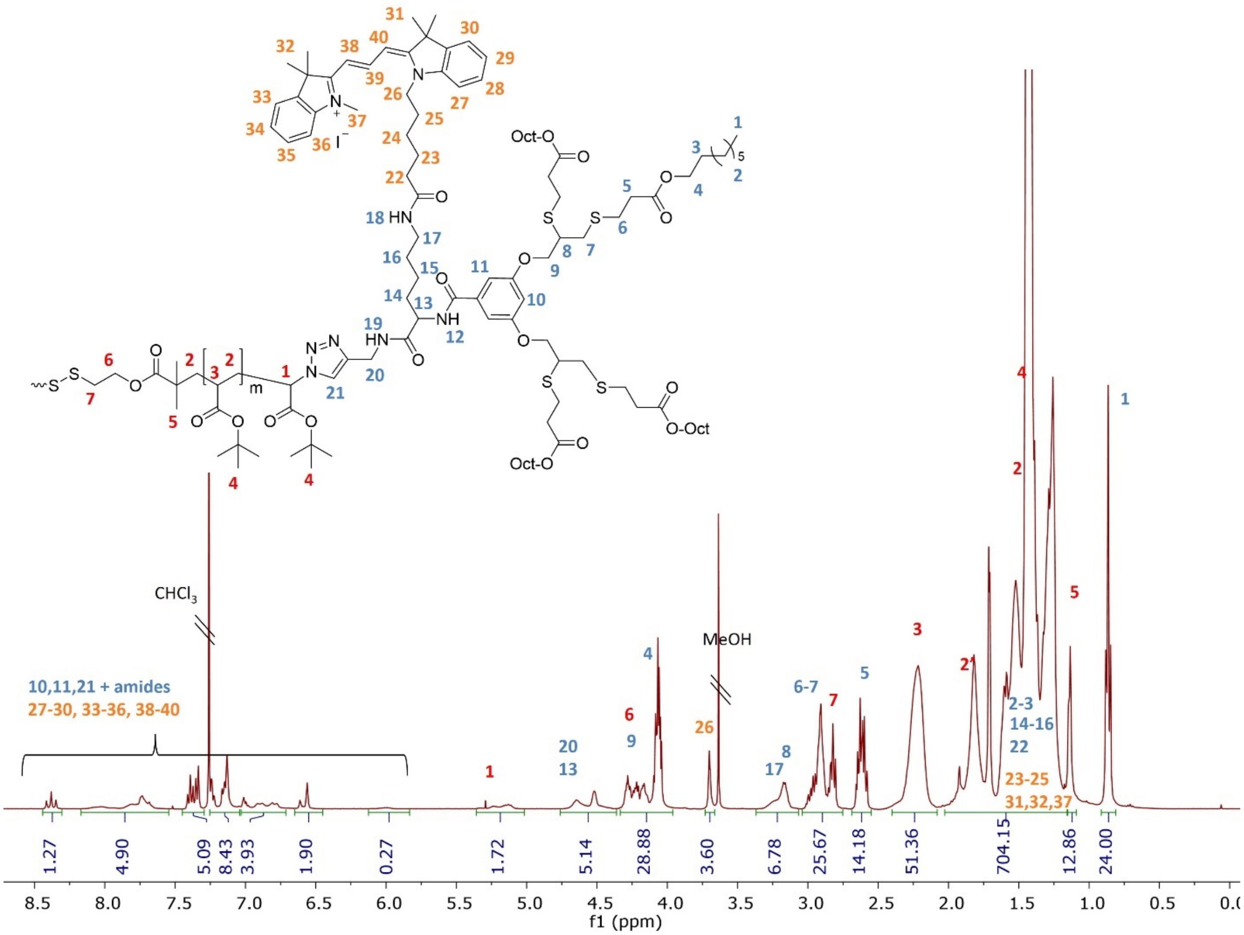

Figure S27: ^{1}H-NMR spectra of SS-PtBA-L(Cy3)-dend-4xOct in $CDCl_3$.

SS-PtBA-L(Cy5)-dend-4xOct

CuBr (6 mg), SS-PtBA-N_3 (92 mg), Lys(Cy3)-dend-4xOct (53 mg) and PMDETA (9 µL) were reacted according to the general procedure. The product was obtained as yellow solid in 93% yield (117 mg). ^{1}H NMR (400 MHz, Chloroform-*d*): see following spectrum and assignments.

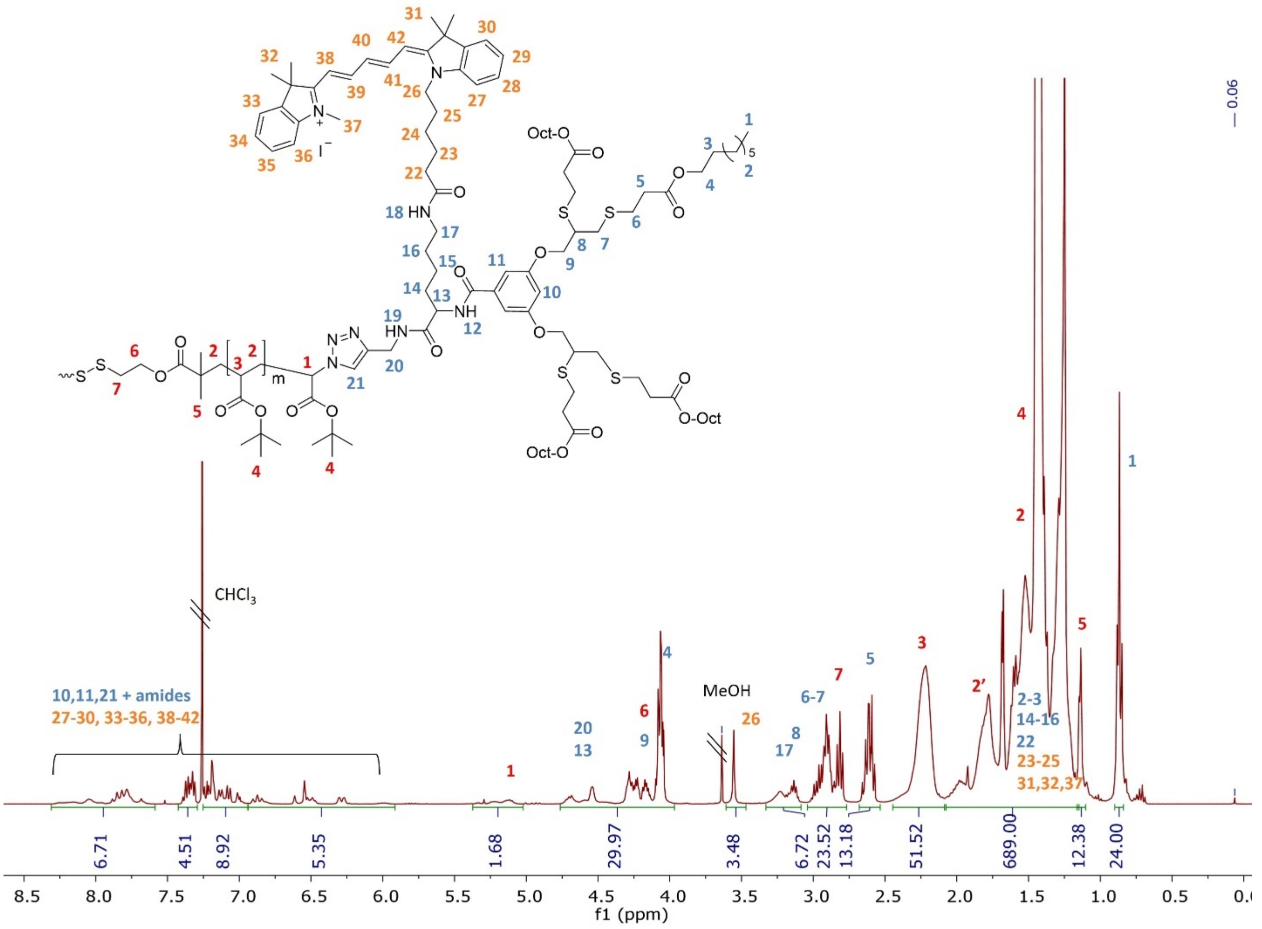

Figure S28: ^{1}H-NMR spectra of SS-PtBA-L(Cy5)-dend-4xOct in $CDCl_3$.

SS-PtBA-L(Ac)-dend-4xDec

CuBr (6 mg), SS-PtBA-N_3 (95mg), Lys(Acetyl)-dend-4xDec (60 mg) and PMDETA (9 µL) were reacted according to the general procedure. The product was obtained as yellow solid in 93% yield (119 mg). ^{1}H NMR (400 MHz, Chloroform-*d*): see following spectrum and assignments.

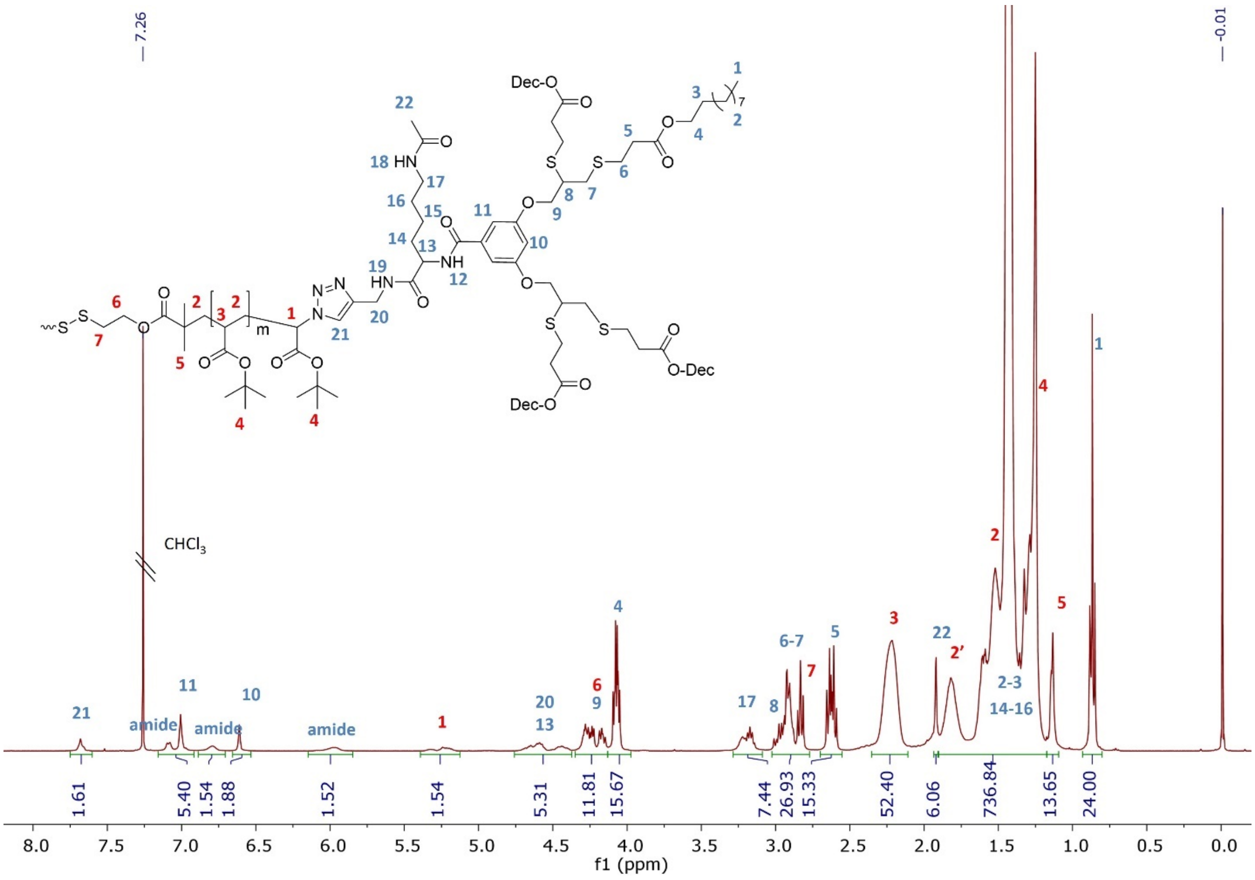

Figure S29: ^{1}H-NMR spectra of SS-PtBA-L(Ac)-dend-4xDec in $CDCl_3$.

SS-PtBA-L(Cy3)-dend-4xDec

CuBr (6 mg), SS-PtBA-N_3 (92 mg), Lys(Cy3)-dend-4xDec (58 mg) and PMDETA (9 µL) were reacted according to the general procedure. The product was obtained as yellow solid in quantitative yield (124 mg). ^{1}H NMR (400 MHz, Chloroform-*d*): see following spectrum and assignments.

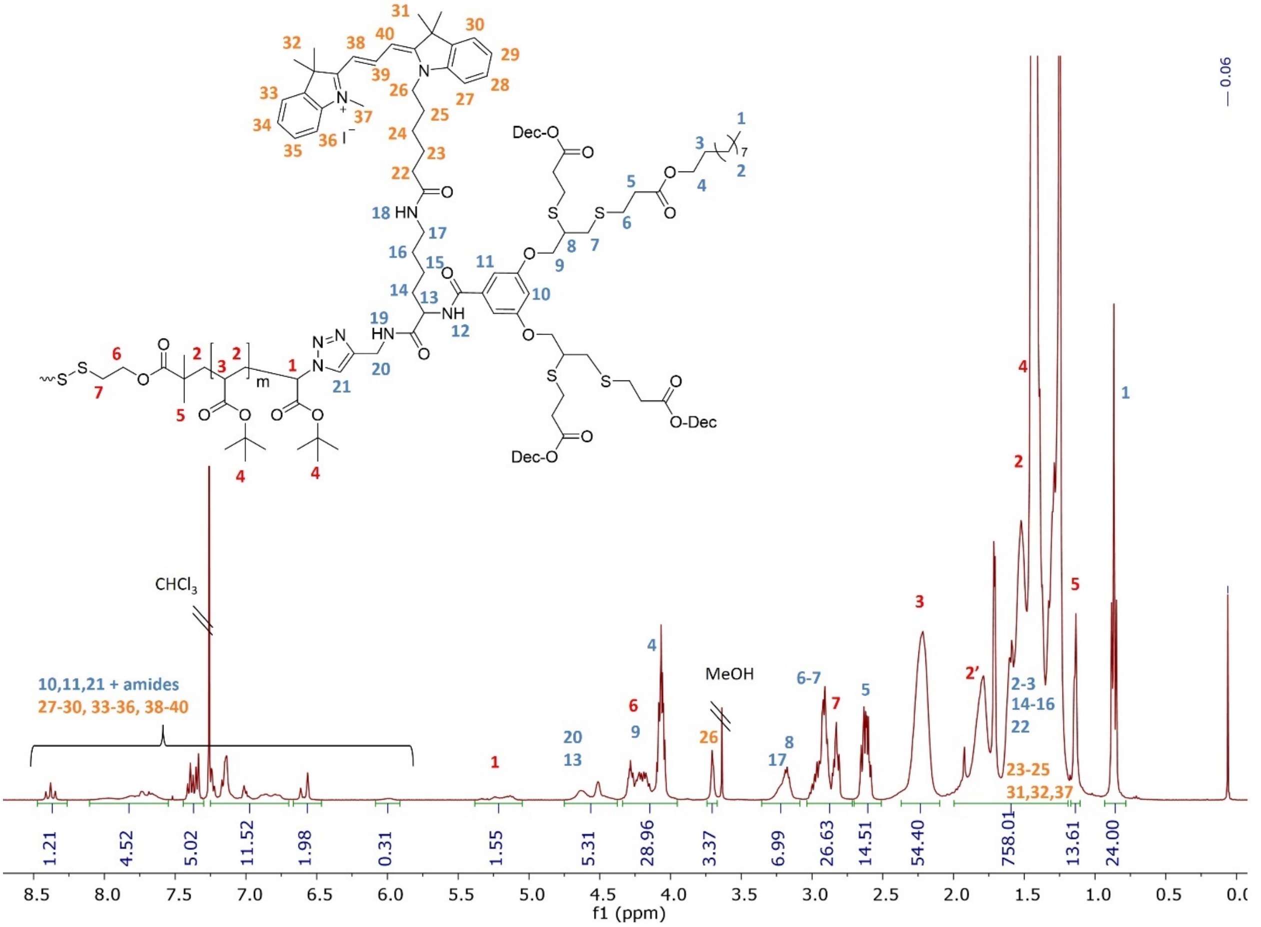

Figure S30: ^{1}H-NMR spectra of SS-PtBA-L(Cy3)-dend-4xDec in $CDCl_3$.

SS-PtBA-L(Cy5)-dend-4xDec

CuBr (6 mg), SS-PtBA-N_3 (92 mg), Lys(Cy5)-dend-4xDec (60 mg) and PMDETA (9 µL) were reacted according to the general procedure. The product was obtained as yellow solid in quantitative yield (126 mg). ^{1}H NMR (400 MHz, Chloroform-*d*): see following spectrum and assignments.

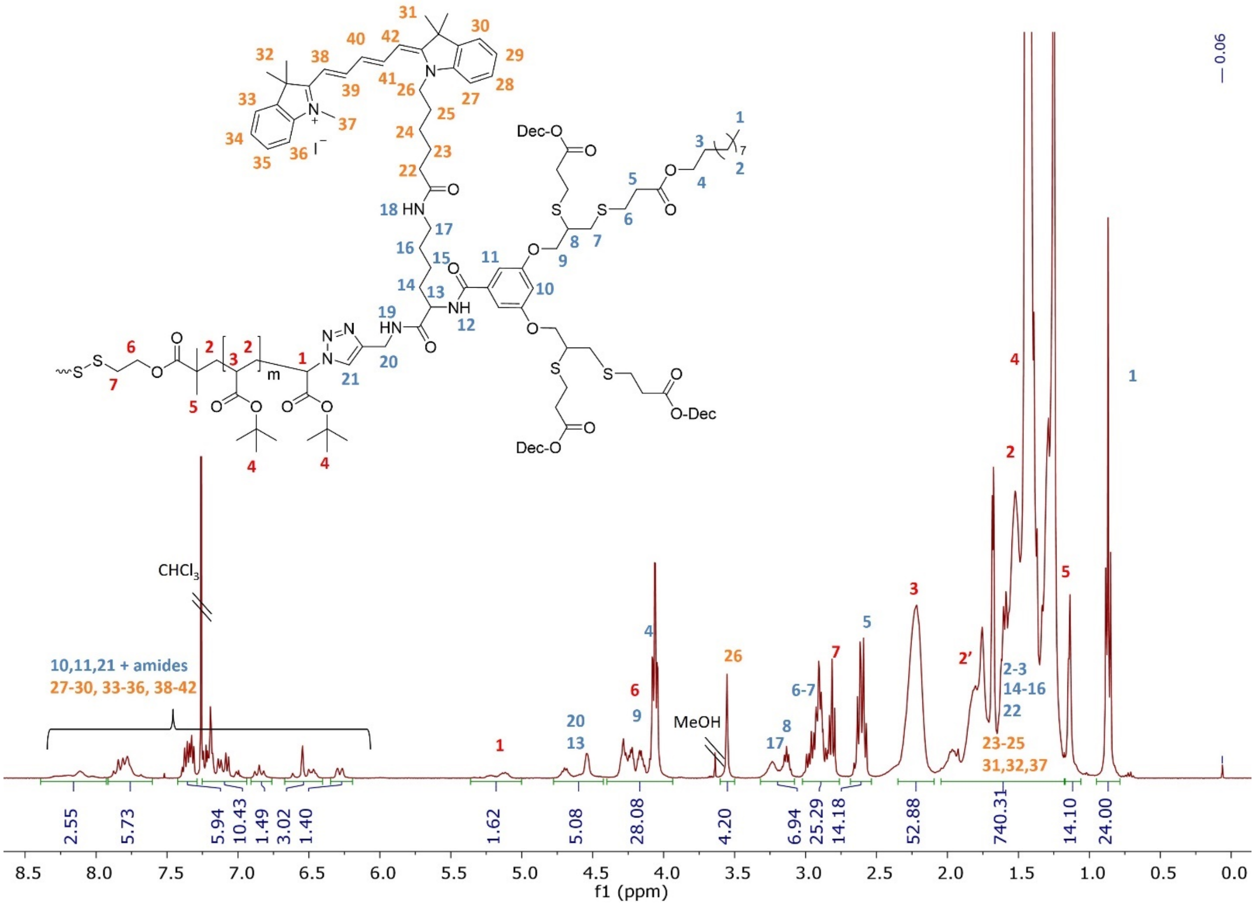

Figure S31: ^{1}H-NMR spectra of SS-PtBA-L(Cy5)-dend-4xDec in $CDCl_3$.

General procedure for tert-butyl deprotection of PtBA:

Tert butyl protection was removed by dissolving ~100 mg of SS-PtBA-L(X)-dend-4xAlkyl in 3 ml TFA and stirring at room temperature for one hour. TFA was removed under reduced pressure and further dried under high vacuum for 30 minutes. Product was redissolved in MeOH and further purified by LH20 SEC, to afford the final TBA amphiphiles (SS-PAA-L(X)-dend-4xAlkyl). Due to the treatment with TFA, the TBA amphiphiles were partially splitted into DBA. All fractions were analyzed using HPLC (at the relevant wavelength), and those with sufficient purity (above 95% TBA) were collected and used for further experiments and analysis.

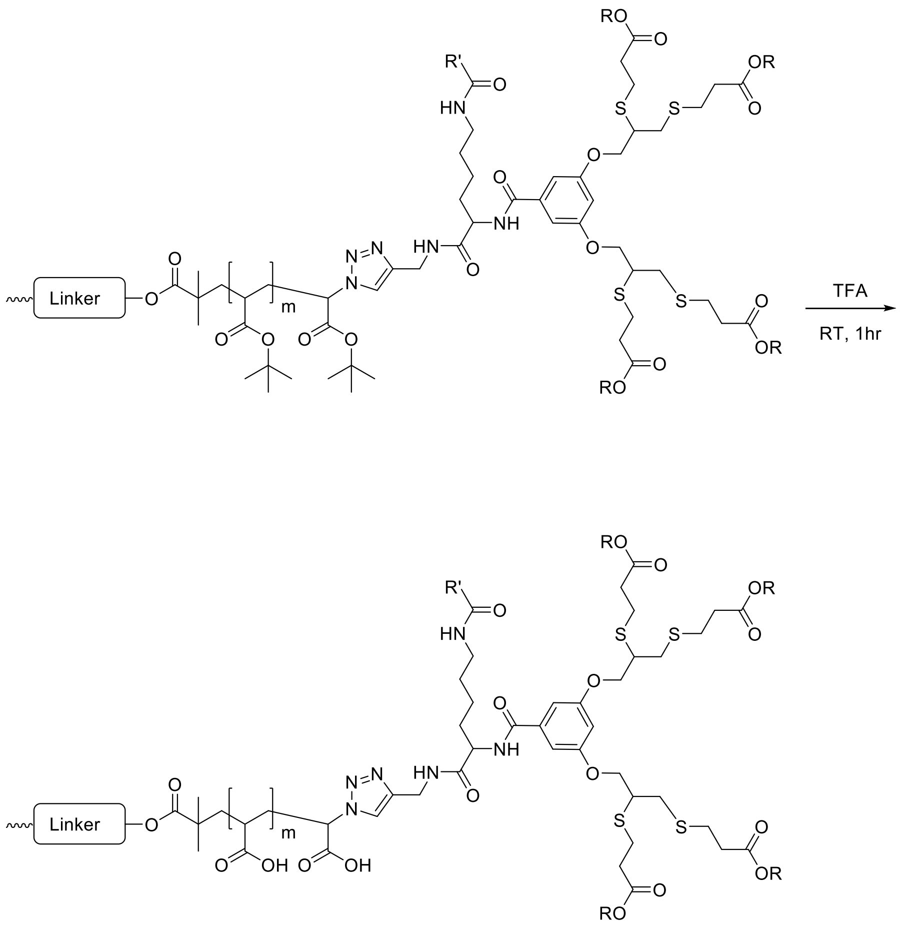

Figure S32: Synthetic scheme for the tert-butyl deprotection of SS-PtBA-L(X)-dend-4xAlkyl.

SS-PAA-L(Ac)-dend-4xHex

SS-PtBA-L(Ac)-dend-4xHex was treated with TFA according to the general procedure. ^{1}H-NMR (400 MHz, CD_3OD): see following spectrum and assignments.

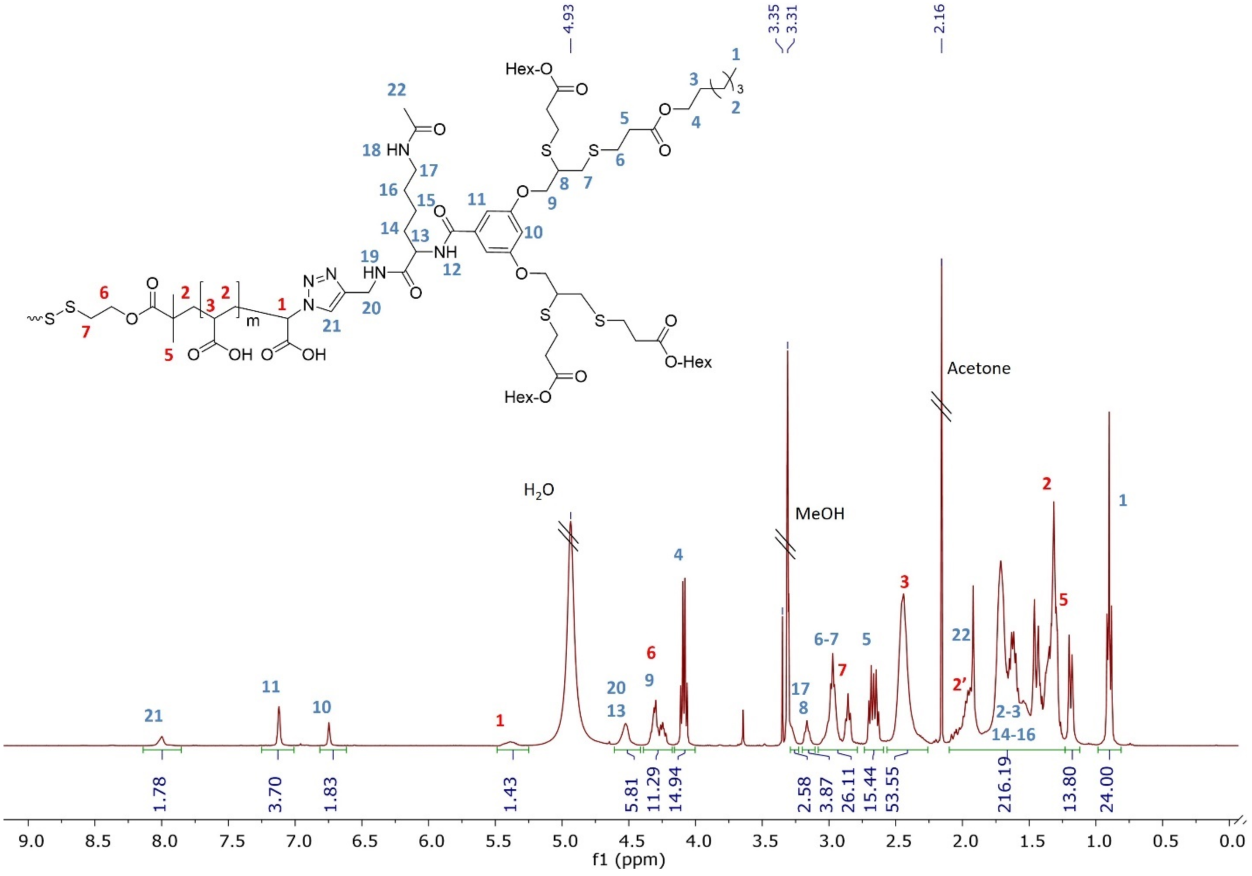

Figure S33: ^{1}H-NMR spectra of SS-PAA-L(Ac)-dend-4xHex in CD_3OD.

SS-PAA-L(Cy3)-dend-4xHex

SS-PtBA-L(Cy3)-dend-4xHex was treated with TFA according to the general procedure. ^{1}H-NMR (400 MHz, CD_3OD): see following spectrum and assignments.

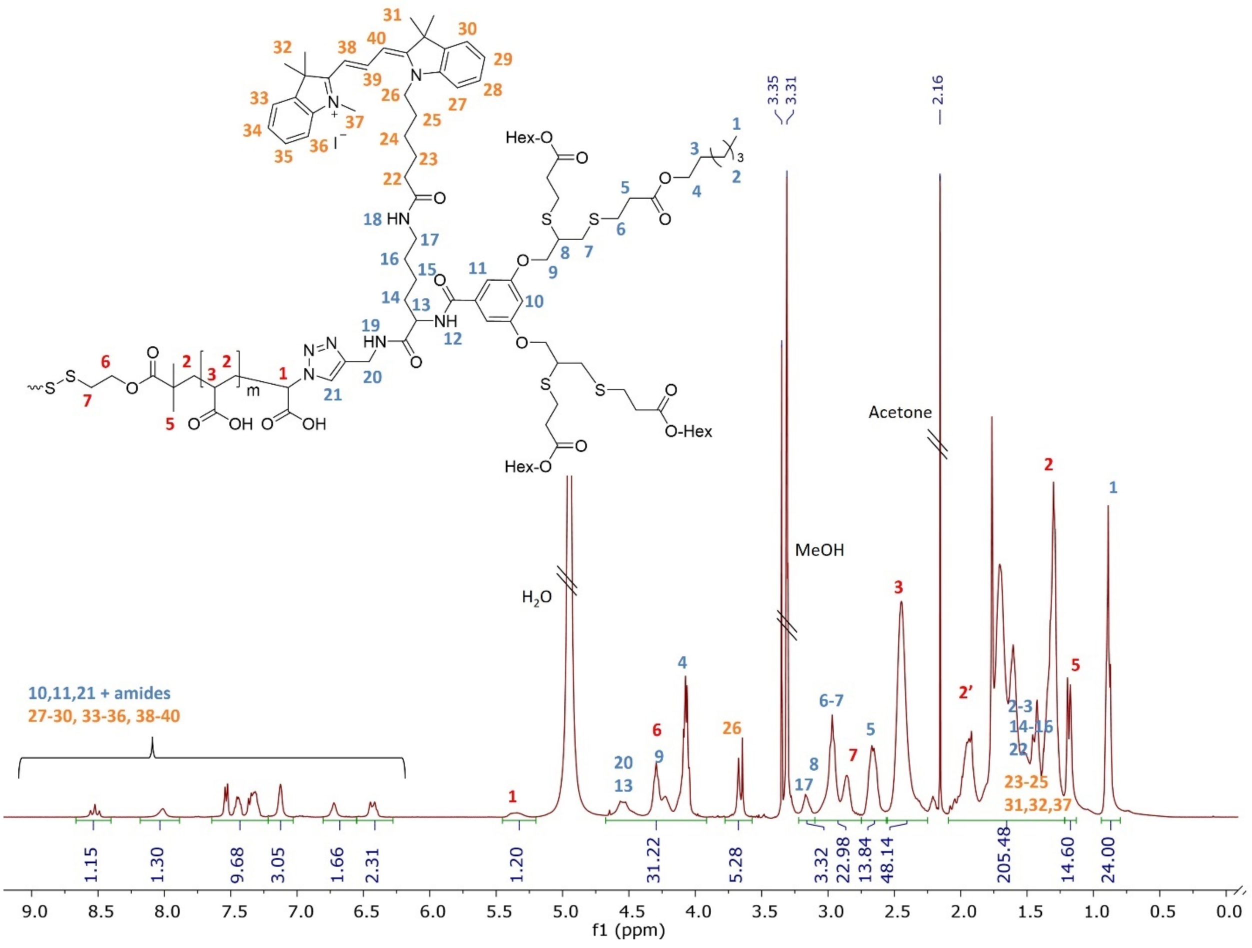

Figure S34: ^{1}H-NMR spectra of SS-PAA-L(Cy3)-dend-4xHex in CD_3OD.

SS-PAA-L(Cy5)-dend-4xHex

SS-PtBA-L(Cy5)-dend-4xHex was treated with TFA according to the general procedure. ^{1}H-NMR (400 MHz, CD_3OD): see following spectrum and assignments.

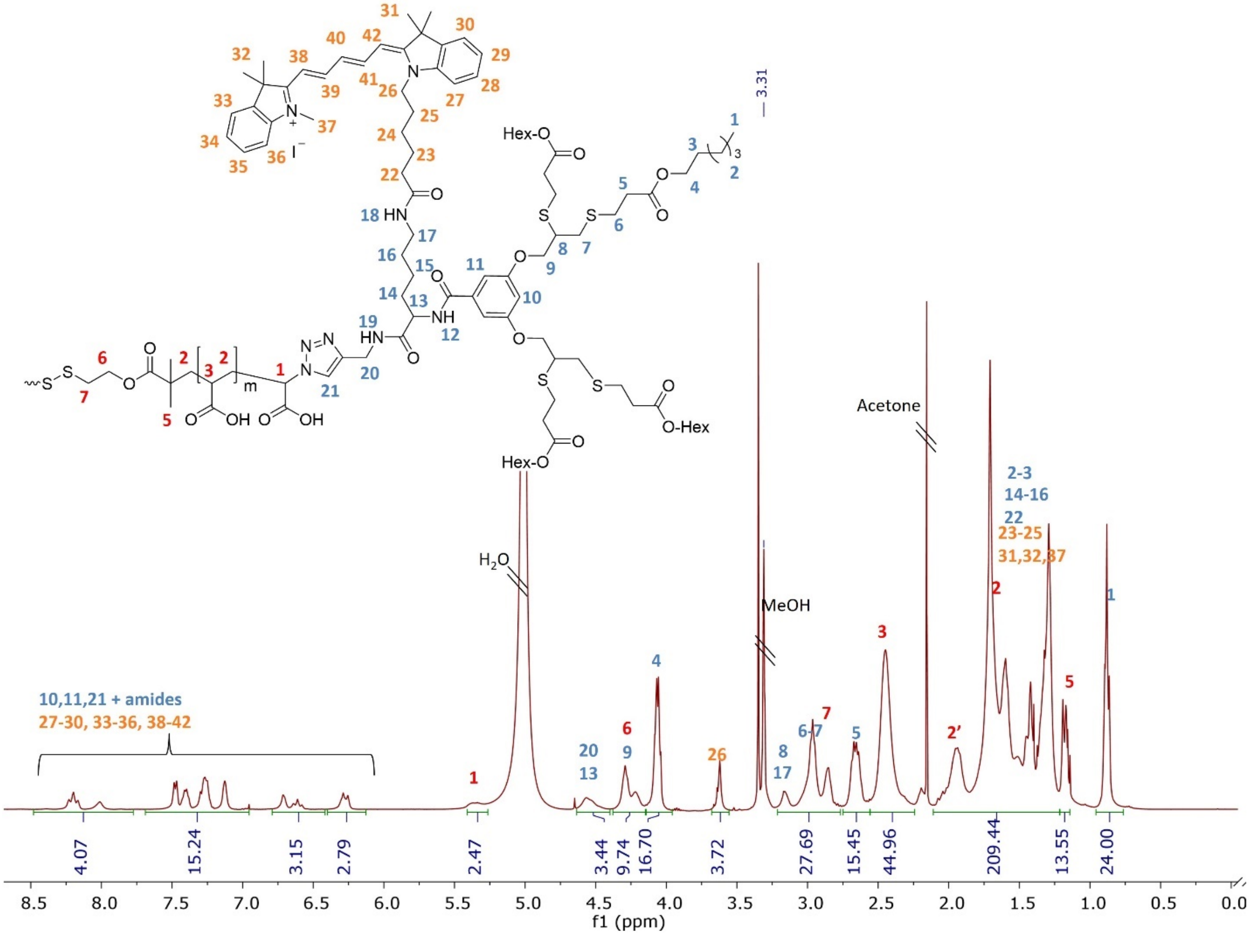

Figure S35: ^{1}H-NMR spectra of SS-PAA-L(Cy5)-dend-4xHex in CD_3OD.

SS-PAA-L(Ac)-dend-4xOct

SS-PtBA-L(Ac)-dend-4xOct was treated with TFA according to the general procedure. ^{1}H-NMR (400 MHz, CD_3OD): see following spectrum and assignments.

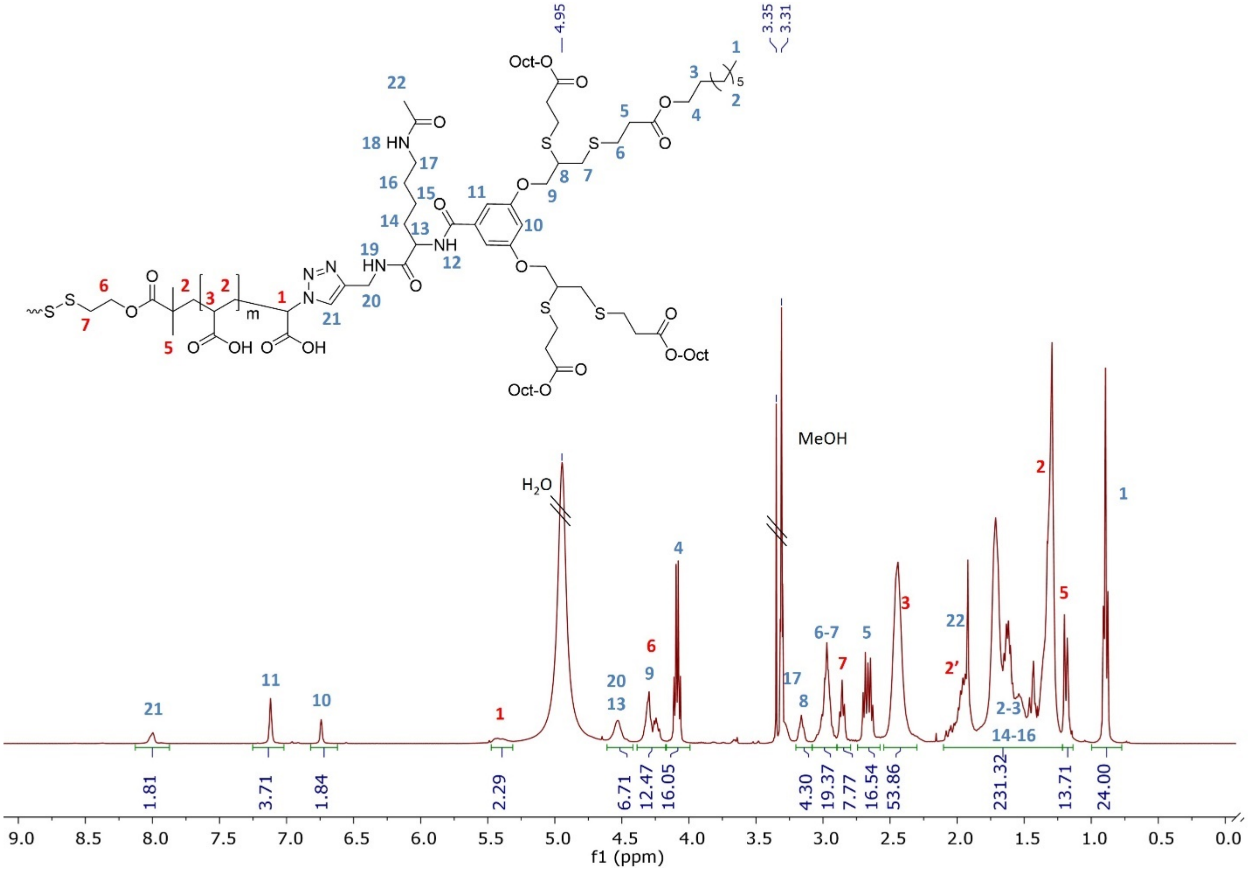

Figure S36: ^{1}H-NMR spectra of SS-PAA-L(Ac)-dend-4xOct in CD_3OD.

SS-PAA-L(Cy3)-dend-4xOct

SS-PtBA-L(Cy3)-dend-4xOct was treated with TFA according to the general procedure. ^{1}H-NMR (400 MHz, CD_3OD): see following spectrum and assignments.

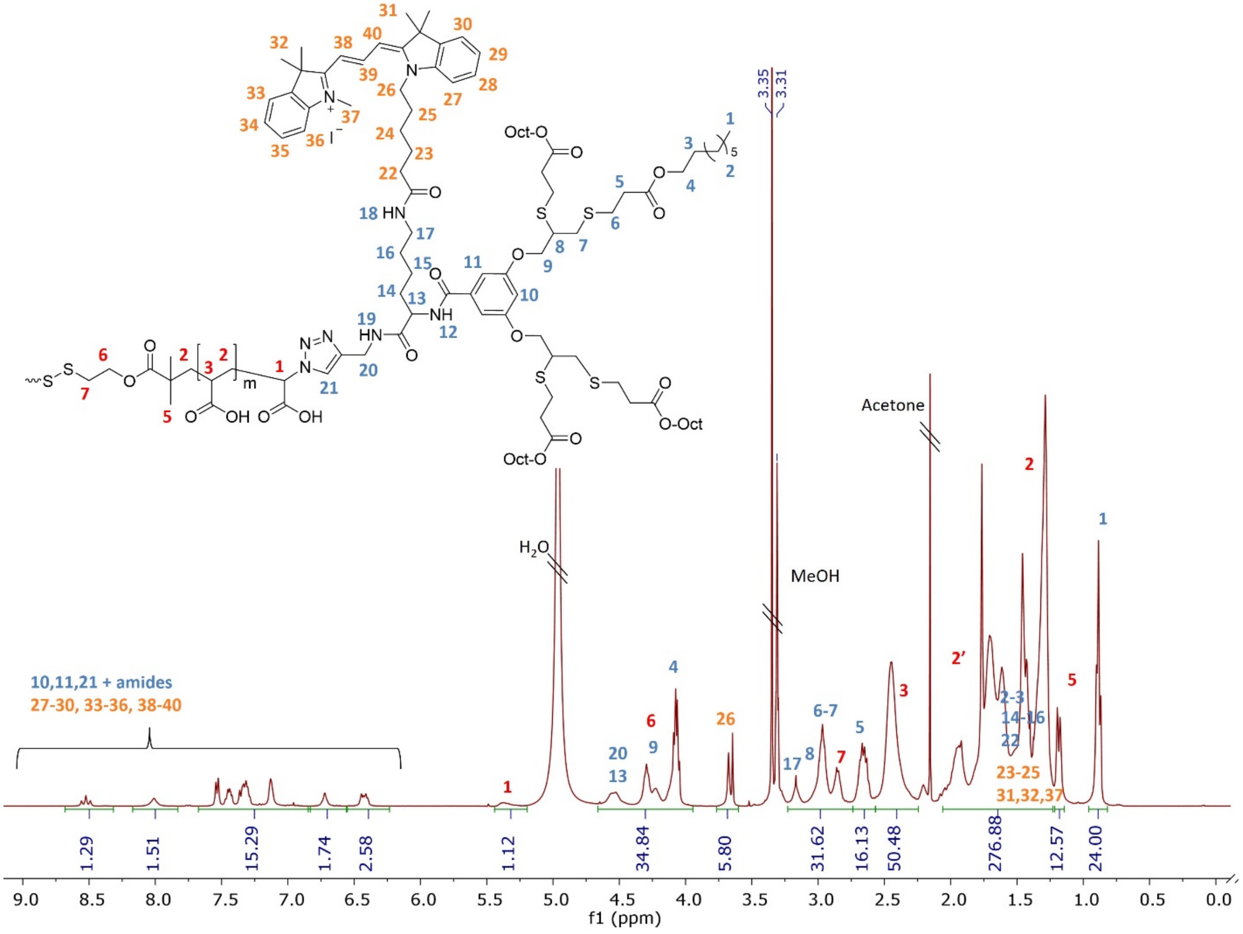

Figure S37: ^{1}H-NMR spectra of SS-PAA-L(Cy3)-dend-4xOct in CD_3OD.

SS-PAA-L(Cy5)-dend-4xOct

SS-PtBA-L(Cy5)-dend-4xOct was treated with TFA according to the general procedure. ^{1}H-NMR (400 MHz, CD_3OD): see following spectrum and assignments.

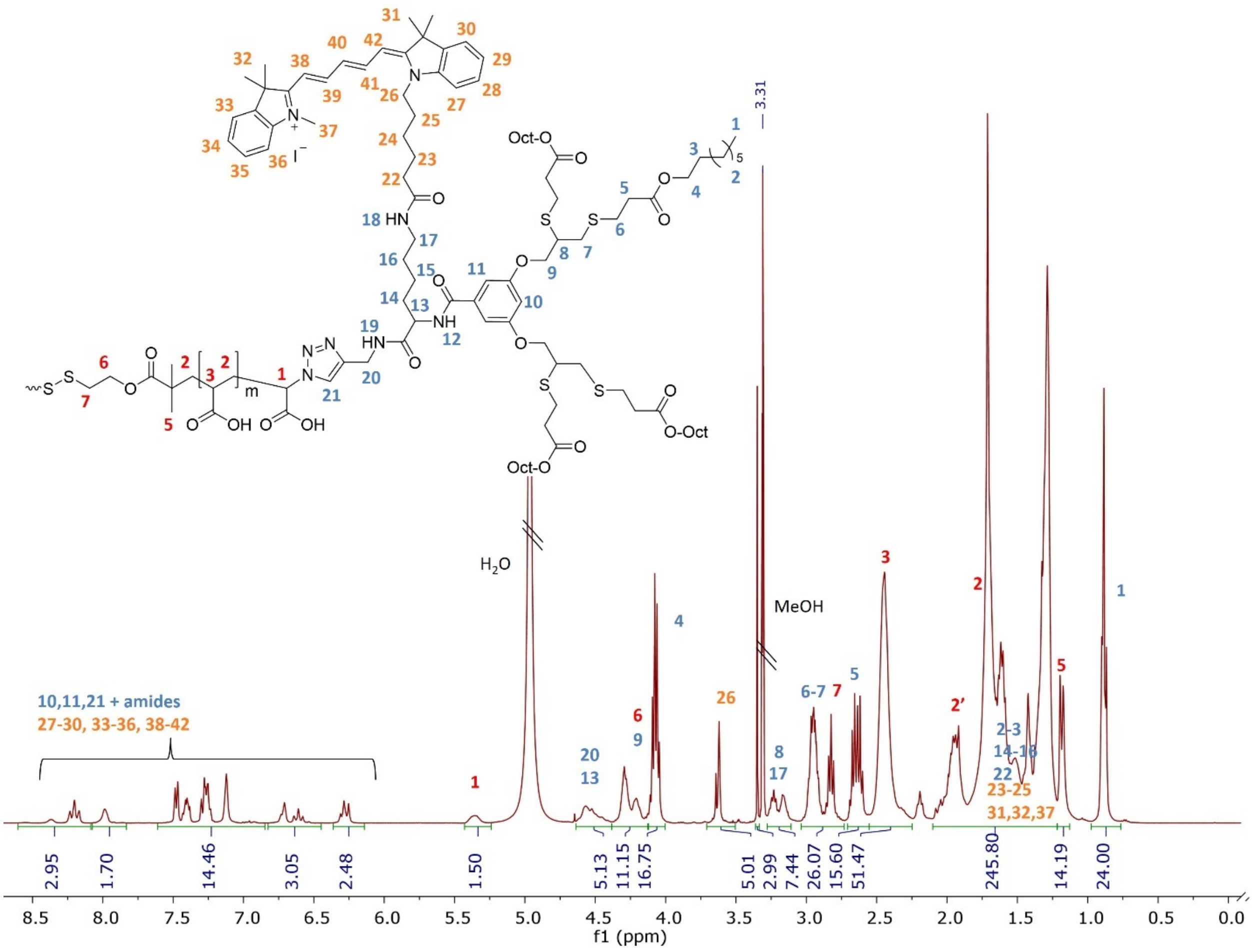

Figure S38: ^{1}H-NMR spectra of SS-PAA-L(Cy5)-dend-4xOct in CD_3OD.

SS-PAA-L(Ac)-dend-4xDec

SS-PtBA-L(Ac)-dend-4xDec was treated with TFA according to the general procedure. ^{1}H-NMR (400 MHz, CD_3OD): see following spectrum and assignments.

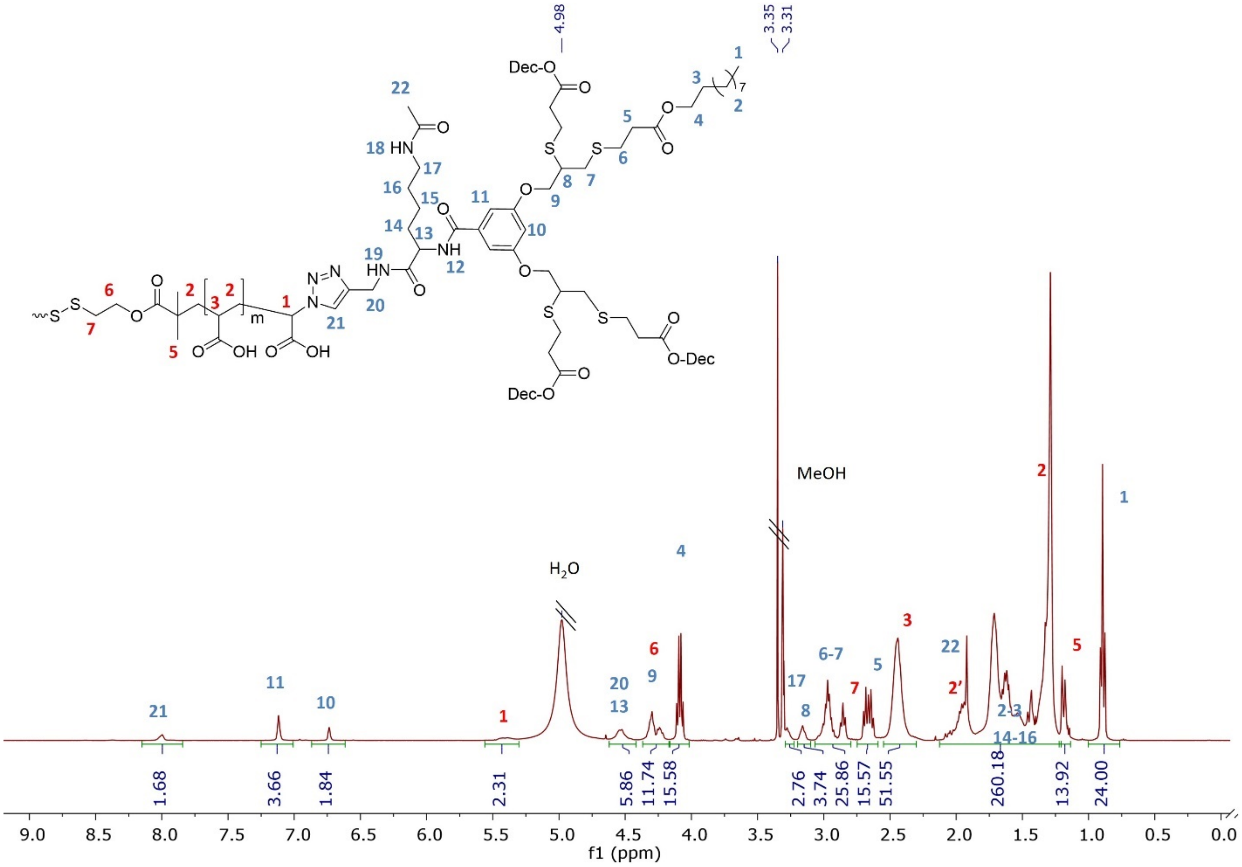

Figure S39: ^{1}H-NMR spectra of SS-PAA-L(Ac)-dend-4xDec in CD_3OD.

SS-PAA-L(Cy3)-dend-4xDec

SS-PtBA-L(Cy3)-dend-4xDec was treated with TFA according to the general procedure. ^{1}H-NMR (400 MHz, CD_3OD): see following spectrum and assignments.

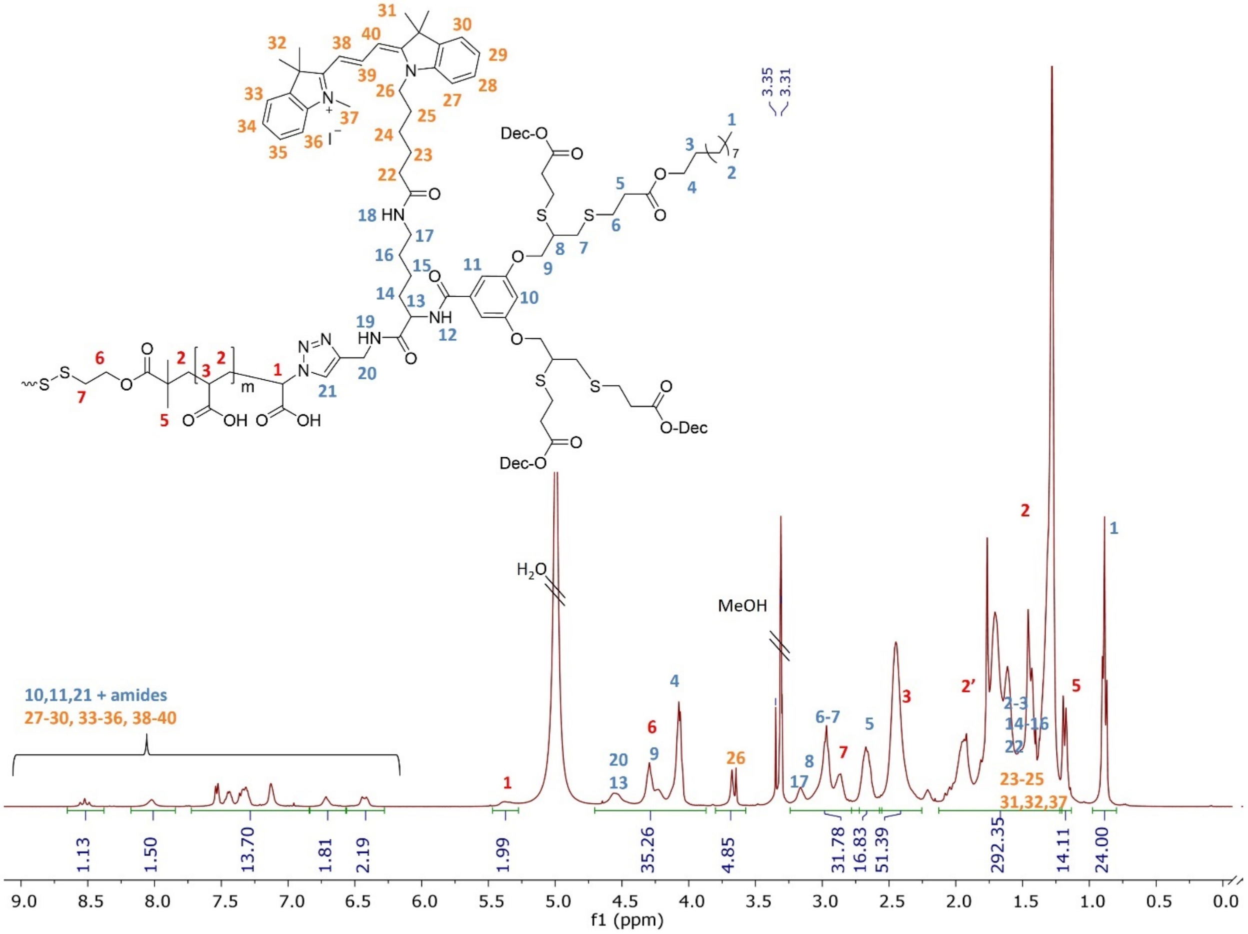

Figure S40: ^{1}H-NMR spectra of SS-PAA-L(Cy3)-dend-4xDec in CD_3OD.

SS-PAA-L(Cy5)-dend-4xDec

SS-PtBA-L(Cy5)-dend-4xDec was treated with TFA according to the general procedure. ^{1}H-NMR (400 MHz, CD_3OD): see following spectrum and assignments.

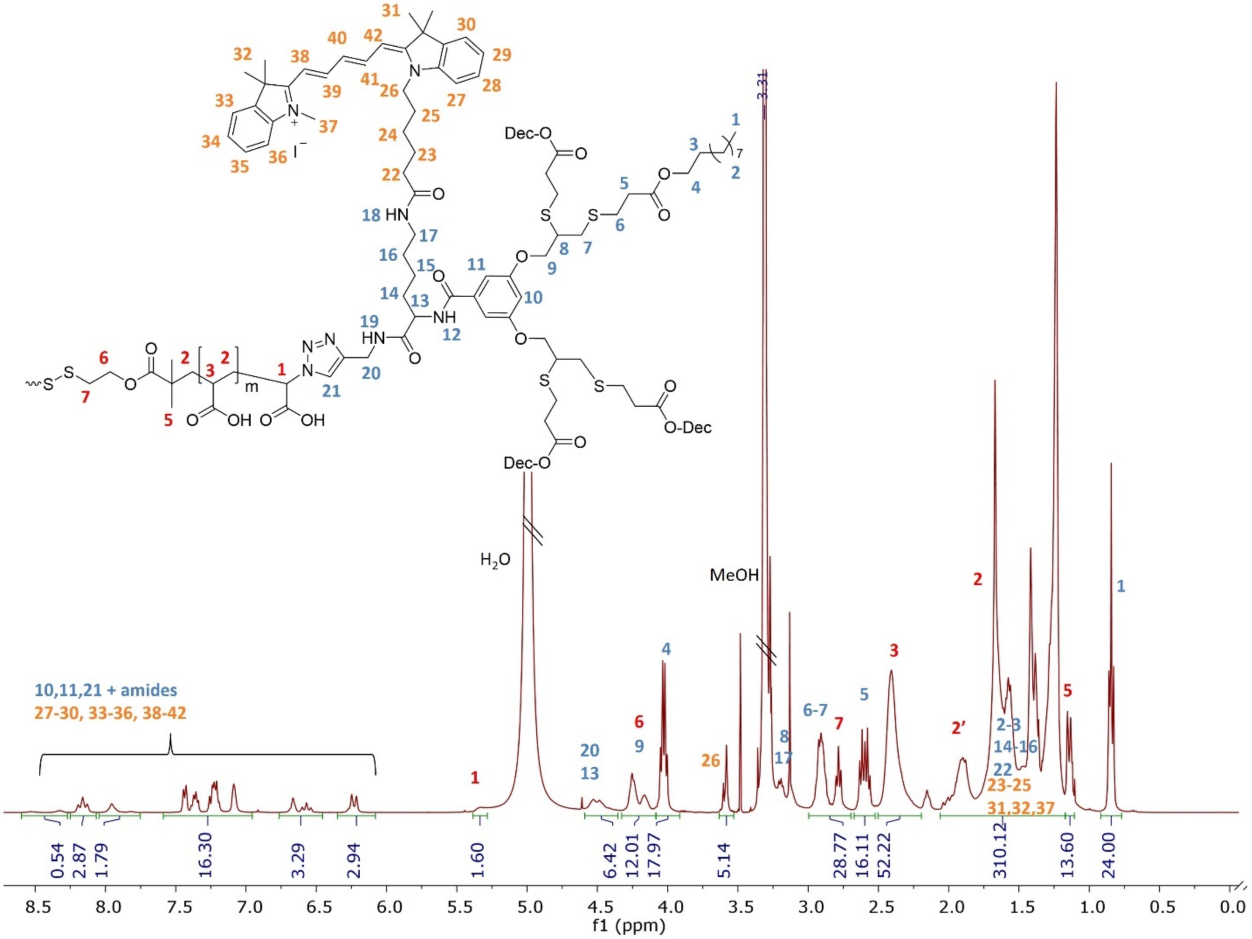

Figure S41: ^{1}H-NMR spectra of SS-PAA-L(Cy5)-dend-4xDec in CD_3OD.

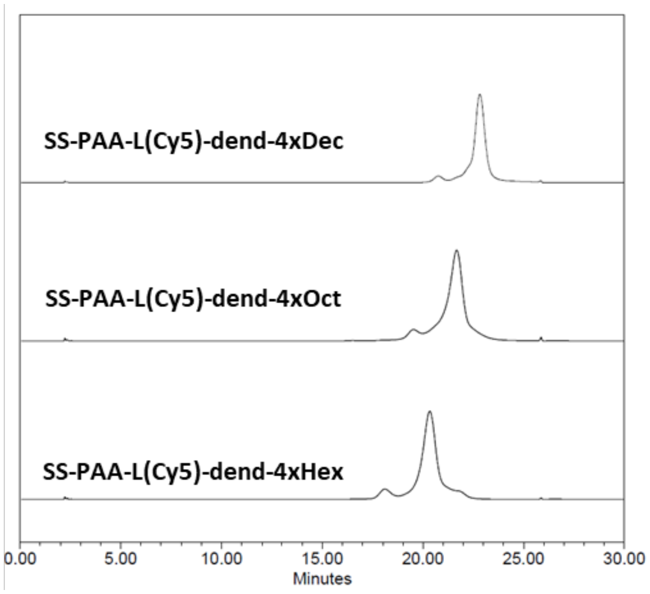

Figure S42: HPLC overlay of Cy5 labelled TBA amphiphiles, taken at 640nm.

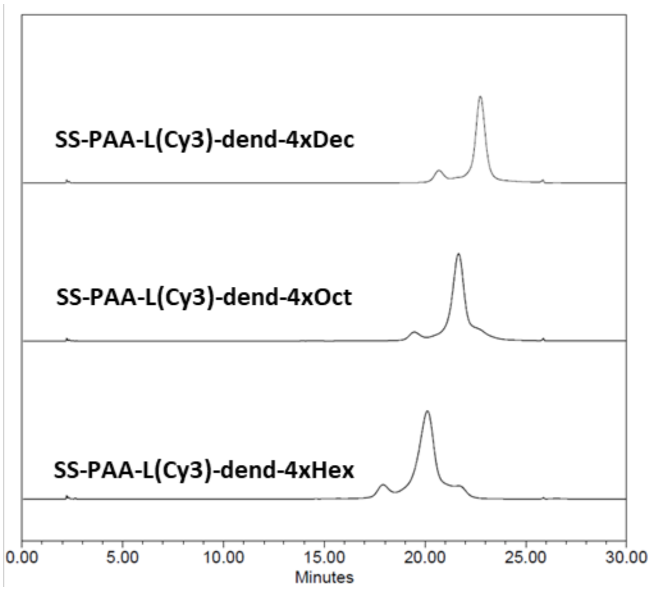

Figure S43: HPLC overlay of Cy3 labelled TBA amphiphiles, taken at 545nm.

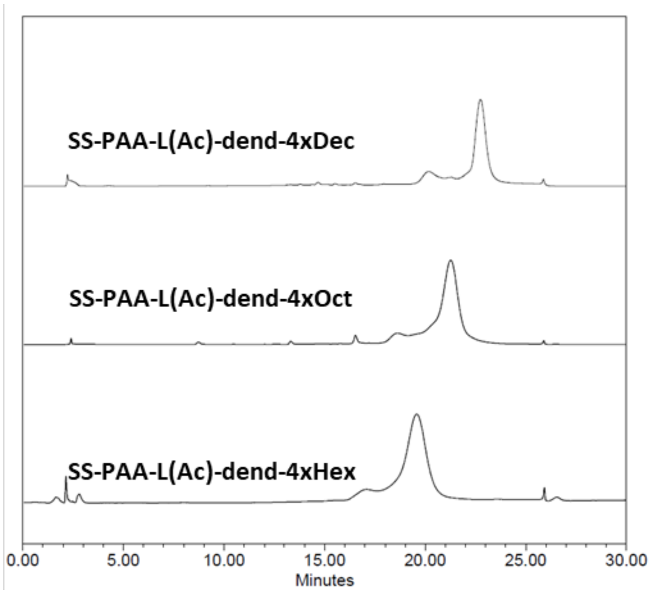

Figure S44: HPLC overlay of Acetyl labelled TBA amphiphiles, taken at 279nm.

<u>Size exclusion chromatography (SEC)</u>

<u>Instrument method:</u>

<u>Instrument:</u> Malvern Viscotek GPCmax

<u>Columns:</u> 2xPSS GRAM 1000Å

<u>Columns temperature:</u> 50°C

<u>Flow rate:</u> 0.5 mL/min

<u>Injection time:</u> 60 min

<u>Injection volume:</u> 50 µL from a 10 mg/ml sample

<u>Diluent + mobile phase:</u> DMF + 25mM NH_4Ac

<u>Needle wash:</u> DMF

<u>Detector:</u> Viscotek VE3580 RI detector

Sample preparation: The Polymer-dendron amphiphiles were directly dissolved in the diluent to give a final concentration of 10 mg/mL and filtered with 0.45 µM PTFE filter.

* Cy labelled and deprotected PAA amphiphiles could not be analyzed by SEC due to column interactions.

* Samples were analyzed using PEG-based calibration curve.

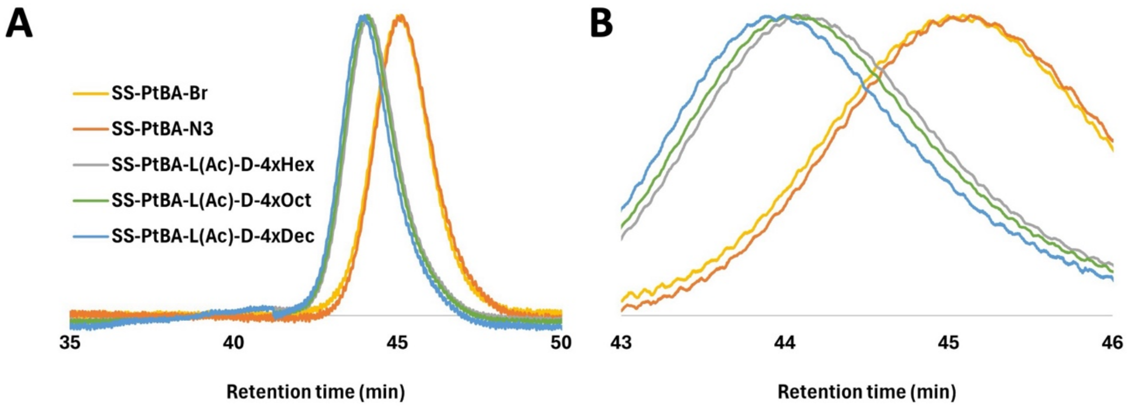

C

Name	Mn_{NMR} (kDa)	Mn_{SEC} (kDa)	Đ
SS-PtBA-Br	6.9	3.9	1.12
SS-PtBA-N3	6.8	3.7	1.13
SS-PtBA-L(Ac)-D-4xHex	9.3	5.2	1.12
SS-PtBA-L(Ac)-D-4xOct	9.5	5.3	1.12
SS-PtBA-L(Ac)-D-4xDec	9.7	5.5	1.13

Figure S45: (A) SEC traces overlay of SS-PtBA-Br, SS-PtBA-N_3, SS-PtBA-L(Ac)-4xHex (TBA-Hex), SS-PtBA-L(Ac)-4xOct (TBA-Oct) and SS-PtBA-L(Ac)-4xDec (TBA-Dec). (B) zoomed-in overlay of A. (C) Mn and Đ values, as calculated by ^{1}HNMR and based on SEC analysis using PEG standards calibration.

Characterization of assembled micellar structures

General protocol for the preparation of TBA and DBA micellar solution

The relevant TBA polymers were dissolved in THF (10% w/w referring to PBS) at the desired formulation ratio (20% labelled and 80% non-labelled amphiphiles). Phosphate buffer saline (PBS, pH=7.4) was added using a syringe pump at a rate of 22.22 µL/min, while stirring, to afford the micellar formation at desired concentration. After the addition was completed, the test tube covered with a KimWipe, and the solution was stirred over night at RT to allow the full evaporation of THF. The solutions were weighted, and if needed, PBS was added upon evaporation.

For the in-situ transition from TBA- to DBA- based micelles, a micellar solution of TBA-X was prepared as mentioned above, then treated with DTT (20 µL from 1M solution in PBS into 1 mL, to yield final DTT concentration of 20 mM), and was incubated for 0.5 hours at 37°C. The full transformation from TBA into DBA amphiphiles was confirmed by HPLC.

HPLC measurements

Instrument: Waters Alliance e2695

Column: Aeris WIDEPORE, C4, 3.6 µm, 150x4.6 mm

Column temperature: 30°C

Sample temperature: 37°C

Solution A: 0.1% $HClO_4$:ACN 95:5v/v

Solution B: 0.1% $HClO_4$:ACN 5:95v/v

Flow rate: 1ml/min

Injection volume: 20 µL

Seal wash: H_2O:MeOH 90:10v/v

Needle wash: MeOH

Detector: Waters 2998 photodiode array detector

Sampling rate: 2 points/sec

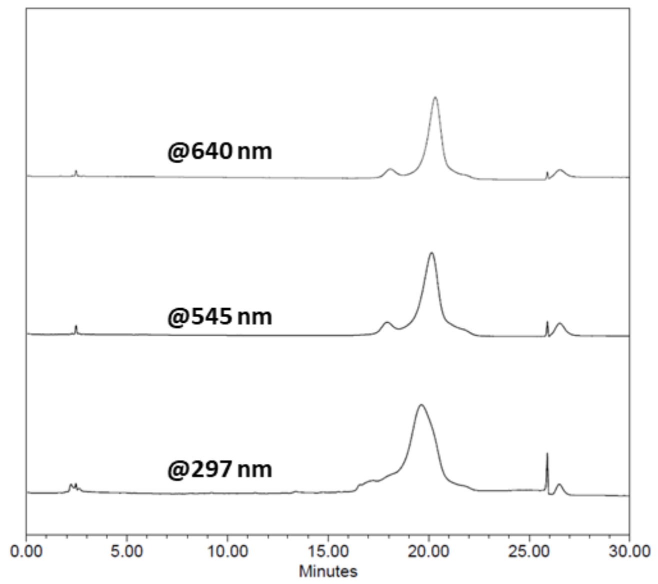

Figure S46: representative HPLC overlay of TBA-Hex experimental mixture with 10% Cy3, 10% Cy5 and 80% acetyl labelled amphiphiles, taken at distinctive wavelengths. All three amphiphiles have an absorbance at 297 nm due to dendritic structure.

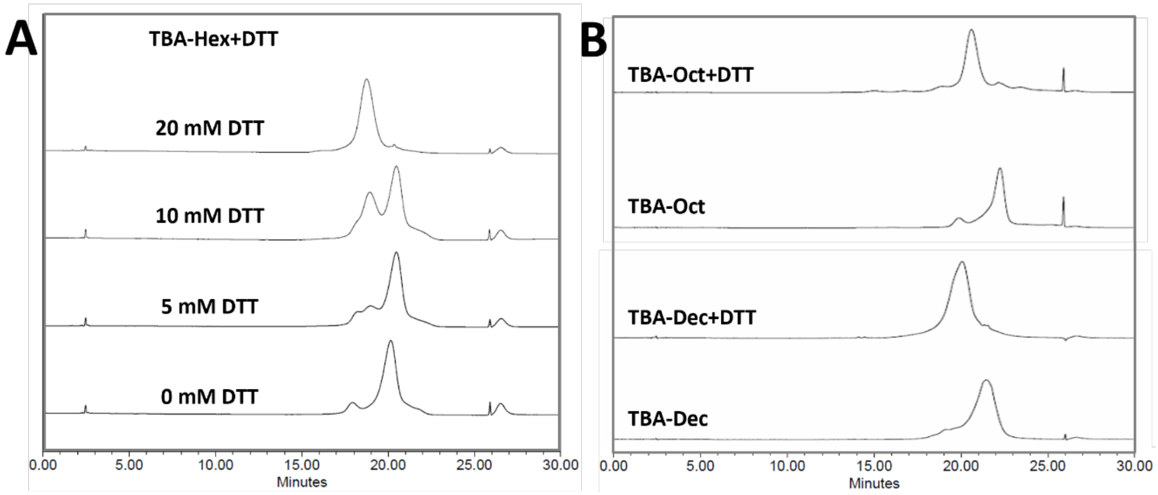

Figure S47: A) representative HPLC overlay of TBA-Hex splitting into DBA-Hex amphiphile upon 10 minutes incubation with different concentrations of reducing agent DTT. B) TBA-Oct and TBA-Dec before and after incubation with 20 mM DTT, confirm full splitting of the amphiphiles under set conditions.

Critical micelles' concentration (CMC)

General procedure of measurement:

Micellar solutions were prepared according to the general protocol, with 50% Cy3 and 50% Cy5 labelled amphiphiles, at a final concentration of 250 µM. Each TBA-X solution was divided into two samples, one of which was treated according to the general procedure for splitting of the TBA amphiphiles into DBAs. Each sample was consecutively diluted by a factor of 1.5 with the diluent to afford a series of 24 samples with decreasing concentration. 150 µL of each sample were loaded onto a 96 well plate and a fluorescence emission scan was performed for each well. To determine the amphiphile's CMC – the FRET signal was monitored by plotting the maximum emission of Cy5 (670 nm), upon the excitation of Cy3 (520 nm), versus the amphiphile's concentration. This procedure was repeated trice for each amphiphile, and mean value is reported as the CMC value and the standard deviation as measurement error.

Instrument method:

Instrument: TECAN Infinite M200Pro

Excitation: 520 nm

Emission intensity scan: 550-750 nm

Step: 5 nm

Number of flashes: 5

Gain: 100

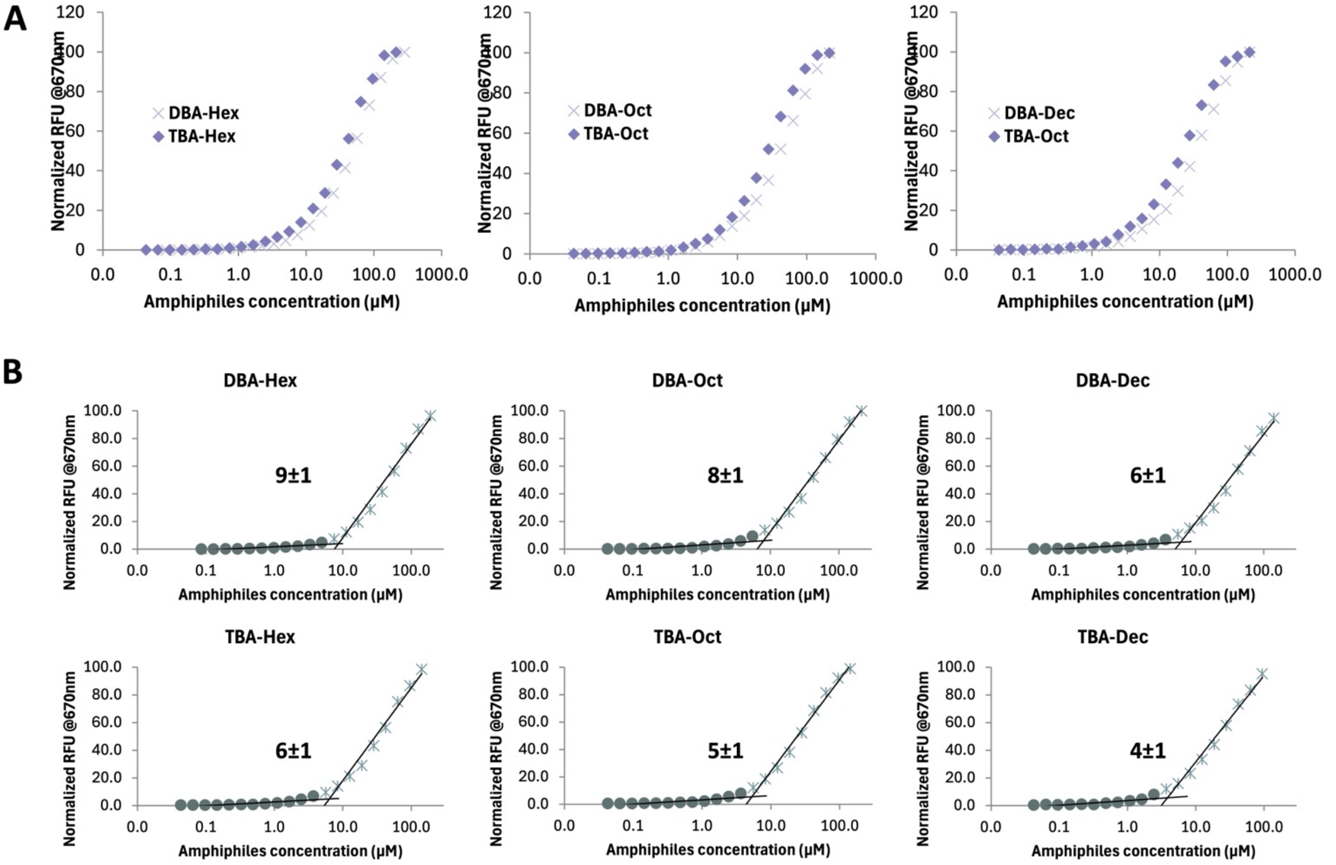

Figure S48: CMC measurements of TBA-X and DBA-X micellar systems.

Dynamic light scattering

All samples for DLS measurements were prepared according to the general protocol, with 20% Cy3 and 80% unlabelled amphiphiles, at a final concentration of 80 µM, and were filtered prior to measurement using 0.45 µm nylon filter.

A

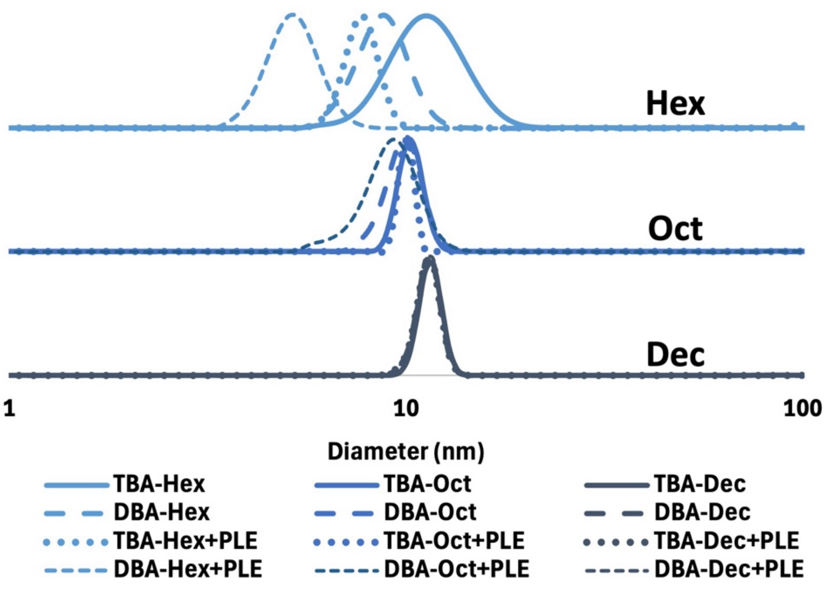

B

	Diameter (nm)	
**Amphiphile**	**t=0**	**t=24h**
TBA-Hex	11±2	8±1
TBA-Oct	10±1	10±1
TBA-Dec	11±1	11±1
DBA-Hex	9±1	5±1
DBA-Oct	10±1	9±1
DBA-Dec	11±1	11±1

Figure S49: (A) size measurements overlay for TBA-X and DBA-X micelles, before and after 24 hours of incubation with PLE. [TBA] = 80 µM, [DTT] = 20 mM, [PLE] = 0.1 µM. (B) Analyzed D_H values of the solutions.

TEM imaging

Sample preparation:

Micellar solutions were prepared according to the general protocol, with 10% Cy3, 10% Cy5 and 80% unlabelled amphiphiles, at a final concentration of 80 µM. 30 µL of the hybrid solution were deposited onto carbon coated copper grids. The excessive solvent of the droplet was wiped away using a solvent-absorbing filter paper after 1 minute and the sample grids were left to dry in air at RT for 8 hours. Then, grids were inspected in transmission electron microscope (TEM), operated at 120 kV (JEM-1400Plus).

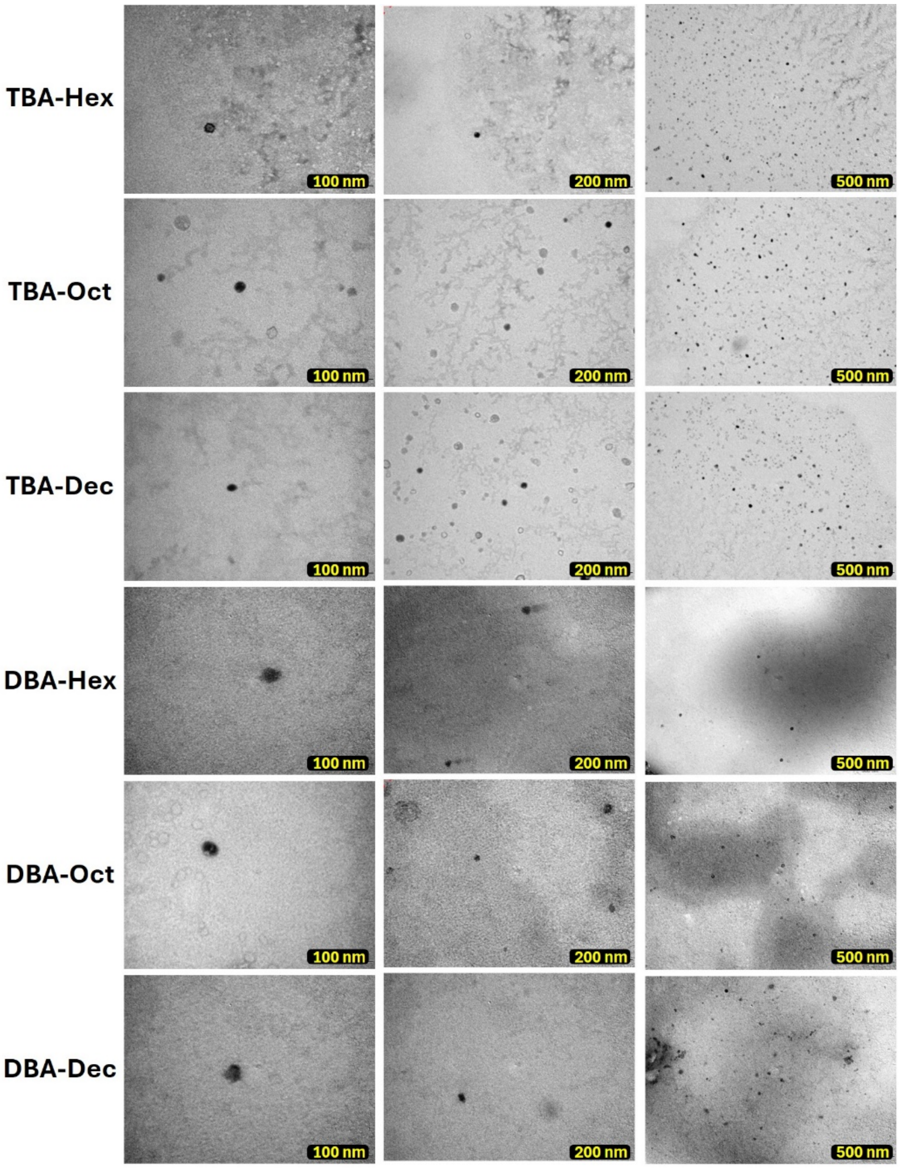

Figure S50: TEM images of TBA-X and DBA-X micellar solutions.

Enzymatic degradation experiments and FRET mixing essays:

Experimental protocol for enzymatic degradation:

Micellar solutions were prepared according to the general protocol, with 10% Cy3, 10% Cy5 and 80% unlabelled amphiphiles, at a final concentration of 80 µM. Splitting of the amphiphiles from TBA do DBA was conducted as mentioned in the general protocol. PLE was added (20 µL into 1 mL, to yield final PLE concentration of 0.1 µM) and degradation was followed at 37°C by monitoring the area under the peak of the parent amphiphiles. Each experiment was conducted trice and the reported values in each time point are the mean valued, and the standard deviation is the error.

Control experiments:

(i) Effect of DTT on enzymatic activity and end-group stability:

Micellar solution of non-splittable hybrid "*mPEG$_{5k}$-D-(Hep)$_4$*" (as synthesized and reported [6]), with comparative dendritic structure and esterase-responsive end-groups, was prepared at a final concentration of 80 µM. DTT, PLE or both were added as mentioned above, and degradation was followed at 37°C by monitoring the area under the peak of the parent amphiphile (297 nm).

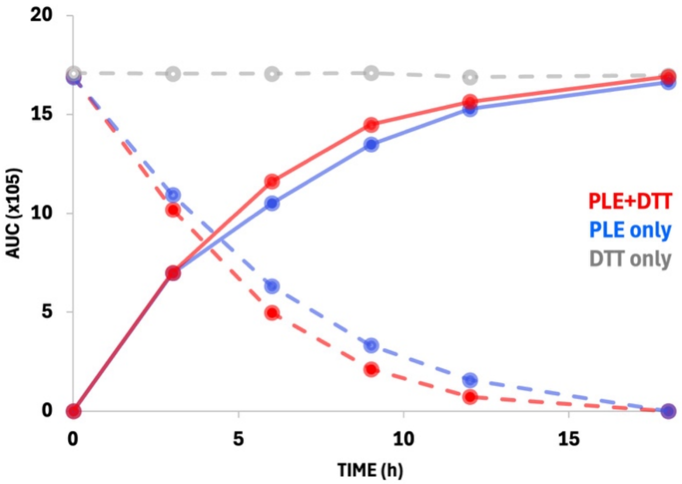

Figure S51: Degradation profile of enzyme responsive hybrid mPEG5k-4xHep: calculated AUC of the parent amphiphile (dashed line) and hydrolyzed polymer (solid line) after treatment with DTT (gray), PLE (blue) or a combination of both (red). [amphiphile] = 80 µM, [DTT] = 20 mM, [PLE] = 0.1 µM.

(ii) Amphiphiles' stability towards hydrolysis:

Micellar solutions were prepared according to the general protocol, with 10% Cy3- and 10% Cy5-labelled amphiphiles, and 80% unlabelled micelles, at a final concentration of 80 µM. Splitting of the amphiphiles from TBA do DBA was conducted as mentioned in the general protocol. Amphiphiles' degradation profile in the absence of the activating enzyme PLE was followed at 37°C by monitoring the area under the peak of the parent amphiphile.

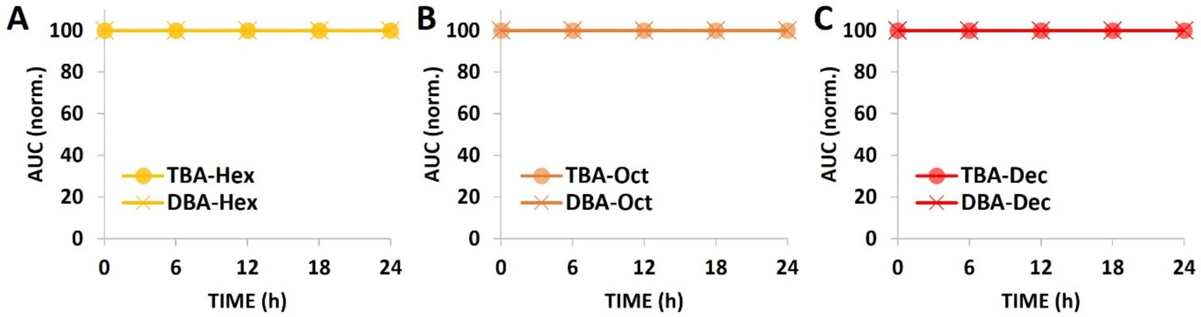

Figure S52: Amphiphiles' stability profiles in the absence of PLE as obtained by HPLC for (A) Hex-, (B) Oct- and (C) Dec-based micelles (TBA-X (circle) and DBA-X (crossed)). [TBA] = 80 µM, [DTT] = 20 mM.

Experimental protocol for FRET based mixing assay:

Micellar solutions were prepared according to the general protocol, with 20% of either Cy3 or Cy5 labelled amphiphiles, and 80% unlabelled micelles, at a final concentration of 80 µM. Splitting of the amphiphiles from TBA do DBA was conducted as mentioned in the general protocol. Micellar solutions were mixed gently in a quartz cuvette, at 1:1 v/v ratio, and the change in emission spectra was measured at the fluorometer (37°C, λ_{ex}=520 nm). Each experiment was conducted trice and the reported values in each time point are the mean valued, and the standard deviation is the error.

FRET signal stability over time:

Mixed-micellar solutions were prepared according to the general protocol, with 10% Cy3- and 10% Cy5-labelled amphiphiles, and 80% unlabelled micelles, at a final concentration of 80 µM. Splitting of the amphiphiles from TBA do DBA was conducted as mentioned in the general protocol. The change in Cy3 and Cy5 emission intensity (570 nm and 670 nm, respectively) was recorded by the fluorometer (37°C, λ_{ex}=520 nm).

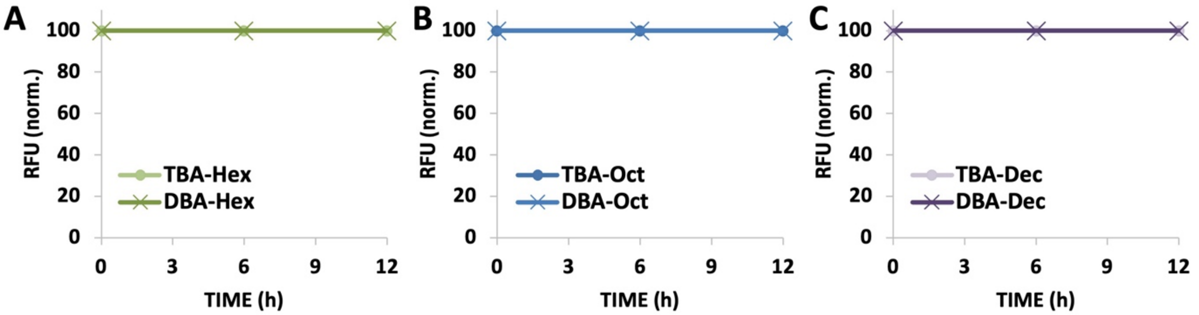

Figure S53: FRET signal stability over time – Cy5 emission (670 nm) for (A) Hex-, (B) Oct- and (C) Dec-based mixed-micelles (TBA-X (circle) and DBA-X (crossed)). [TBA] = 80 µM, [DTT] = 20 mM, λ_{ex}=520 nm*.*

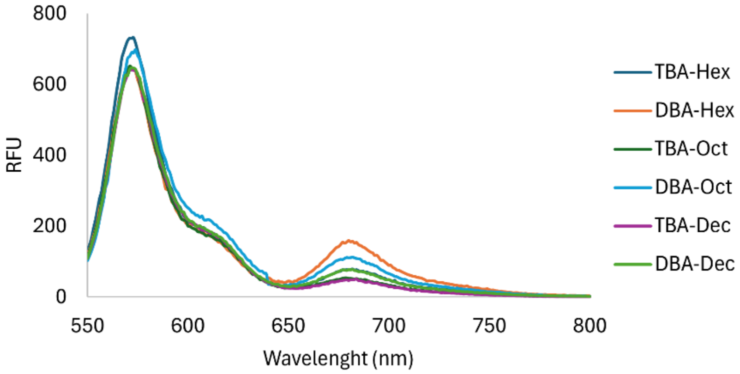

Figure S54: Emission spectra of labelled micelles at t=0, upon the mixing of two micellar populations as part of the FRET-based mixing assays. [amphiphile] = 80 µM, [DTT] = 20 mM. λ_{ex}=520 nm*.*

References:

(1) Slor, G.; Tevet, S.; Amir, R. J. Stimuli-Induced Architectural Transition as a Tool for Controlling the Enzymatic Degradability of Polymeric Micelles. *ACS Polymers Au* **2022**, *2* (5), 380–386. https://doi.org/10.1021/ACSPOLYMERSAU.2C00023.

(2) Harnoy, A. J.; Buzhor, M.; Tirosh, E.; Shaharabani, R.; Beck, R.; Amir, R. J. Modular Synthetic Approach for Adjusting the Disassembly Rates of Enzyme-Responsive Polymeric Micelles. *Biomacromolecules* **2017**, *18* (4), 1218–1228. https://doi.org/10.1021/ACS.BIOMAC.6B01906/ASSET/IMAGES/LARGE/BM-2016-01906T_0014.JPEG.

(3) Slor, G.; Olea, A. R.; Pujals, S.; Tigrine, A.; De La Rosa, V. R.; Hoogenboom, R.; Albertazzi, L.; Amir, R. J. Judging Enzyme-Responsive Micelles by Their Covers: Direct Comparison of Dendritic Amphiphiles with Different Hydrophilic Blocks. *Biomacromolecules* **2021**, *22* (3), 1197–1210. https://doi.org/10.1021/ACS.BIOMAC.0C01708/ASSET/IMAGES/LARGE/BM0C01708_0009.JPEG.

(4) Harnoy, A.; Papo, N.; Slor, G.; Amir, R. Mixing End Groups in Thiol-Ene/Yne Reactions as a Simple Approach toward Multienzyme-Responsive Polymeric Amphiphiles. *Synlett* **2018**, *29* (19), 2582–2587. https://doi.org/10.1055/s-0037-1611340.

(5) Slor, G.; Olea, A. R.; Pujals, S.; Tigrine, A.; De La Rosa, V. R.; Hoogenboom, R.; Albertazzi, L.; Amir, R. J. Judging Enzyme-Responsive Micelles by Their Covers: Direct Comparison of Dendritic Amphiphiles with Different Hydrophilic Blocks. *Biomacromolecules* **2021**, *22* (3), 1197–1210. https://doi.org/10.1021/acs.biomac.0c01708.

(6) Tevet, S.; Amir, R. J. Hydrophobicity as a Tool for Programming Sequential Mesophase Transitions of Enzyme-Responsive Polymeric Amphiphiles. *J Mater Chem B* **2024**, *12* (45), 11685–11695. https://doi.org/10.1039/D4TB01587H.
